# Mimicking Nature in Reshaping the Triterpene Skeleton: Synthesis of a Class of Unnatural Oleanane Derivatives

**DOI:** 10.1021/acs.orglett.5c02231

**Published:** 2025-07-10

**Authors:** Edoardo di Biase, Ernesto Gargiulo, Daniela Imperio, Giuseppina Chianese, Diego Caprioglio, Hawraz Ibrahim M. Amin, Orazio Taglialatela-Scafati, Alberto Minassi

**Affiliations:** ‡ Department of Pharmaceutical Sciences, University of Piemonte Orientale, Largo Donegani 2, 28100 Novara, Italy; § Department of Pharmacy, 9307 University of Naples Federico II, Via Montesano 49, 80131 Naples, Italy; ∥ Department for Sustainable Development and Ecological Transition, University of Piemonte Orientale, Piazza Sant’Eusebio 5, 13100 Vercelli, Italy; ⊥ Department of Chemistry, Università degli Studi di Pavia, Via Taramelli 12, 27100 Pavia, Italy

## Abstract

The possibility of duplicating the efficiency and selectivity of natural processes under laboratory conditions remains a significant challenge for organic chemists. In this letter, we demonstrated that it is possible to introduce pinpoint modifications and to reshape the terpene skeleton of easily available oleananes in a biomimetic fashion. Furthermore, aromatization of the A ring of the triterpene system is reported here for the first time, mimicking what occurs exclusively in nature in sediments.

## Graphical abstract

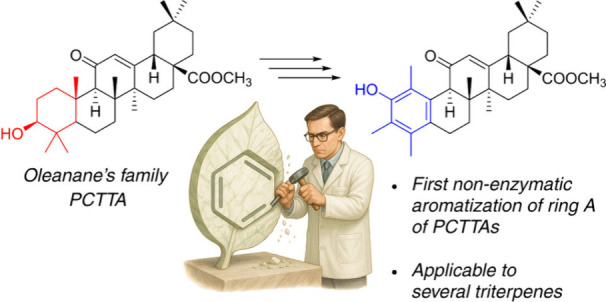

Terpenes are probably the most structurally diverse family of natural products. This complexity derives from very simple biosynthetic routes through a “cyclase phase” in which cation-triggered polyene cyclization yields a myriad of derivatives typically containing multiple fused rings and stereocenters.^1^

Pentacyclic triterpene acids (PCTTAs) are a class of secondary terpenoids resulting from the oxidative cyclization of squalene, with oleanane, ursane, and lupane being their biogenetically earliest and most widespread skeletal types.^2^ While the first two are characterized by a pentacyclic 6/6/6/6/6 ring system diverging only for the position of the methyl groups C-29 and C-30, the lupane skeleton is characterized by the contraction of ring E with a typical 6/6/6/6/5 system (Scheme 1). All of the modifications of the carbon architecture occur via Wagner–Meerwein transpositions catalyzed by a single enzyme that is able to transform squalene oxide into the final products.^3^

**Scheme 1:**
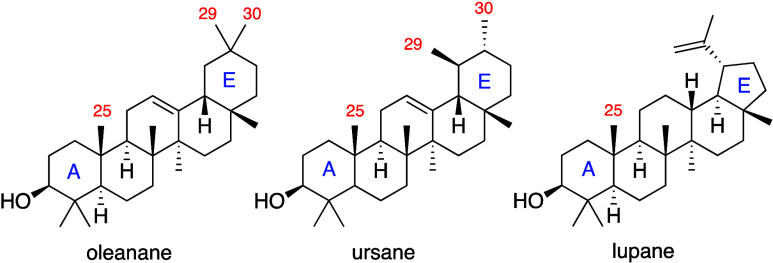
Pentacyclic Triterpene Acid Skeletal Types

PCTTAs have attracted considerable attention due to their intrinsic biological properties, and through the decoration of the triterpene core, new and more powerful derivatives have been discovered.^4^ Although decoration has been an excellent strategy for expanding the pharmacological potential of PCTTAs, it is nevertheless confined to a restricted number of basic structures, On the contrary, the discovery of new scaffolds holds a much higher potential to unveil new bioactivities.^5^

Our research group recently studied the photoreactivity of PCTTAs in order to find a strategy for manipulation of the carbon skeleton of easily available triterpenic acids. In particular, we described a new photochemical pathway for the direct transformation of Δ^1^-3-oxo-oleanolic acids into the justicane analogues in a biomimetic class-interconversion process.^6^ In search of new ideas to fertilize the field and inspired by the seminal works of Stork and Burgstahler,^7^ Eschenmoser,^8^ and Johnson^9^ about the domestication of polyene cyclizations, we started a new project focused on the introduction of pinpoint modifications on the A ring of easily accessible oleanane derivatives. In an attempt to copy nature, obtaining a new class of unnatural oleananes, we reasoned about a strategy to shift the methyl group C-25 from C-10 to C-1 in a stereospecific manner. To induce the rearrangement, functional groups facilitating this migration were needed, with an allylic alcohol and epoxyketone identified as possible solutions to our requirements. The synthesis of derivatives **2** and **3** started from the methyl ester of oleanolic acid **1**, the most widespread and easily available oleanane. Allylic derivative **2** was synthesized in two steps, in which the enone system was first installed by oxidation with IBX,^10^ and subsequently, the ketone moiety was selectively reduced under Luche’s conditions.^11^ Alternatively, derivative **3** was obtained from compound **4** by epoxidation in an alkaline medium. Lastly, compounds **2** and **3** were treated with BF_3_ etherate. Compound **2** reacted instantaneously, resulting in the formation of single product **5** in 85% yield, while compound **3** required longer times, yielding the two isomeric products **6** and **7** in 40 and 16% yields, respectively (Scheme 2).

**Scheme 2:**
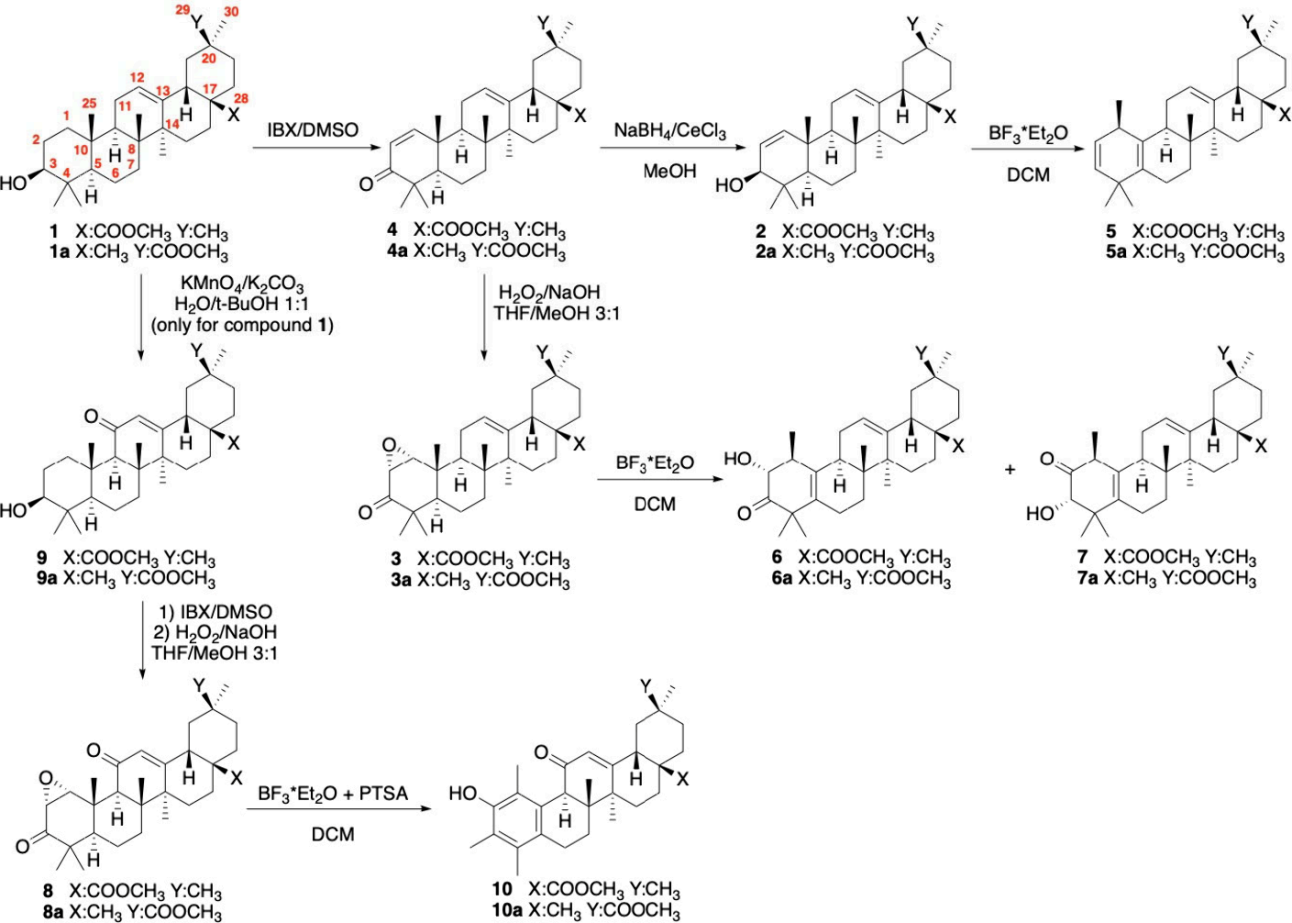
Synthesis of the Rearranged Products **5**, **5a**, **6**, **6a**, **7**, **7a**, **10**, and **10a**

The chemical structures of compounds **5**–**7** were confirmed by MS and through analysis of their 1D and 2D NMR spectra. For compound **5**, the doublet at δ_H_ 1.0, assigned to H_3_-25, was part of a spin system including H-1, H-2, and H-3. The placement of the double bonds Δ^2^ and Δ^5(10)^ was secured by the HMBC correlations of H_3_-23/H_3_-24 with C-4 and with sp^2^ C-3 and C-5 and of H_3_-25 with sp^2^ C-10. The β orientation of Me-25 was inferred by the NOESY cross-peaks of both H_3_-25 and H_3_-26 with H-12β. Parallel results were obtained for compounds **6** and **7** with the obvious exception that H-1 was coupled to an oxymethine in compound **6**, while H_3_-25 gave HMBC correlation to a ketone carbonyl in compound **7**.

Compound **5** could be derived by the loss of the hydroxy group at C-3, induced by Lewis acid coordination, with the subsequent migration of the double bond between C-2 and C-3, followed by the suprafacial methyl shift and the final regiospecific elimination of H-5 with the installation of the second double bond between C-5 and C-10. The reaction could be concerted, and it can be favored by the antiperiplanar alignment of the migrating methyl group at C-10 and the leaving hydrogen at C-5 (Scheme 3a). On the other hand, compounds **6** and **7** are regioisomers resulting from the epoxide opening followed by the stereospecific methyl shift and the subsequent installation of the double bond. In particular, intermediate **11** (the analogue of compound **6**), once formed, could be in equilibrium with enediol **12** that after hydrolysis furnished the regioisomeric mixture (Scheme 3b).

**Scheme 3:**
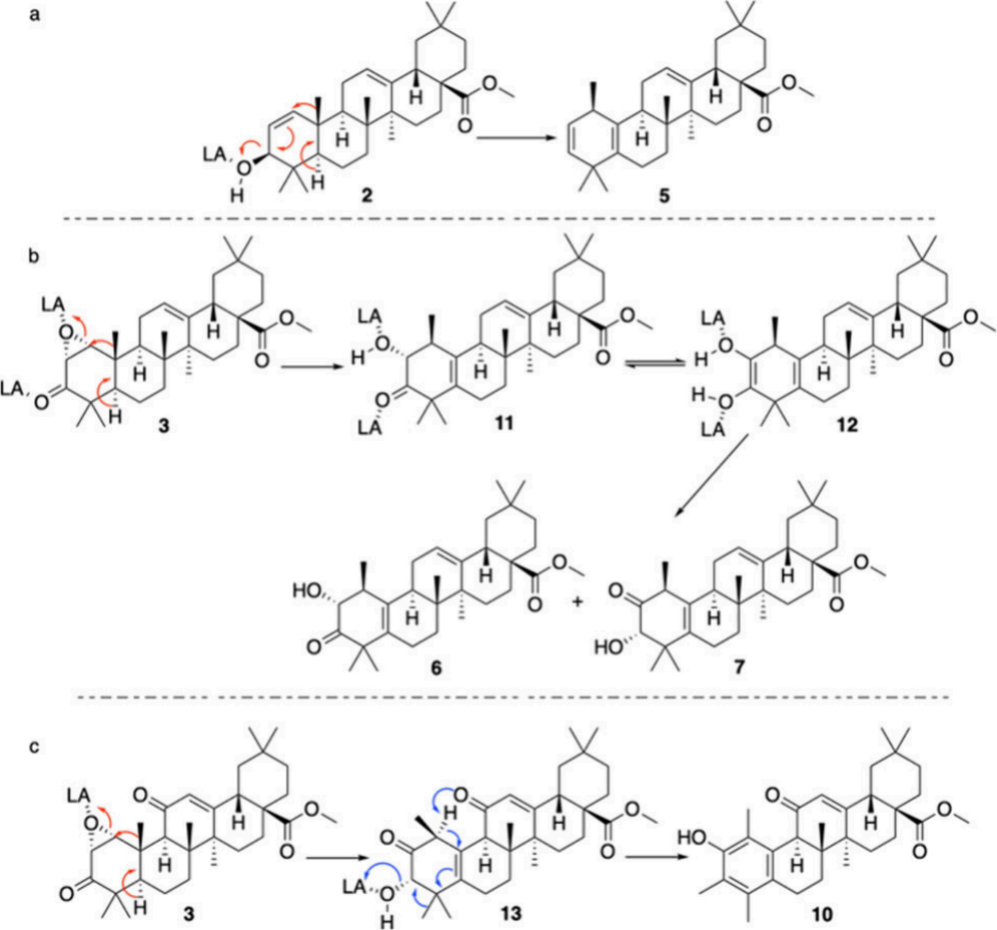
(a) Proposed Mechanism for the Synthesis of Compound **5**, (b) Proposed Mechanism for the Synthesis of Compounds **6** and **7**, and (c) Proposed Mechanism for the Synthesis of Compound **10**

Since 11-oxo-oleananes have important biological activities, as exemplified by glycyrrhetinic acid, we decided to expand the scope of the strategy, exploring the reactivity of 1,2-epoxy-3–11-dioxo oleanolic derivative **8**. This was synthesized by starting from compound **1** in three steps. An allylic oxidation mediated by KMnO_4_/K_2_CO_3_ was used to add ketone at C-11, followed by the installation of the enone moiety on ring A and subsequent selective epoxidation of the disubstituted double bond. Compound **8** then reacted with BF_3_ etherate in dry DCM, and to our surprise, aromatic derivative **10** was obtained in 15% yield (Scheme 2).

Compound **10** was fully characterized by MS and NMR. The aromatization of ring A was unambiguously deduced from the ^13^C NMR resonances of C-1 to C-5 and C-10, while the placement of the methyl groups on the aromatic ring was inferred by the network of HMBC correlations and by the key NOESY cross-peaks of H_3_-23 with H_3_-24 and of H_3_-25 with H-9.

In order to improve the yield of the reaction, a mixture of Lewis and Bronsted acids was used: the use of 3 mol/equiv of PTSA resulted in a marked increase in the yield of compound **10**, up to 45%. In comparison of the structures of compounds **8** and **3**, it is clear that, to obtain the aromatization of ring A, the presence of a ketone group at C-11 is of crucial importance. In addition, a protic acid is needed to promote the displacement of the hydroxyl group. After the formation of intermediate **13** (the analogue of compound **7**), the loss of the proton at C-1, favored by an anchimeric assistance of the keto moiety at C-11, could be the switch inducing cascading rearrangements that start with the formation of a new double bond between C-1 and C-10. This induces the migration of the pre-existing double bond to C-4 and C-5 and the simultaneous shift of one of the methyl groups from C-4 to C-3, and the final loss of the protonated hydroxyl moiety at C-3 leads to the aromatic derivative (Scheme 3c). To corroborate this hypothesis, compound **3** was treated with a mixture of BF_3_ etherate and PTSA without furnishing any aromatic derivative.

To test the generality of the process, we selected another member of the oleanane family: glycyrrhetinic acid. Its methyl ester derivatives **2a**, **3a**, and **8a** were reacted under optimized conditions and satisfactorily gave the homologous products (**5a**, **6a**, **7a**, and **10a**) previously described for oleanolic acid (Scheme 2).

Our results demonstrate that it is possible to mimic nature by introducing pinpoint modifications in the carbon architecture of the oleanane skeleton. By using the appropriate functional groups, the stereoselective migration of methyl group C-25 from C-10 to C-1 can be achieved in moderate to excellent yields, providing a new class of natural–unnatural oleananes.

In the enzymatic process catalyzed by aromatase, the oxidative demethylation of C-25 is needed to obtain the aromatization of ring A in estrogens. On the other hand, the aromatization of ring A of triterpenoids has also been described in sediments at the earlier stages of diagenesis,^12–15^ where it probably involves microbially mediated processes. This paper describes for the first time a non-enzymatic aromatization of ring A of a triterpenic acid, mimicking in a flask what nature does with enzymes. The reaction seems general for oleananes, and most likely, it can be extended to ursanes and other triterpenoidic scaffolds. However, this requires specific structural features to happen. The aromatized products are obtained through a cascade pathway involving the migration of two methyl groups, the installation of two new double bonds, and the loss of a molecule of water. This strategy can pave the way to the preparation of a new class of unnatural PCTTAs, whose unpredictable pharmacological profiles could open the possibility of the discovery of new bioactivities.

## Supplementary Material



## Data Availability

The data underlying this study are available in the published article and its Supporting Information.

## Notes

Experimental procedures, characterization data for all products, and NMR spectra of compounds **2**/**2a**, **3a**, **8**/**8a**, **5**/**5a**, **6**/**6a**, **7**/**7a**, and **10**/**10a** (PDF)
