# Supplementary material for: Mimicking Nature in Reshaping the Triterpene Skeleton: Synthesis of a Class of Unnatural Oleanane Derivatives
Source: Org Lett. 2025 Jul 10;27(28):7639–42. doi: 10.1021/acs.orglett.5c02231 (PMC12281560; doi:10.1021/acs.orglett.5c02231)

Supplementary Information

Mimicking nature in reshaping the triterpene skeleton: synthesis of a class of unnatural oleanane derivatives

Edoardo di Biase,[‡] Ernesto Gargiulo,[‡] Daniela Imperio, Giuseppina Chianese, Diego Caprioglio, Hawraz Ibrahim M. Amin, Orazio Taglialatela-Scafati* and Alberto Minassi*

Edoardo di Biase, Diego Caprioglio, Alberto Minassi: Department of Pharmaceutical Sciences, University of Piemonte Orientale, L.go Donegani 2, 28100 Novara, Italy

Daniela Imperio: Department for Sustainable Development and Ecological Transition, University of Piemonte Orientale, P.zza St. Eusebio 5, 13100 Vercelli, Italy

Hawraz Ibrahim M. Amin: Department of Chemistry, Università degli Studi di Pavia, Via Taramelli 12, 27100 Pavia, Italy

Ernesto Gargiulo, Giuseppina Chianese, Orazio Taglialatela-Scafati: Department of Pharmacy, University of Naples Federico II, Via Montesano 49, 80131 Naples, Italy

1. **Experimental** ... 4
   1.1. **General experimental procedures** ... 4
   1.2. **^{1}H NMR spectrum of compound 2 (400 MHz, $CDCl_3$)** ... 13
   1.3. **^{13}C NMR spectrum of compound 2 (400 MHz, $CDCl_3$)** ... 13
   1.4. **^{1}H NMR spectrum of compound 8 (400 MHz, $CDCl_3$)** ... 14
   1.5. **^{13}C NMR spectrum of compound 8 (400 MHz, $CDCl_3$)** ... 14
   1.6. **^{1}H NMR spectrum of compound 2a (400 MHz, $CDCl_3$)** ... 15
   1.7. **^{13}C NMR spectrum of compound 2a (400 MHz, $CDCl_3$)** ... 15
   1.8. **^{1}H NMR spectrum of compound 3a (400 MHz, $CDCl_3$)** ... 16
   1.9. **^{13}C NMR spectrum of compound 3a (400 MHz, $CDCl_3$)** ... 16
   1.10. **NMR spectrum of compound 8a (400 MHz, $CDCl_3$)** ... 17
   1.11. **^{13}C NMR spectrum of compound 8a (400 MHz, $CDCl_3$)** ... 17
   1.12. **NMR spectrum of compound 5 (400 MHz, $CDCl_3$)** ... 18
   1.13. **^{13}C NMR spectrum of compound 5 (400 MHz, $CDCl_3$)** ... 18
   1.14. **COSY NMR spectrum of compound 5 (400 MHz, $CDCl_3$)** ... 19
   1.15. **2D HSQC NMR spectrum of compound 5 (400 MHz, $CDCl_3$)** ... 20
   1.16. **2D HMBC NMR spectrum of compound 5 (400 MHz, $CDCl_3$)** ... 20
   1.17. **2D NOESY NMR spectrum of compound 5 (400 MHz, $CDCl_3$)** ... 21
   1.17 **^{1}H NMR spectrum of compound 6 (400 MHz, $CDCl_3$)** ... 22
   1.18 **^{13}C NMR spectrum of compound 6 (400 MHz, $CDCl_3$)** ... 22
   1.19 **COSY NMR spectrum of compound 6 (400 MHz, $CDCl_3$)** ... 23
   1.23 **^{1}H NMR spectrum of compound 7 (400 MHz, $CDCl_3$)** ... 25
   1.24 **^{13}C NMR spectrum of compound 7 (400 MHz, $CDCl_3$)** ... 25
   1.25 **COSY NMR spectrum of compound 7 (400 MHz, $CDCl_3$)** ... 26
   1.26 **HSQC NMR spectrum of compound 7 (400 MHz, $CDCl_3$)** ... 26
   1.27. **HMBC NMR spectrum of compound 7 (400 MHz, $CDCl_3$)** ... 27
   1.28 **NOESY NMR spectrum of compound 7 (400 MHz, $CDCl_3$)** ... 27
   1.29 **^{1}H NMR spectrum of compound 10 (400 MHz, $CDCl_3$)** ... 28
   1.30 **^{13}C NMR spectrum of compound 10 (400 MHz, $CDCl_3$)** ... 28
   1.35. **^{1}H NMR spectrum of compound 5a (400 MHz, $CDCl_3$)** ... 31
   1.36 **^{13}C NMR spectrum of compound 5a (400 MHz, $CDCl_3$)** ... 31
   1.41. **^{1}H NMR spectrum of compound 6a (400 MHz, $CDCl_3$)** ... 34
   1.42. **^{13}C NMR spectrum of compound 6a (400 MHz, $CDCl_3$)** ... 34
   1.43 **COSY NMR spectrum of compound 6a (400 MHz, $CDCl_3$)** ... 35
   1.44 **HSQC NMR spectrum of compound 6a (400 MHz, $CDCl_3$)** ... 35

1.45 . **HMBC NMR spectrum of compound 6a (400 MHz, $CDCl_3$)**......36

1.47. **^{1}H NMR spectrum of compound 7a (400 MHz, $CDCl_3$)**......37

1.48. **^{13}C NMR spectrum of compound 7a (400 MHz, $CDCl_3$)**......37

1.49 **COSY NMR spectrum of compound 7a (400 MHz, $CDCl_3$)**......38

1.50 **COSY NMR spectrum of compound 7a (400 MHz, $CDCl_3$)**......38

1.51. **HMBC NMR spectrum of compound 7a (400 MHz, $CDCl_3$)**......39

1.52 **NOESY NMR spectrum of compound 7a (400 MHz, $CDCl_3$)**......39

1.53 **^{1}H NMR spectrum of compound 10a (400 MHz, $CDCl_3$)**......40

1.54 **^{13}C NMR spectrum of compound 10a (400 MHz, $CDCl_3$)**......40

1.55 **COSY NMR spectrum of compound 10a (400 MHz, $CDCl_3$)**......41

1.56 **HSQC NMR spectrum of compound 10a (400 MHz, $CDCl_3$)**......41

1.57 **HMBC NMR spectrum of compound 10a (400 MHz, $CDCl_3$)**......42

1.58 **NOESY NMR spectrum of compound 10a (400 MHz, $CDCl_3$)**......42

1. Experimental

1.1. General experimental procedures

NMR Spectroscopy: Optical rotations were measured on an Anton Paar Polarimeter MCP 100 at 20 °C. Infrared spectra were recorded on a FT-IR Bruker Alpha II spectrometer with absorption maxima (νmax) recorded in wavenumbers (cm^{-1}). ^{1}H (400 MHz) and ^{13}C (100 MHz) NMR spectra were measured on a Bruker Avance spectrometer (Bruker, Billerica, MA, USA). Chemical shifts are referenced to the residual solvent signal ($CDCl_3$: δ_H 7.26 ppm, δ_C 77.0 ppm). Homonuclear ^{1}H connectivities were determined by COSY (correlation spectroscopy) experiments. Through-space ^{1}H connectivities were evidenced using a NOESY (nuclear Overhauser enhancement spectroscopy) experiment with a mixing time of 300 ms. One-bond heteronuclear $^{1}H-^{13}C$ connectivities was determined by the HSQC (heteronuclear single quantum correlation) experiment: two- and three-bond $^{1}H-^{13}C$ connectivities by gradient-HMBC (heteronuclear multiple bond correlation) experiments optimized for a $^{2,3}J$ of 8 Hz. Structural assignments were made with additional information from gCOSY, gHSQC, and gHMBC experiments. The NMR spectra of all the other compounds (**2**/**2a**, **3**/**3a** and **8**/**8a**) were recorded using a Bruker Avance Neo 400 MHz spectrometer. Chemical shifts are reported in parts per million (ppm) relative to the residual solvent. A Q-Exactive Plus UHMR Hybrid Quadrupole Orbitrap™ Mass Spectrometer (Waltham, MA, USA) was employed for mass spectrometry. The spectra were recorded by infusion into the ESI source using MeOH as the solvent. Commercially available reagents and solvents were purchased from Aldrich, TCI and Fluorochem and were used without further purification. Column chromatography was performed on silica gel (Merck Kieselgel 60, 230−400 mesh ASTM). Thin-layer chromatography (TLC) was carried out on 5 × 20 cm plates with a layer thickness of 0.25 mm (Merck silica gel 60 F254). Compounds **1**,[1] **3**,[2] **4**[2], **4a**[3] and **9a**[4] have previously been reported.

Chromatographic purifications: HPLC separations were performed on an Agilent instrument, using a Knauer 1800 apparatus equipped with a refractive index detector using Luna (5 μm Silica 250 × 4.6 mm) column (Phenomenex, Torrance, CA, USA) and a Rheodyne injector. Thin-layer chromatography (TLC) was performed on plates coated with silica gel 60 (F254 Merck, 0.25 mm).

Chemicals and solvents were from Merck Life Science S.r.l. and were used without any further purification unless stated otherwise.

Synthesis of Δ¹-oleanoic acid methyl ester (2): To a solution of compound **4** (745 mg, 1.60 mmol; 1 equiv./mol) in 50 mL MeOH, $CeCl_3$*7 H_2O (3,6 g, 9.58 mmol; 6 equiv./mol) and $NaBH_4$ (362 mg, 9.58 mmol; 6 equiv./mol) were sequentially added. The reaction was stirred at rt for 3h, quenched with H_2SO_4 (2N sol.) and extracted with EtOAc. The organic phase was dried over Na_2SO_4 and evaporated at reduced pressure. The crude was purified over silica gel (PE/EtOAc 9:1 as eluent, isocratic) to give compound **2** (659 mg, 88%) as white solid. $[\alpha]^{20}_D$ +104.4 (*c* 0.7, $CHCl_3$); m.p.: 204 °C; IR (KBr) 2948, 2913, 2864, 1726, 1468, 1382, 1199, 1024, 651; ^1H NMR (400 MHz, $CDCl_3$) 5.80 (dd, 1H, *J* = 10.2, 2.6 Hz), 5.31 (m, 2H, H-2 and H-12), 3.90 (d, 1H, *J* = 7.6 Hz), 3.43 (s, 3H), 2.88 (dd, 1H, *J* = 13.9, 3.0 Hz), 1.15 (s, 3H), 1.08 (s, 3H), 1.02 (s, 3H), 0.95 (s, 3H), 0.91 (s, 3H), 0.83 (s, 3H), 0.77 (s, 3H), (partial ^1H assignment, only signals amenable to a first-order analysis are reported); ^{13}C NMR (101 MHz, $CDCl_3$) δ 178.2, 144.1, 137.8, 125.4, 122.1, 77.3, 53.4, 51.5, 46.7, 45.7, 44.2, 41.8, 41.3, 39.9, 39.0, 37.0, 33.8, 33.1, 33.0, 32.3, 30.6, 27.6, 27.4, 26.0, 23.8, 23.6, 23.0, 17.7, 17.57, 17.56, 17.51; HRMS (ESI) m/z: $[M + H]^+$ Calcd for $C_{31}H_{49}O_3^+$, 469.3676; Found 469.3673.

*Synthesis of (1α,2α)-1,2-epoxy-3,11-dioxo-oleanoic acid methyl ester (**8**)*: To a solution of oleanolic acid methyl ester **1** (1 g, 2.12 mmol; 1 equiv./mol) in 12 mL H_2O/t-BuOH (1:1), K_2CO_3 (1.76 g, 12.75 mmol; 6 equiv./mol) and $KMnO_4$ (1.34 g, 8.50 mmol; 4 equiv./mol) were sequentially added. The reaction was stirred at 40°C (oil bath) for 48h. The reaction mixture was then cooled down at rt and quenched with brine and extracted with EtOAc. The organic phase was washed with Na_2SO_3 (sat. sol.), dried over Na_2SO_4 and evaporated at reduced pressure. The crude was purified over silica gel (PE/EtOAc 7:3 as eluent, isocratic) to give compound **9** (564 mg, 55%) as white solid (the NMR data are in agreement with that reported in literature).[5] To a solution of compound **9** (564 mg,1.17 mmol; 1 equiv./mol) in 16 mL EtOAc/DMSO (3:1), IBX (1.64 g, 5.85 mmol; 5 equiv./mol) and fluorobenzene (5%) were sequentially added. The reaction was stirred at 110°C (oil bath) for 12h, cooled down at rt and filtered over celite pad and washed with EtOAc. The organic phase was washed with Na_2SO_3 (sat. sol.), dried over Na_2SO_4 and evaporated at reduced pressure. The crude was

purified over silica gel (PE/EtOAc 8:2 as eluent, isocratic) to give the dienone (2.1 gr, 75%) as a white solid. To a solution of the latter (488mg, 0.947 mmol; 1 equiv./mol) in 21.5 mL THF, MeOH (6 mL), NaOH (2 M sol.; 6 mL) and H_2O_2 (30%; 3.5 mL) were sequentially added. The reaction was stirred at rt for 6h, diluted with brine solution and extracted with EtOAc. The organic phases were dried over Na_2SO_4 and evaporated at reduced pressure. The crude was purified over silica gel (PE/EtOAc 9:1 as eluent, isocratic) to give compound **8** (2.07 gr, 95%) as white solid. $[\alpha]^{20}_{D}$ +213.1 (*c* 0.7 $CHCl_3$); m.p.: 197 °C; IR (KBr) 2949, 2866, 1725, 1697, 1650, 1467, 1385, 1195, 1032, 1010, 883, 627; ^{1}H NMR (400 MHz, $CDCl_3$) 5.76 (brs, 1H), 4.57 (d, 1H, *J* = 4.7 Hz), 3.66 (s, 3H), 3.40 (d, 1H, *J* = 4.7 Hz), 3.07 (dd, 1H, *J* = 13.8, 3.7 Hz), 2.92 (s, 1H), 1.44 (s, 3H), 1.19 (s, 3H), 1.12 (s, 3H), 1.03 (s, 3H), 0.97 (s, 3H), 0.96 (s, 3H), 0.95 (s, 3H), (partial ^{1}H assignment, only signals amenable to a first-order analysis are reported); ^{13}C NMR (101 MHz, $CDCl_3$) δ 212.4, 199.2, 177.4, 170.3, 127.4, 64.7, 57.3, 54.6, 51.9, 46.2, 45.6, 44.8, 44.8, 44.3, 43.7, 41.8, 38.5, 32.8, 31.8, 31.5, 23.5, 23.4, 22.8, 20.9, 19.0, 18.0, 15.6; HRMS (ESI) m/z: $[M + H]^+$ Calcd for $C_{31}H_{45}O_5^+$, 497.3261; Found 497.3253.

Synthesis of Δ^1-olean-29-oic acid methyl ester (2a): To a solution of compound **4a** (412 mg, 0.88 mmol; 1 equiv./mol) in 25 mL MeOH, $CeCl_3$*7 H_2O (1.98 g, 5.3 mmol; 6 equiv./mol) and $NaBH_4$ (200 mg, 5.3 mmol; 6 equiv./mol) were sequentially added. The reaction was stirred at rt for 3h, quenched with a solution of H_2SO_4 (2N sol.) and extracted with EtOAc. The organic phase was dried over Na_2SO_4 and evaporated at reduced pressure. The crude was purified over silica gel (PE/EtOAc 9:1 as eluent, isocratic) to give compound **2a** (375 mg; 91%) as white solid. $[\alpha]^{20}_{D}$ +191.1 (*c* 0.5 $CHCl_3$); m.p.: 198 °C; IR (KBr) 3356, 2963, 2946, 2906, 2871, 2842, 1722, 1465, 1451, 1216, 1158, 1025, 822, 802; ^{1}H NMR (400 MHz, $CDCl_3$) 5.82 (dd, 1H, *J* = 10.3, 2.2 Hz), 5.31 (m, 2H), 3.91 (t, 1 H, *J* = 2.2 Hz), 3.70 (s, 3H), 1.15 (s, 3H), 1.14 (s, 3H), 1.11 (s, 3H), 1.03 (s, 3H), 1.01 (s, 3H), 0.85 (s, 3H), 0.80 (s, 3H), partial ^{1}H assignment, only signals amenable to a first-order analysis are reported); ^{13}C NMR (101 MHz, $CDCl_3$) 177.6, 144.6, 137.9, 125.4, 122.3, 69.2, 53.3, 51.5, 51.5, 48.2, 45.0, 44.3, 42.7, 41.7, 40.4, 38.9, 38.4, 37.0, 36.2, 33.0, 31.9, 31.3, 28.5, 28.5, 28.1, 27.4, 26.9, 26.0, 23.9, 17.9, 17.4; HRMS (ESI) m/z: $[M + H]^+$ Calcd for $C_{31}H_{49}O_3^+$, 469.3676; Found 469.3674.

Synthesis of (1α,2α)-1,2-epoxy-3-oxo-olean-29-oic acid methyl ester (3a): To a solution of

compound **4a** (413 mg, 0.88 mmol; 1 equiv./mol) in 18 mL THF, MeOH (5 mL), NaOH (2 M sol.; 5 mL) and H_2O_2 (30%; 3 mL) were sequentially added. The reaction was stirred at rt for 6h, diluted with brine and extracted with EtOAc. The organic phase weas dried over Na_2SO_4 and evaporated at reduced pressure. The crude was purified over silica gel (PE/EtOAc 95:5 as eluent, isocratic) to give compound **3a** (382 mg, 90%) as white solid. $[\alpha]^{20}_D$ +203.5 (*c* 0.5 $CHCl_3$); m.p.: 148 °C; IR (KBr) 2948, 2906, 2868, 1728, 1699, 1453, 1381, 1214, 1153, 1085, 878; ^1H NMR (400 MHz, $CDCl_3$) δ 5.36 (t, 1H, *J*= 3.4 Hz), 3.71 (s, 3H), 3.53 (d, 1H, *J* = 4.6 Hz), 3.39 (d, 1H, *J* = 4.6 Hz), 1.23 (s, 3H), 1.16 (s, 3H), 1.13 (s, 3H), 1.12 (s, 3H), 1.04 (s, 3H), 1.03 (s, 3H), 0.81 (s, 3H), partial ^1H assignment, only signals amenable to a first-order analysis are reported); ^{13}C NMR (101 MHz, $CDCl_3$) δ ^{13}C NMR (101 MHz, CDCl3) δ 13C NMR (101 MHz, CDCl3) δ 212.7, 177.6, 144.9, 121.7, 63.9, 56.9, 51.5, 48.3, 46.0, 44.8, 44.2, 42.7, 41.8, 40.6, 40.0, 38.4, 38.3, 32.0, 32.0, 31.3, 28.5, 28.2, 28.0, 26.9, 26.1, 25.7, 23.9, 20.9, 18.9, 17.1, 15.0; HRMS (ESI) m/z: $[M + H]^+$ Calcd for $C_{31}H_{47}O_4^+$, 483.3468; Found 483.3469.

*Synthesis of (1α,2α)-1,2-epoxy-3,11-dioxo-olean-29-oic acid methyl ester (**8a**)*: To a solution of compound **9a** (650 mg, 1.34 mmol; 1 equiv./mol) in 16 mL EtOAc/DMSO (3:1), IBX (1.88 g, 6.70 mmol; 5 equiv./mol) and fluorobenzene (5%) were sequentially added. The reaction was stirred at 110°C (oil bath) for 12h, cooled down at rt, filtered over celite pad and washed with EtOAc. The organic phase was washed with Na_2SO_3 (sat. sol.), dried over Na_2SO_4 and evaporated at reduced pressure. The crude was purified over silica gel to give the dienone (450 mg, 71%) as a white solid. To a solution of the latter (450 mg, 0.947 mmol; 1 equiv./mol) in 21.5 mL THF, MeOH (6 mL), NaOH (2 M sol.; 6 mL) and H_2O_2 (30%; 3.5 mL) were sequentially added. The reaction was stirred at rt for 6h, diluted with Brine solution and extracted with EtOAc. The organic phases were dried over Na_2SO_4 and evaporated at reduced pressure. The crude was purified over silica gel (PE/EtOAc 9:1 as eluent, isocratic) to give compound **8a** (446 mg, 95%) as white solid. $[\alpha]^{20}_D$ +252.3 (*c* 0.8 $CHCl_3$); m.p.: 241 °C; IR (KBr) 2963, 2947, 2919, 2874, 1724, 1701, 1650, 1610, 1216, 1152, 864; ^1H NMR (400 MHz, $CDCl_3$) 5.79 (brs, 1H), 4.53 (d, 1H, *J* = 4.7 Hz), 3.72 (s, 3H), 3.41 (d, 1H, *J* = 4.7 Hz), 2.94 (s, 1H), 1.45 (s, 3H), 1.23 (s, 3H), 1.18 (s, 3H), 1.17 (s, 3H), 1.14 (s, 3H), 1.04 (s, 3H), 0.85 (s, 3H) (partial ^1H assignment, only signals amenable to a first-order analysis are reported); ^{13}C

NMR (101 MHz, $CDCl_3$) δ 212.4, 199.1, 176.9, 170.9, 128.1, 64.7, 57.3, 54.7, 51.8, 48.5, 45.6, 45.1, 44.8, 44.0, 43.5, 41.1, 38.4, 37.7, 31.9, 31.7, 31.1, 28.6, 28.3, 27.9, 26.6, 26.3, 23.3, 20.9, 18.76, 18.17, 15.8; HRMS (ESI) m/z: $[M + H]^+$ Calcd for $C_{31}H_{45}O_5^+$, 497.3261; Found 497.3255.

General procedure to obtain compounds 5 and 5a: To a solution of allylic alcohol **2**/**2a** (1 equiv./mol) in dry DCM cooled at 0°C, BF_3*Et_2O (3.5 equiv./mol) was added. The reaction was stirred at 0°C for 10 min. The reaction mixture was quenched with $NaHCO_3$ (sat. sol.), extracted with EtOAc, dried over Na_2SO_4 and evaporated at reduced pressure. The crude was purified over silica gel to give compound **5** (85%)/**5a** (72%)

General procedure to obtain compounds 6, 7, 6a and 7a: to a solution of epoxyketone **3**/**3a** (1 equiv./mol) in dry DCM cooled at 0°C, BF_3*Et_2O (6 equiv./mol) was added. The reaction was stirred at 0°C for 12h. The reaction mixture was quenched with $NaHCO_3$ (sat. sol.), extracted with EtOAc, dried over Na_2SO_4 and evaporated at reduced pressure. The crude was purified over silica gel (PE/EtOAc 95:5; PE/EtOAc 8:2 as eluent, isocratic) to give compound **6** (40%)/**6a** (35%); **7** (16%)/**7a** (18%).

General procedure to obtain compounds 10 and 10a: to a solution of epoxydiketone **8**/**8a** (1 equiv./mol) in dry DCM cooled at 0°C, PTSA (2 equiv./mol) and BF_3*Et_2O (6 equiv./mol) were sequentially added. The reaction was stirred at 0°C for 24h. The reaction mixture was quenched with $NaHCO_3$ (sat. sol.), extracted with EtOAc, dried over Na_2SO_4 and evaporated at reduced pressure. The crude was purified over silica gel (PE/EtOAc 8:2 as eluent, isocratic) to give compound **10** (45%)/**10a** (38%).

*1-methyl-2,5-dien-oleanoic acid methyl ester (**5**)*: yellow solid. $[\alpha]^{20}_D$ +84.7 (*c* 0.8 $CHCl_3$); m.p.: 128 °C; ^1H NMR ($CDCl_3$, 400 MHz) *δ* 5.73 (dd, 1H, *J* = 9.5, 5.2, H-2), 5.49 (d, 1H, *J* = 9.5, H-3), 5.43 (m, 1H, H-12), 3.63 (s, 3H, -OCH_3-28), 2.92 (dd, 1H, *J* = 14.4, 3.7, H-18), 2.61 (quint., 1H, *J* = 5.1, 25.2, H-1), 2.55 (m, 1H, H-9), 2.21 (ovl, 2H, H_2-11), 2.1 (ovl, 1H, H-16a), 2.00 (ovl, 2H, H-16b, H-6a), 1.74 (ovl, 1H, H-19a), 1.72 (ovl, 1H, H-22a), 1.67 (ovl, 1H, H-6b), 1.62 (ovl, 4H, H_2-7, H_2-16), 1.52 (ovl, 1H, H-22b), 1.38 (ovl, 1H, H-21a), 1.32 (ovl, 2H, H_2-15), 1.22 (ovl, 2H, H-19b, H-21b), 1.15 (s, 3H, H_3-27), 1.09 (d, 3H, *J* = 5.1, H_3-25), 1.08 (s, 6H, H_3-23, H_3-24), 0.93 (s, 3H, H_3-29), 0.91 (s, 3H, H_3-30), 0.74 (s, 3H, H_3-26). ^{13}C NMR ($CDCl_3$, 100 MHz) *δ* 178.3 (C-28), 143.2 (C-

13), 136.1 (C-3), 135.5 (C-5), 133.1 (C-10), 131.2 (C-2), 123.4 (C-12), 51.5 (OCH_3), 47.4 (C-17), 45.2 (C-19), 42.6 (C-18), 41.0 (C-14), 39.4 (C-9), 37.9 (C-8), 36.4 (C-4), 34.0 (C-21), 33.4 (C-1), 33.1 (C-30), 32.3 (C-22), 30.7 (C-20), 30.1 (C-23), 29.3 (C-24), 28.9 (C-7), 28.3 (C-11), 28.1 (C-15), 24.9 (C-27), 24.3 (C-25), 23.5 (C-29), 23.3 (C-6), 21.4 (C-16), 16.4 (C-26). HRMS (ESI) m/z: $[M + Na]^+$ Calcd for $C_{31}H_{46}O_2Na^+$ 473.3390; Found 473.3385.

*(2α)-2-hydroxy-1-methyl-3-oxo-5-dien-oleanoic acid methyl ester (**6**)*: Compound **6** was dissolved in chloroform and purified via HPLC on Knauer apparatus using a Phenomenex Luna 5 μm Silica 250 × 4.6 mm column, using a mixture of hexane/ethyl acetate = 90:10. The flow rate was set at 1 mL/min, obtaining compound **6** in the pure state (t_R 24.1 min) as a yellow solid. $[\alpha]_D$ = + 259.3 (*c* 0.08, $CHCl_3$). m.p.: 148 °C; ^1H NMR ($CDCl_3$, 400 MHz) δ 5.44 (dd, 1H, *J* = 4.9, 2.6, H-12), 3.76 (dd, 1H, *J* = 8.8, 3.6, H-2), 3.63 (s, 3H, $-OCH_3$-28), 2.94 (dd, 1H, *J* = 14.4, 3.7, H-18), 2.53 (ovl, 1H, H-1), 2.49 (m, 1H, H-9), 2.23 (m, 2H, H_2-11), 2.22 (m, 1H, H-6a), 2.21 (s, 1H, 2-OH), 2.01 (m, 1H, H-16a), 1.97 (ovl, 1H, H-6b), 1.92 (m, 1H, H-11a), 1.73 (m, 1H, H-19a), 1.71 (m, 1H, H-22a), 1.67 (ovl, 1H, H-16b), 1.57 (ovl, 2H, H-7a, H-15a), 1.47 (dd, 1H, *J* = 5.5, 2.3, H_2-7b), 1.35 (d, 1H, *J* = 4.1, H_2-21a), 1.31 (ovl, 1H, H-15b), 1.30 (s, 3H, H_3-23), 1.22 (ovl, 1H, H-22b), 1.21 (ovl, 1H, H-19b), 1.17 (s, 6H, H_3-24, H_3-27), 0.94 (s, 3H, H_3-30), 0.93 (s, 3H, H_3-29), 0.90 (d, 3H, *J* = 7.1, H_3-25), 0.72 (s, 3H, H_3-26). ^{13}C NMR ($CDCl_3$, 100 MHz) δ 214.0 (C-3), 178.2 (C-28), 143.7 (C-13), 135.0 (C-5), 130.9 (C-10), 122.8 (C-12), 78.5 (C-2), 51.5 (OCH_3), 47.4 (C-17), 46.0 (C-4), 45.2 (C-19), 42.6 (C-18), 40.9 (C-14), 40.4 (C-9), 39.7 (C-1), 37.5 (C-8), 34.0 (C-21), 33.1 (C-29), 32.2 (C-22), 30.7 (C-20), 28.0 (C-7, C-15), 27.2 (C-23), 26.9 (C-11), 24.7 (C-24, C-27), 23.5 (C-30), 23.2 (C-16), 20.7 (C-6), 18.2 (C-25), 15.3 (C-26). HRMS (ESI) m/z: $[M + Na]^+$ Calcd for $C_{31}H_{46}O_4Na$ 505.3289; Found 505.3285.

(3α)-3-hydroxy-1-methyl-2-oxo-5-dien-oleanoic acid methyl ester (7): white solid. $[\alpha]^{20}_D$ +173.0 (*c* 0.5 $CHCl_3$); m.p.: 143 °C; ^1H NMR ($CDCl_3$, 400 MHz) δ 5.42 (dd, 1H, *J* = 4.9, 2.6, H-12), 4.49 (d, 1H, *J* = 5.7, H-3), 3.64 (s, 3H, $-OCH_3$-28), 3.33 (d, 1H, *J* = 5.7, 3-OH), 2.92 (dd, 1H, *J* = 14.5, 3.8, H-18), 2.79 (quad., 1H, *J* = 20.2, 6.6, H-1), 2.44 (m, 1H, H-9), 2.10 (dt, 1H, *J* = 17.3, 10.2, 5.3, H_2-11a), 2.00 (m, 4H, H_2-16a, H_2-11b, H_2-6), 1.70 (m, 2H, H_2-19a, H_2-22a), 1.67 (m, 1H, H_2-16b), 1.55 (m, 2H, H_2-22b, H_2-15a), 1.40 (d, 2H, *J* = 6.3, H_2-7), 1.37 (d, 3H, *J* = 6.6, H_3-25), 1.34 (ovl, 1H, H_2-

21a), 1.25 (ovl, 1H, H_2-15b), 1.21 (ovl, 4H, H_3-24, H_2-21b,) 1.17 (ovl, 1H, H_2-19b), 1.11 (s, 3H, H_3-27), 0.93 (s, 3H, H_3-29), 0.91 (s, 3H, H_3-30), 0.79 (s, 6H, H_3-26, H_3-23). ^{13}C NMR ($CDCl_3$, 100 MHz) δ 212.5 (C-2), 178.2 (C-28), 143.5 (C-13), 135.8 (C-5), 130.2 (C-10), 122.7 (C-12), 76.5 (C-3), 51.5 (OCH_3), 47.4 (C-17), 47.3 (C-4), 45.9 (C-1), 45.2 (C-19), 42.5 (C-18), 40.8 (C-14, C-9), 37.6 (C-8), 34.0 (C-21), 33.1 (C-30), 32.2 (C-22), 30.7 (C-20), 28.6 (C-11), 28.5 (C-7), 28.0 (C-15), 24.6 (C-24, C-27), 23.5 (C-29), 23.3 (C-25), 23.2 (C-6), 22.8 (C-16), 20.1 (C-23), 15.8 (C-26). HRMS (ESI) m/z: $[M + Na]^+$ Calcd for $C_{31}H_{46}O_4Na$ 505.3289; Found 505.3285.

*3-hydroxy-1,3,4-trimethyl,11-oxo-1,3,5-trien-oleanoic acid methyl ester (**10**)*: brownish-yellow solid. $[\alpha]^{20}_D$ +184.3 (*c* 1.3 $CHCl_3$); m.p.: 181 °C; ^{1}H NMR ($CDCl_3$, 400 MHz) δ 5.91 (s, 1H, H-12), 4.64 (s, 1H, 3-OH), 4.26 (s, 1H, H-9), 3.64 (s, 3H, -OCH_3-28), 3.10 (dd, 1H, J = 14.2, 3.6, H-18), 2.58 (m, 2H, H_2-6), 2.18 (s, 3H, H_3-24), 2.13 (s, 3H, H_3-23), 2.09 (ovl, 1H, H-16a), 1.89 (s, 3H, H_3-25), 1.81 (m, 1H, H-16b), 1.78 (m, 1H, H-22a), 1.76 (m, 1H, H-21a), 1.73 (m, 1H, H-7a), 1.69 (ovl, 1H, H-21b), 1.66 (ovl, 1H, H-7b), 1.62 (ovl, 1H, H-15a), 1.49 (ovl, 4H, H_3-27, H-15b), 1.41 (m, 2H, H-22b, H-19a), 1.31 (m, 1H, H-19b), 0.97 (s, 6H, H_3-29, H_3-30), 0.80 (s, 3H, H_3-26). ^{13}C NMR ($CDCl_3$, 100 MHz) δ 197.4 (C-11), 177.5 (C-28), 166.1 (C-13), 150.2 (C-2), 132.9 (C-4), 128.4 (C-10), 128.1 (C-12), 127.1 (C-5), 121.2 (C-1), 120.9 (C-3), 51.9 (C-9), 51.6 (OCH_3), 47.3 (C-17), 44.1 (C-14), 43.8 (C-8), 43.6 (C-21), 43.1 (C-18), 33.9 (C-19), 32.9 (C-30), 31.6 (C-22), 30.7 (C-20), 28.4 (C-7), 28.1 (C-15), 24.4 (C-6), 23.3 (C-16, C-29), 21.3 (C-27), 16.8 (C-26), 15.2 (C-23), 14.6 (C-25), 12.2 (C-24). HRMS (ESI) m/z: $[M-H]^-$ Calcd for $C_{31}H_{41}O_4$ 477.3010; Found 477.2995.

*1-methyl-2,5-dien-olean-29-oic acid methyl ester (**5a**)*: yellow solid. $[\alpha]^{20}_D$ +33.5 (*c* 0.5 $CHCl_3$); m.p.: 128 °C; ^{1}H NMR ($CDCl_3$, 400 MHz) δ 5.73 (dd, 1H, J = 9.5, 5.1, H-2), 5.49 (d, 1H, J = 9.5, H-3), 5.43 (m, 1H, H-12), 3.69 (s, 3H, -OCH_3-30), 2.61 (quint., 1H, J = 5.1, 25.2, H-1), 2.55 (m, 1H, H-9), 2.18 (ovl, 2H, H_2-11), 2.11 (ovl, 1H, H_2-6), 2.08 (ovl, 2H, H_2-16a), 1.90 (ovl, 1H, H_2-19a), 1.81 (ovl, 2H, H_2-15), 1.66 (ovl, 1H, H_2-19b), 1.63 (ovl, 2H, H_2-7a, H_2-21a), 1.32(ovl, 4H, H_2-7b, H_2-21b, H_2-22), 1.14 (s, 3H, H_3-27), 1.13 (s, 3H, H_3-29), 1.12 (ovl, 3H, H_3-25), 1.09 (ovl, 6H, H_3-23, H_3-24), 0.93 (s, 3H, H_3-26), 0.91 (ovl, 1H, H_2-16b), 0.81 (s, 3H, H_3-28). ^{13}C NMR ($CDCl_3$, 100 MHz) δ 177.7 (C-30), 143.9 (C-13), 136.1 (C-3), 135.6 (C-5), 133.1 (C-10), 131.2 (C-2), 123.4 (C-12), 51.5 (OCH_3), 49.4 (C-18), 44.5 (C-20), 42.2 (C-19), 41.0 (C-14), 39.5 (C-9), 38.2 (C-8, C-22),

36.4 (C-4), 33.4 (C-1), 32.4 (C-17), 31.3 (C-21), 30.1 (C-24), 30.7 (C-20), 30.1 (C-24), 29.3 (C-23), 29.1 (C-7), 28.6 (C-29), 28.3 (C-11), 27.1 (C-16), 26.3 (C-15), 24.3 (C-25), 21.4 (C-6), 16.6 (C-26). HRMS (ESI) m/z: $[M+Na]^+$ Calcd for $C_{31}H_{46}O_2Na$ 473.3390; Found 473.3395.

*(2α)-2-hydroxy-1-methyl-3-oxo-5-dien-olean-29-oic acid methyl ester (**6a**)*: yellow solid. $[\alpha]^{20}_D$ +175.3 (*c* 1.4 $CHCl_3$); m.p.: 173 °C; ^1H NMR ($CDCl_3$, 400 MHz) δ 5.42 (dd, 1H, J = 4.9, 2.6, H-12), 3.77 (ovl, 1H, H-2), 3.69 (s, 3H, -OCH_3-30), 2.56 (ovl, 1H, H-1), 2.53 (m, 1H, H-9), 2.24 (m, 1H, H_2-11a), 2.20 (m, 2H, H_2-6a, H_2-16a), 2.00 (m, 1H, H_2-6b), 1.96 (ovl, 3H, H_2-16b, H-18, H_2-21a), 1.93 (ovl, 2H, H_2-11b, H_2-19a), 1.82 (m, 1H, H_2-15a), 1.69 (m, 1H, H_2-19b), 1.60 (ovl, 1H, H_2-7a), 1.52 (ovl, 1H, H_2-7b), 1.33 (ovl, 2H, H_2-22), 1.32 (ovl, 1H, H_2-21b), 1.31 (s, 3H, H_3-23), 1.22 (ovl, 1H, H_2-15b), 1.9 (s, 3H, H_3-24), 1.17 (s, 3H, H_3-27), 1.14 (s, 3H, H_3-29), 0.91 (d, 3H, J = 7.1, H_3-25), 0.90 (s, 3H, H_3-26), 0.81 (s, 3H, H_3-28). ^{13}C NMR ($CDCl_3$, 100 MHz) δ 214.1 (C-3), 177.6 (C-30), 144.4 (C-13), 134.9 (C-5), 130.9 (C-10), 122.9 (C-12), 78.5 (C-2), 51.6 (OCH_3), 49.4 (C-18), 46.0 (C-4), 44.3 (C-20), 42.2 (C-19), 40.9 (C-14), 40.6 (C-9), 39.8 (C-1), 38.2 (C-22), 37.8 (C-8), 32.4 (C-17), 31.3 (C-21), 28.6 (C-29), 28.3 (C-28), 28.1 (C-7), 27.2 (C-23), 27.0 (C-11, C-16), 26.2 (C-15), 24.7 (C-24, C-27), 20.8 (C-6), 18.3 (C-25), 15.3 (C-26). HRMS (ESI) m/z: $[M+Na]^+$ Calcd for $C_{31}H_{46}O_4Na$ 505.3288; Found 505.3296.

*(3α)-3-hydroxy-1-methyl-2-oxo-5-dien-olean-29-oic acid methyl ester (**7a**)*: white solid. $[\alpha]^{20}_D$ +166.3 (*c* 6 $CHCl_3$); m.p.: 164 °C; ^1H NMR ($CDCl_3$, 400 MHz) δ 5.39 (dd, 1H, J = 4.9, 2.6, H-12), 4.51 (d, 1H, J = 5.7, H-3), 3.69(s, 3H, -OCH_3-30), 3.35 (d, 1H, J = 5.7, 3-OH), 2.81 (quad., 1H, J = 20.2, 6.6, H-1), 2.46 (m, 1H, H-9), 2.15 (dt, 1H, J = 17.3, 10.2, 5.3, H-11a), 2.04 (m, 2H, H_2-6), 2.00 (ovl, 1H, H-11b), 1.97 (ovl, 1H, H-16a), 1.94 (ovl, 1H, H-21a), 1.91 (m, 2H, H-19a, H-18), 1.78 (m, 1H, H-15a), 1.67 (m, 1H, H-19b), 1.45 (m, 2H, H_2-7), 1.39 (d, 3H, J = 6.6, H_3-25), 1.32 (ovl, 3H, H_2-22, H-21b), 1.23 (s, 3H, H_3-24), 1.17 (ovl, 1H, H-15b), 1.13 (s, 3H, H_3-29), 1.11 (s, 3H, H_3-27), 0.98 (s, 3H, H_3-26), 0.92 (ovl, 1H, H-16b) 0.81 (s, 6H, H_3-28, H_3-23). ^{13}C NMR ($CDCl_3$, 100 MHz) δ 212.5 (C-2), 177.6 (C-30), 144.2 (C-13), 135.8 (C-5), 130.3 (C-10), 122.8 (C-12), 76.5 (C-3), 51.5 (OCH_3), 49.3 (C-18), 47.4 (C-4), 46.0 (C-1), 44.2 (C-20), 42.1 (C-19), 40.9 (C-9), 40.8 (C-14), 38.2 (C-22), 37.9 (C-8), 32.4 (C-17), 31.3 (C-21), 28.6 (C-7), 28.6 (C-29), 28.5 (C-11), 28.3 (C-28), 27.0 (C-16), 26.2 (C-15), 24.6 (C-24, C-27), 23.3 (C-25), 22.9 (C-6), 20.1 (C-23), 15.9 (C-26). HRMS

(ESI) m/z: $[M+Na]^+$ Calcd for $C_{31}H_{46}O_4Na$ 505.3288; Found 505.3282.

*3-hydroxy-1,3,4-trimethyl,11-oxo-1,3,5-trien-olean-29-oic acid methyl ester (**10a**)*: brownish yellow solid. $[\alpha]^{20}_D$ +133.2 (*c* 5 $CHCl_3$); m.p.: 192 °C; ^1H NMR ($CDCl_3$, 400 MHz) δ 5.93 (s, 1H, H-12), 4.61 (s, 1H, 3-OH), 4.28 (s, 1H, H-9), 3.70 (s, 3H, $-OCH_3$-30), 2.61 (m, 2H, H_2-6), 2.18 (ovl, 4H, H_3-24, H_2-18), 2.15 (s, 3H, H_3-23), 2.10 (m, 1H, H_2-15a), 2.04 (m, 1H, H_2-19a), 2.02 (m, 1H, H_2-21a), 1.94 (m, 1H, H_2-15b), 1.91 (s, 3H, H_3-25), 1.79 (m, 1H, H_2-19b), 1.76 (m, 1H, H_2-7a), 1.69 (ovl, 1H, H_2-7b), 1.49 (s, 3H, H_3-27), 1.44 (m, 2H, H_2-16), 1.43 (m, 1H, H_2-22a), 1.37 (ovl, 2H, H_2-22b, H_2-21b), 1.19 (s, 3H, H_3-29), 0.98 (s, 3H, H_3-26), 0.85 (s, 3H, H_3-28). ^{13}C NMR ($CDCl_3$, 100 MHz) δ 197.8 (C-11), 176.9 (C-30), 166.8 (C-13), 150.2 (C-2), 133.0 (C-4), 128.4 (C-10), 128.1 (C-12), 127.1 (C-5), 121.1 (C-1), 120.9 (C-3), 51.8 (C-9), 51.7(OCH_3), 49.9 (C-18), 44.1 (C-14, C-20), 43.9 (C-8), 40.5 (C-19), 37.7 (C-22), 32.9 (C-17), 31.2 (C-21), 28.7 (C-28), 28.5 (C-7), 28.4 (C-29), 26.7 (C-16), 26.6 (C-15), 24.4 (C-6), 21.2 (C-27), 17.0 (C-26), 15.2 (C-23), 14.6 (C-25), 12.2 (C-24). HRMS (ESI) m/z: $[M-H]^-$ Calcd for $C_{31}H_{41}O_4$ 477.3010; Found 477.3013.

(1) Kratena, N.; Kaiser, M.; Naumov, K.; Waxmann, M.; Gaertner, P. Bioinspired Synthesis of Alstoscholarinoids A and B. *JACS Au* **2025**. https://doi.org/10.1021/jacsau.5c00102.

(2) Elkin, M.; Scruse, A. C.; Turlik, A.; Newhouse, T. R. Computational and Synthetic Investigation of Cationic Rearrangement in the Putative Biosynthesis of Justicane Triterpenoids. *Angew. Chemie - Int. Ed.* **2019**, *58* (4), 1025–1029. https://doi.org/10.1002/anie.201810566.

(3) JIN, Bohan; DONG, Qing; HUNG, Gene; KALDOR, S. WO_2020068689_A1.Pdf.

(4) Holt, E.; Wang, M.; Harry, S. A.; He, C.; Wang, Y.; Henriquez, N.; Xiang, M. R.; Zhu, A.; Ghorbani, F.; Lectka, T. An Electrochemical Approach to Directed Fluorination. *J. Org. Chem.* **2023**, *88* (4), 2557–2560. https://doi.org/10.1021/acs.joc.2c01886.

(5) Siewert, B.; Wiemann, J.; Köwitsch, A.; Csuk, R. The Chemical and Biological Potential of C Ring Modified Triterpenoids. *Eur. J. Med. Chem.* **2014**, *72*, 84–101. https://doi.org/10.1016/j.ejmech.2013.11.025.

1.2. ^{1}H NMR spectrum of compound 2 (400 MHz, $CDCl_3$)

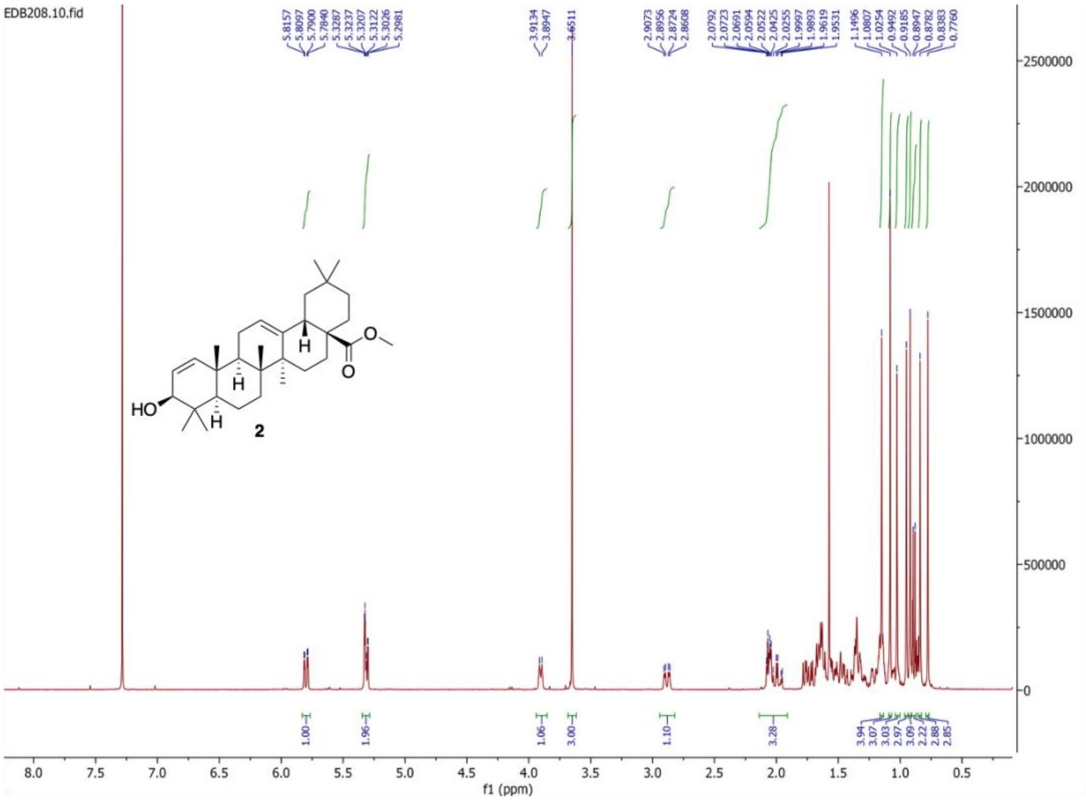

1.3. ^{13}C NMR spectrum of compound 2 (400 MHz, $CDCl_3$)

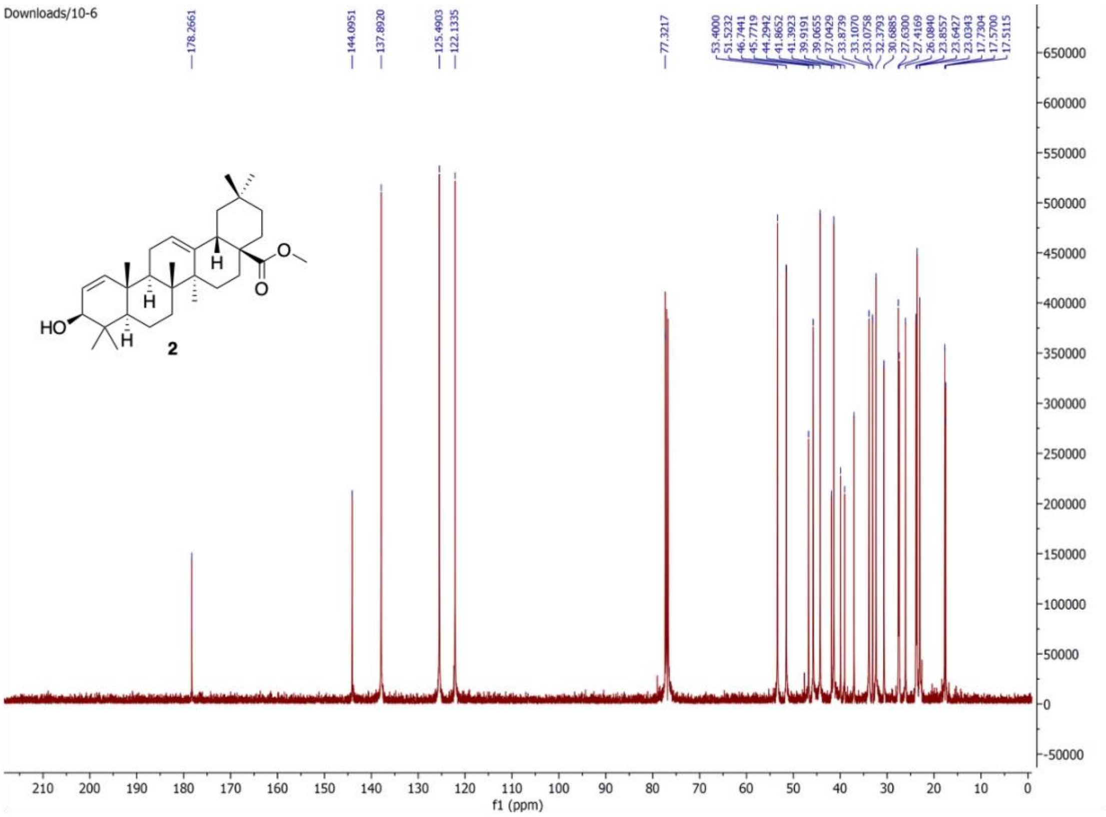

1.4. ^{1}H NMR spectrum of compound 8 (400 MHz, $CDCl_3$)

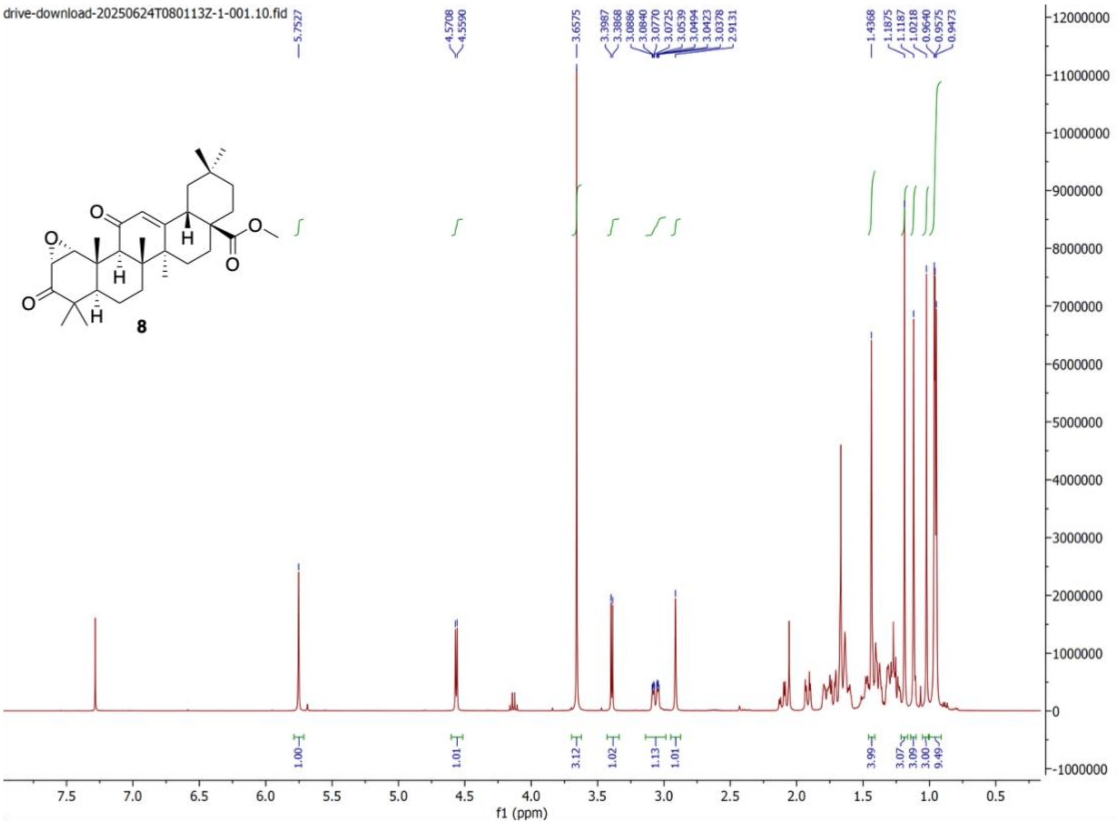

1.5. ^{13}C NMR spectrum of compound 8 (400 MHz, $CDCl_3$)

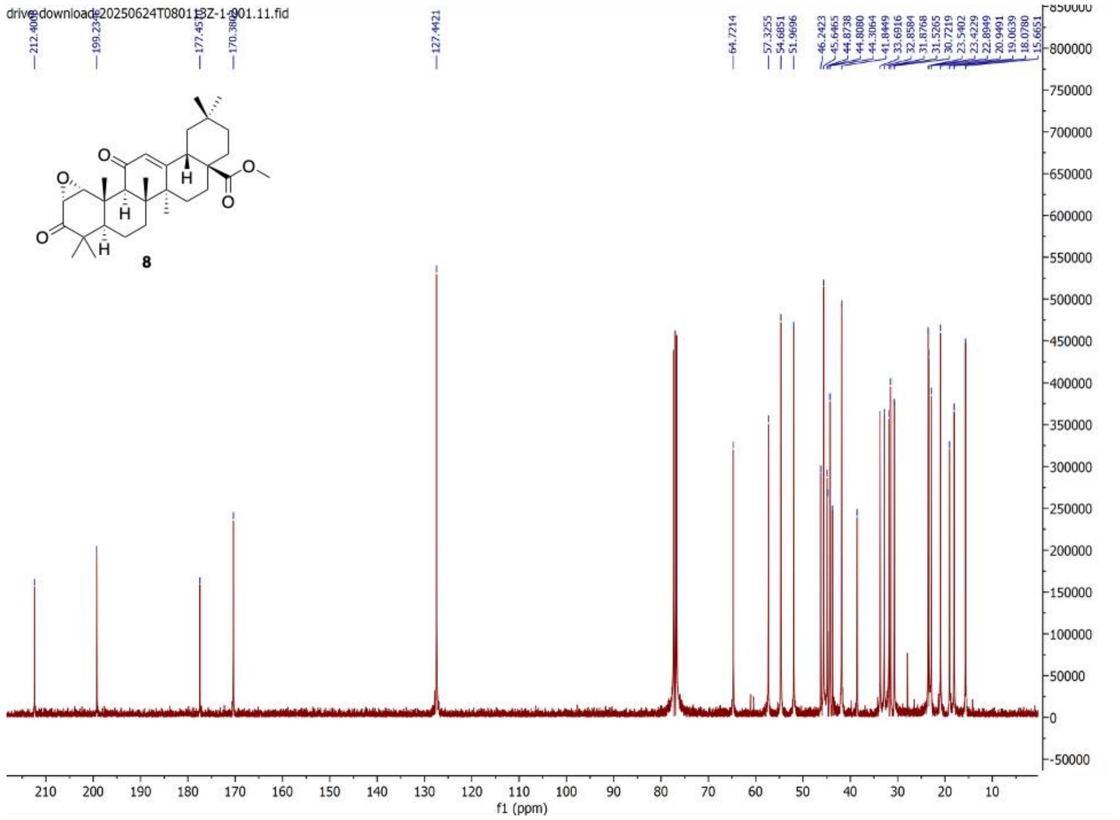

1.6. ^{1}H NMR spectrum of compound 2a (400 MHz, $CDCl_3$)

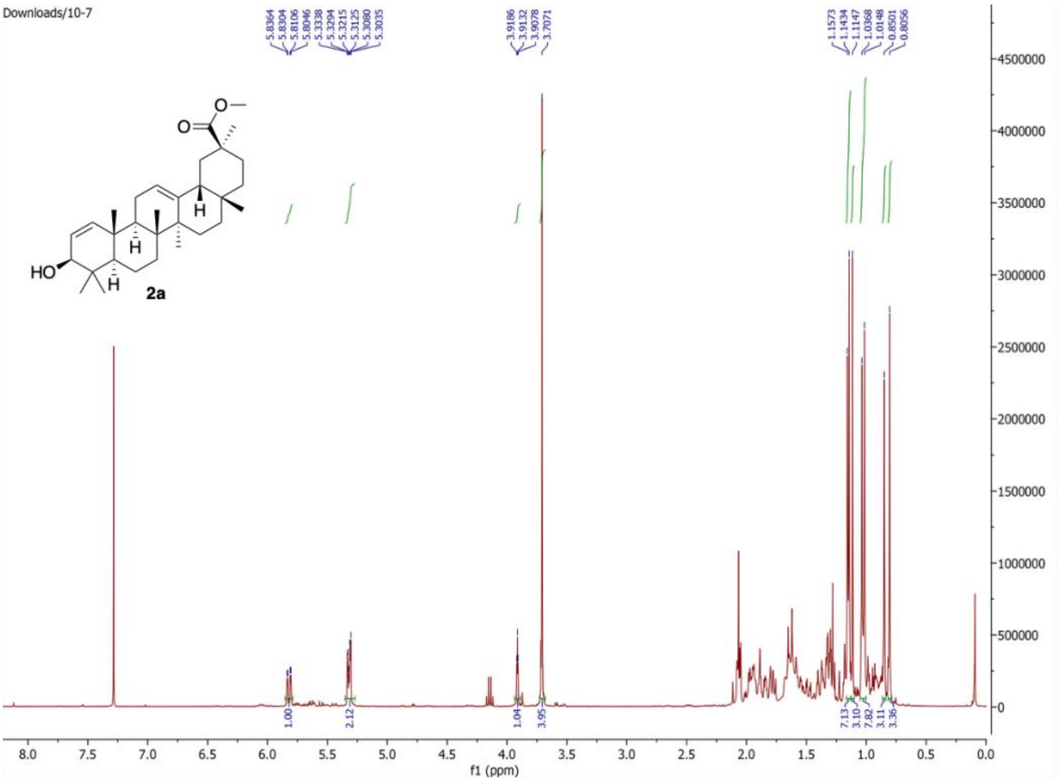

1.7. ^{13}C NMR spectrum of compound 2a (400 MHz, $CDCl_3$)

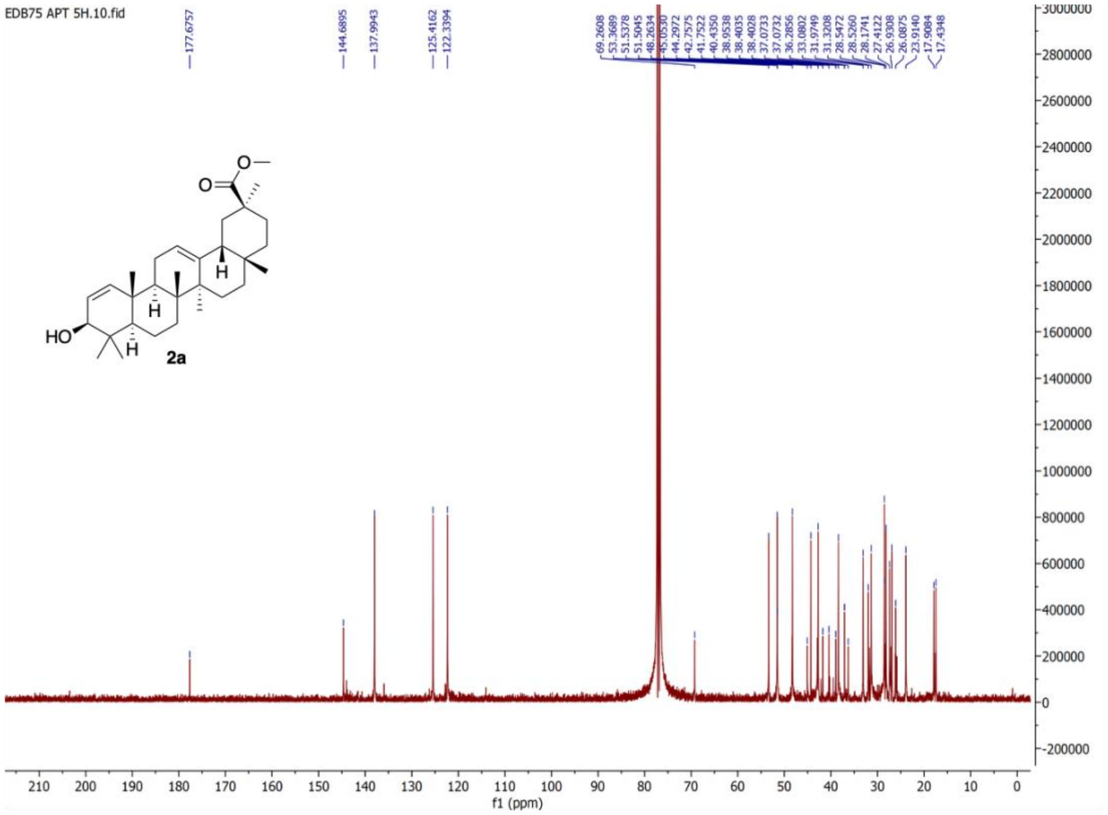

1.8. ^{1}H NMR spectrum of compound 3a (400 MHz, $CDCl_3$)

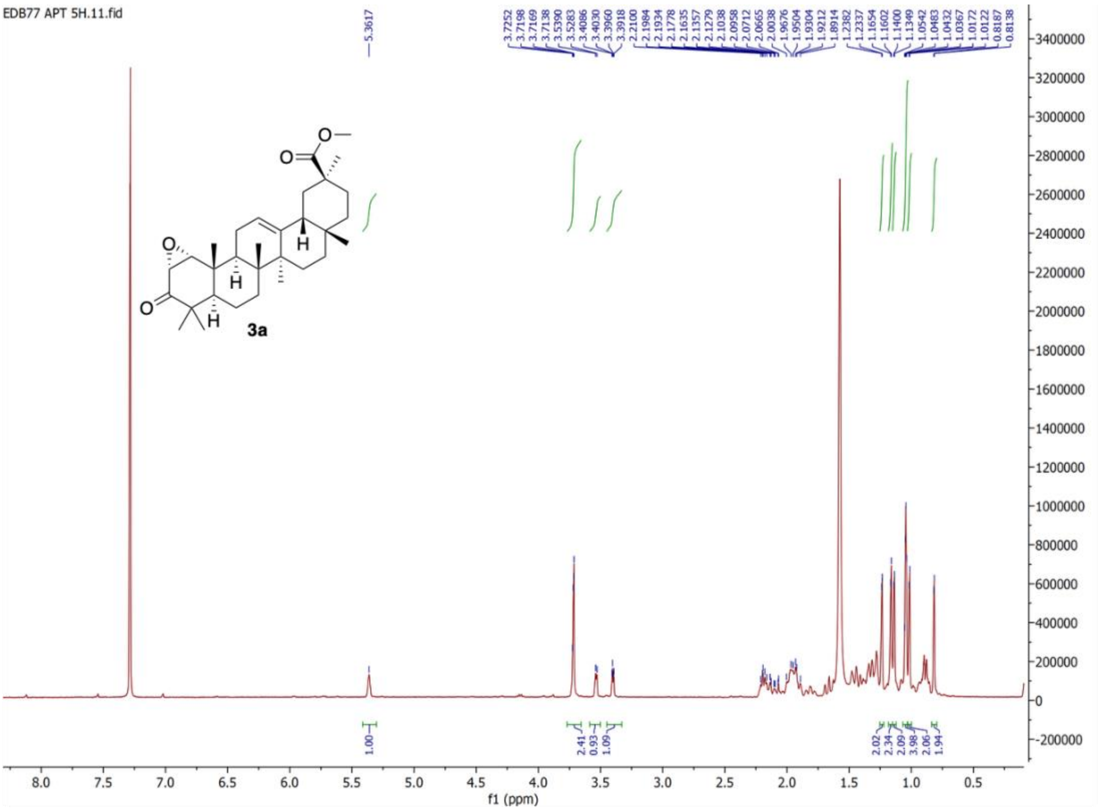

1.9. ^{13}C NMR spectrum of compound 3a (400 MHz, $CDCl_3$)

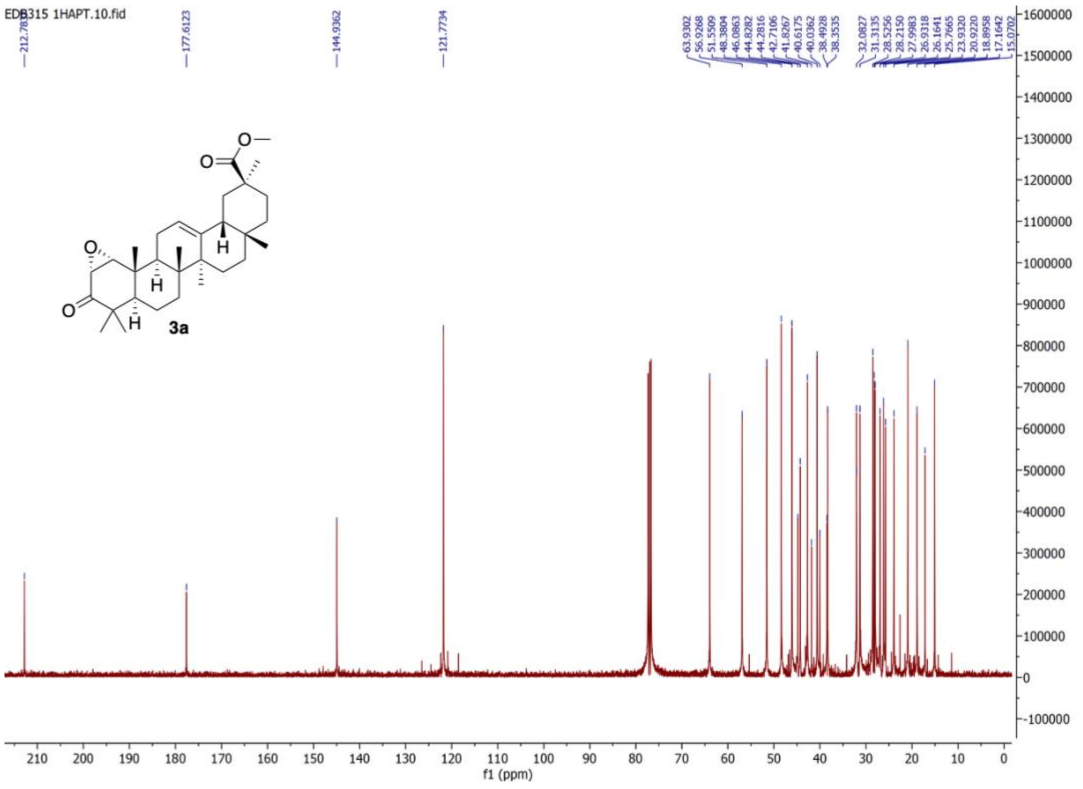

1.10. NMR spectrum of compound 8a (400 MHz, $CDCl_3$)

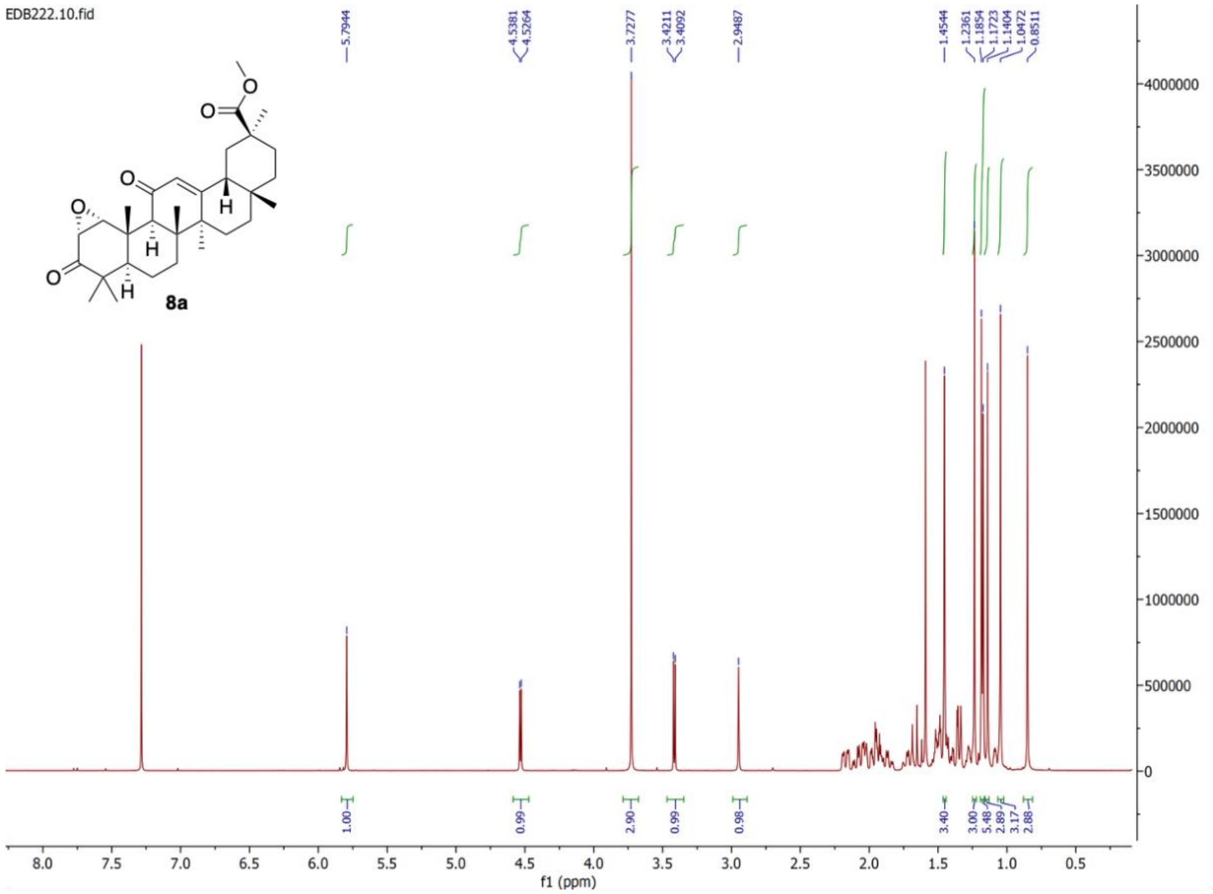

1.11. ^{13}C NMR spectrum of compound 8a (400 MHz, $CDCl_3$)

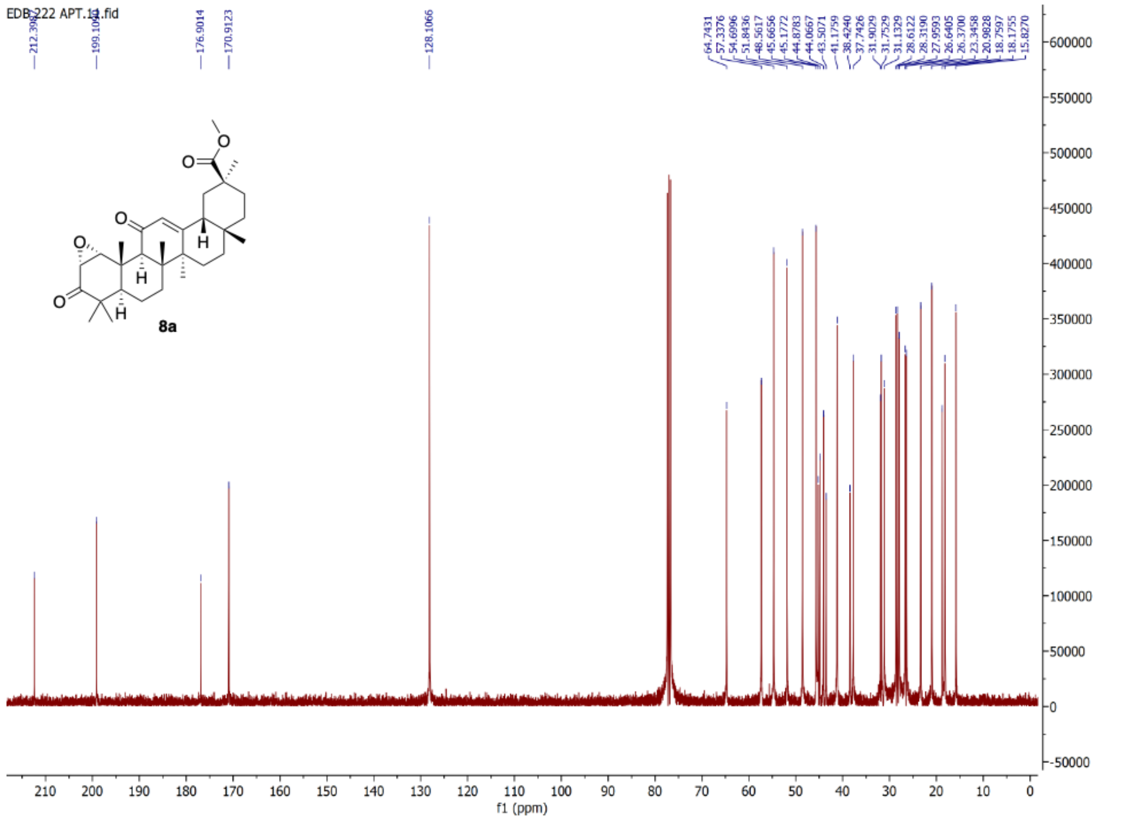

1.12.NMR spectrum of compound 5 (400 MHz, $CDCl_3$)

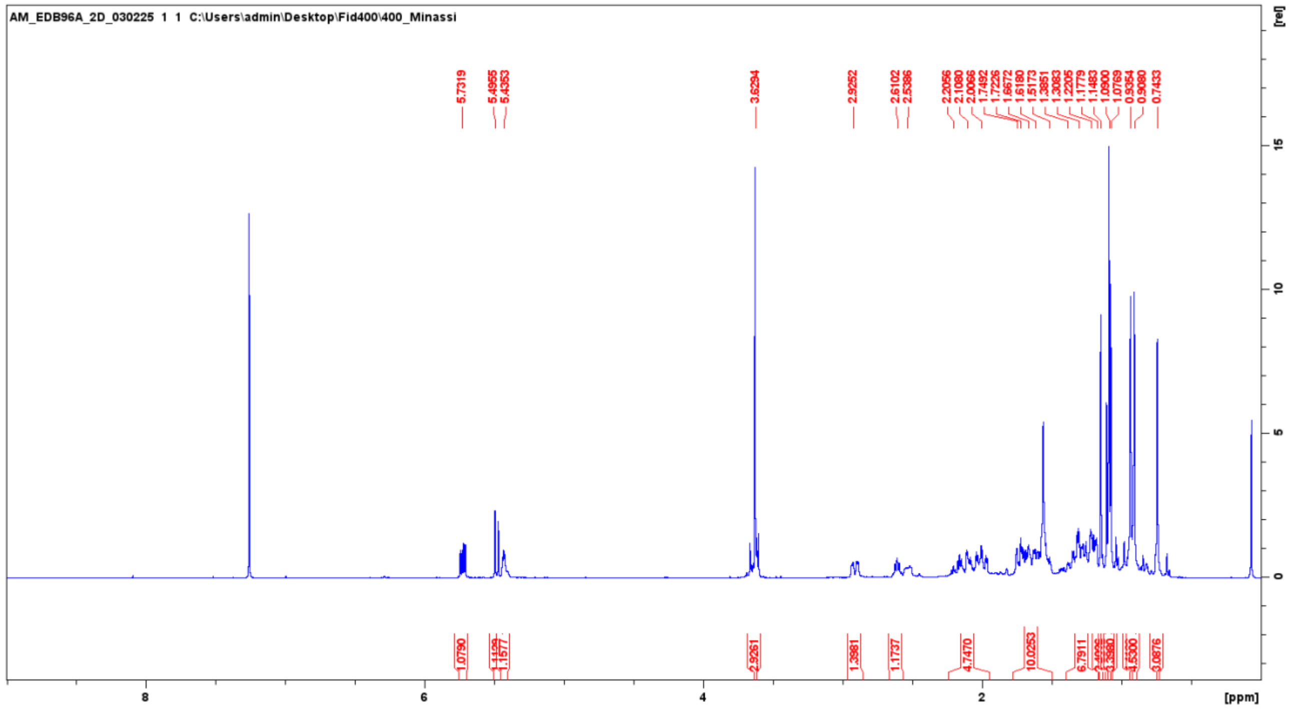

1.13.^{13}C NMR spectrum of compound 5 (400 MHz, $CDCl_3$)

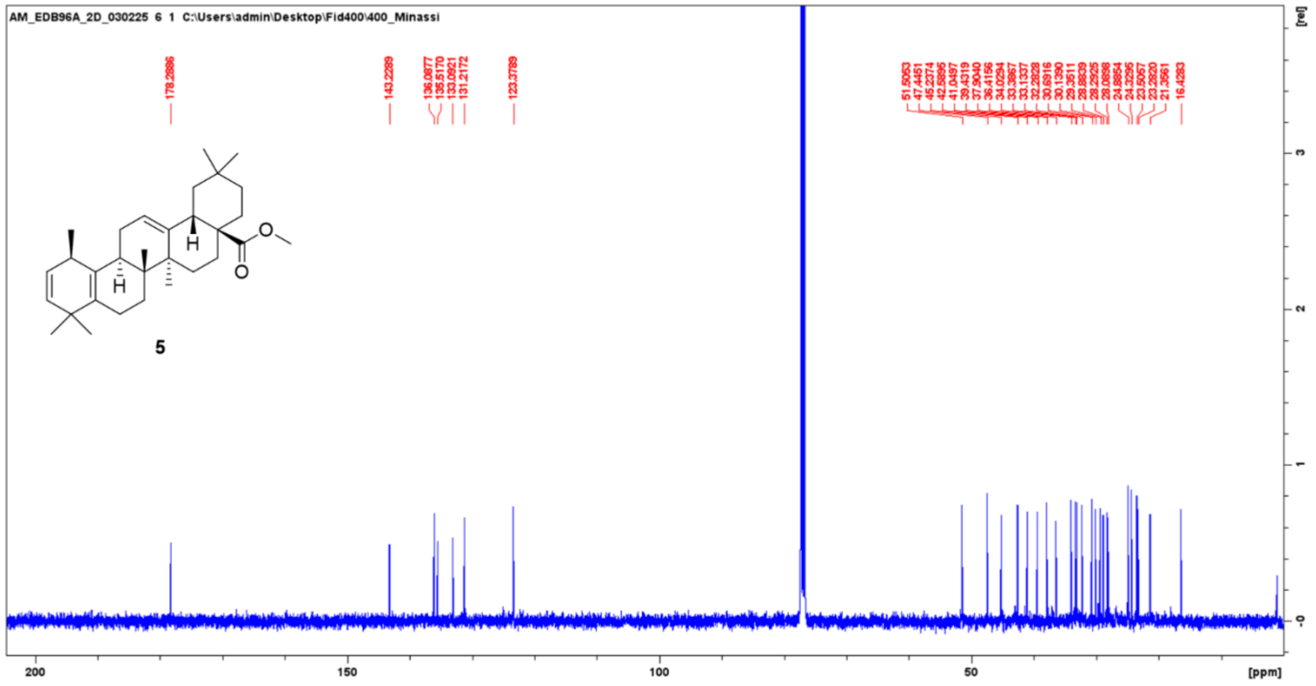

1.14. COSY NMR spectrum of compound 5 (400 MHz, $CDCl_3$)

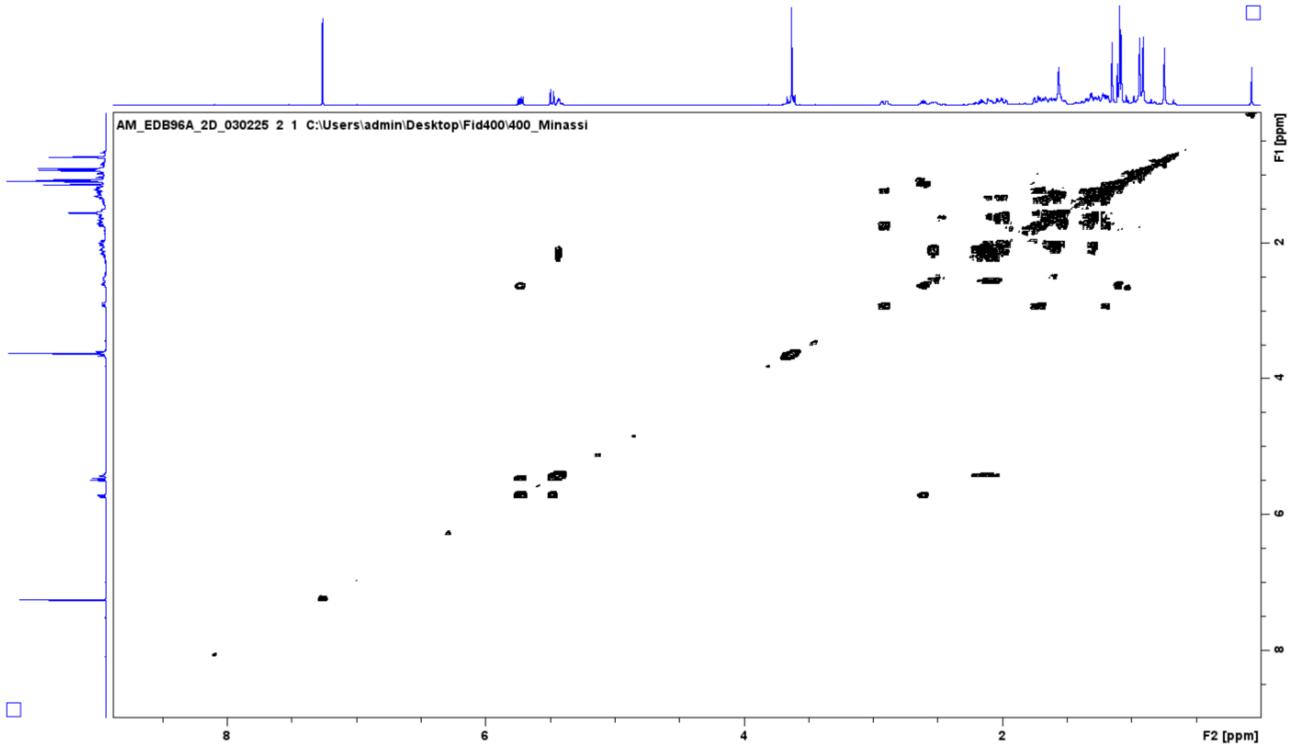

1.15.2D HSQC NMR spectrum of compound 5 (400 MHz, $CDCl_3$)

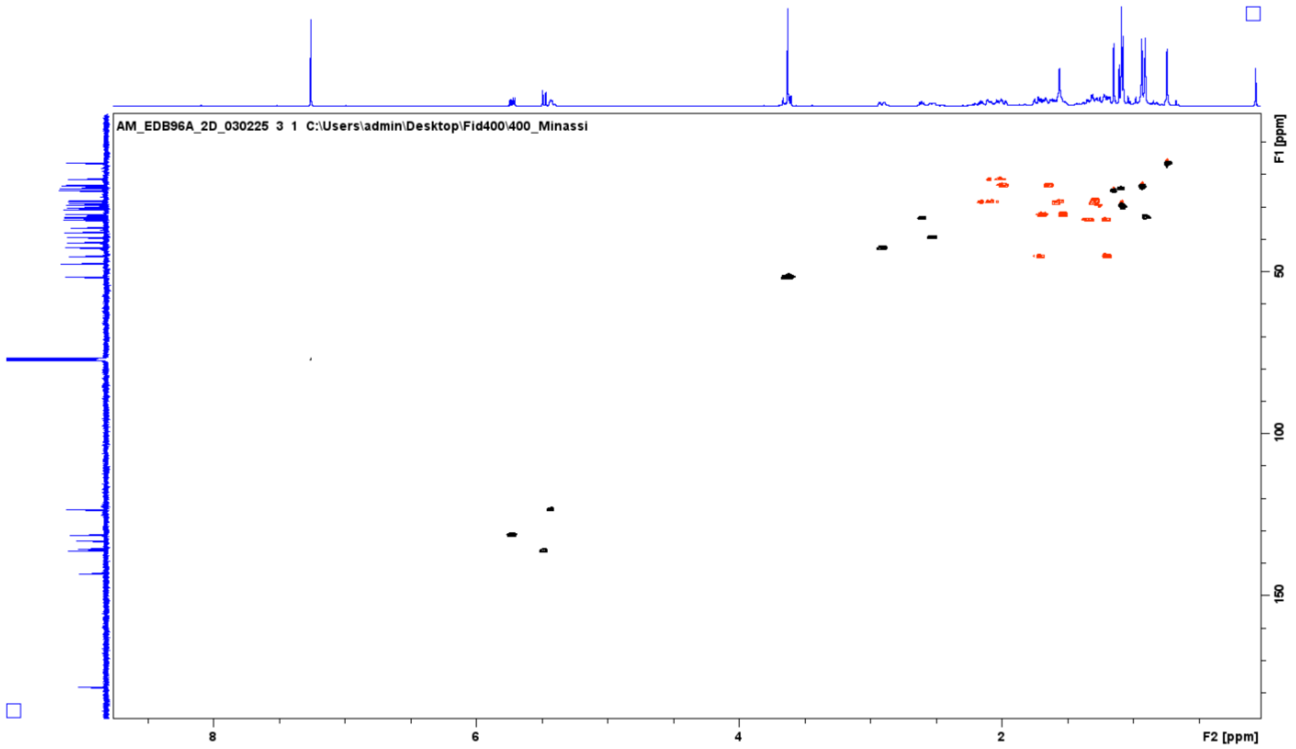

1.16.2D HMBC NMR spectrum of compound 5 (400 MHz, $CDCl_3$)

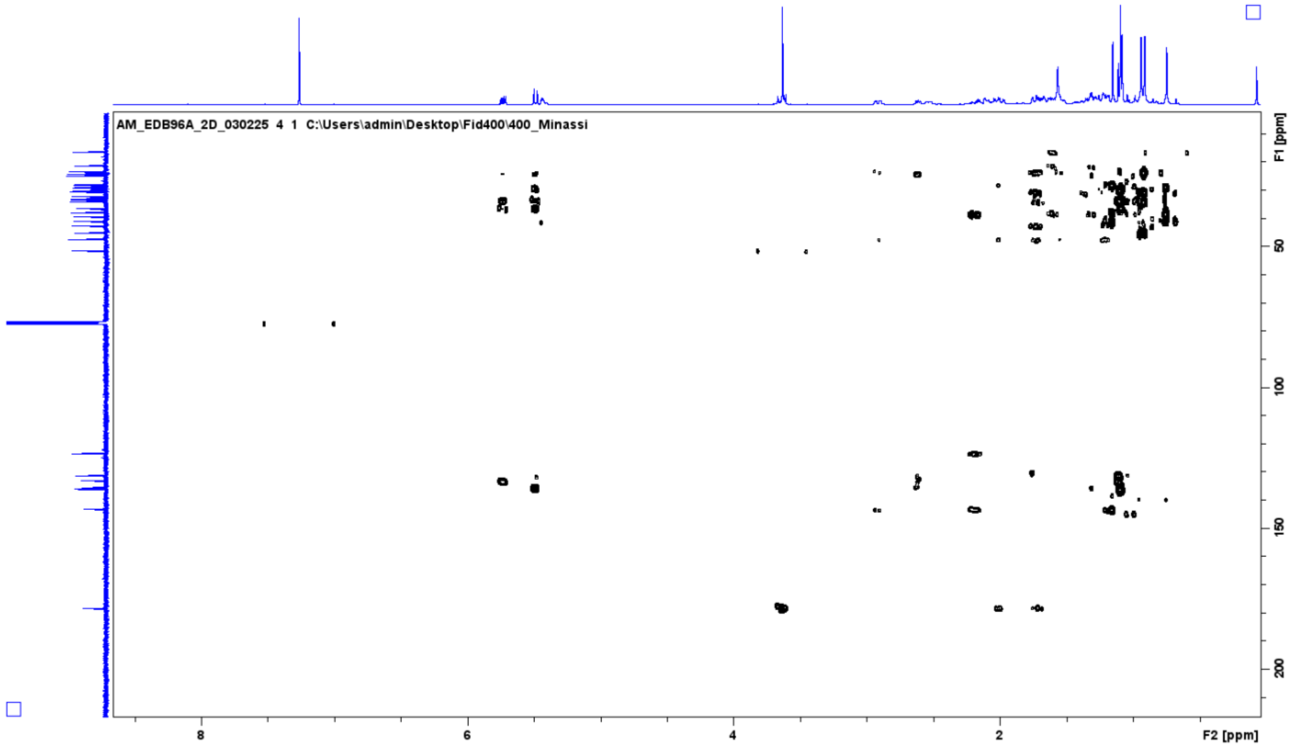

1.17.2D NOESY NMR spectrum of compound 5 (400 MHz, $CDCl_3$)

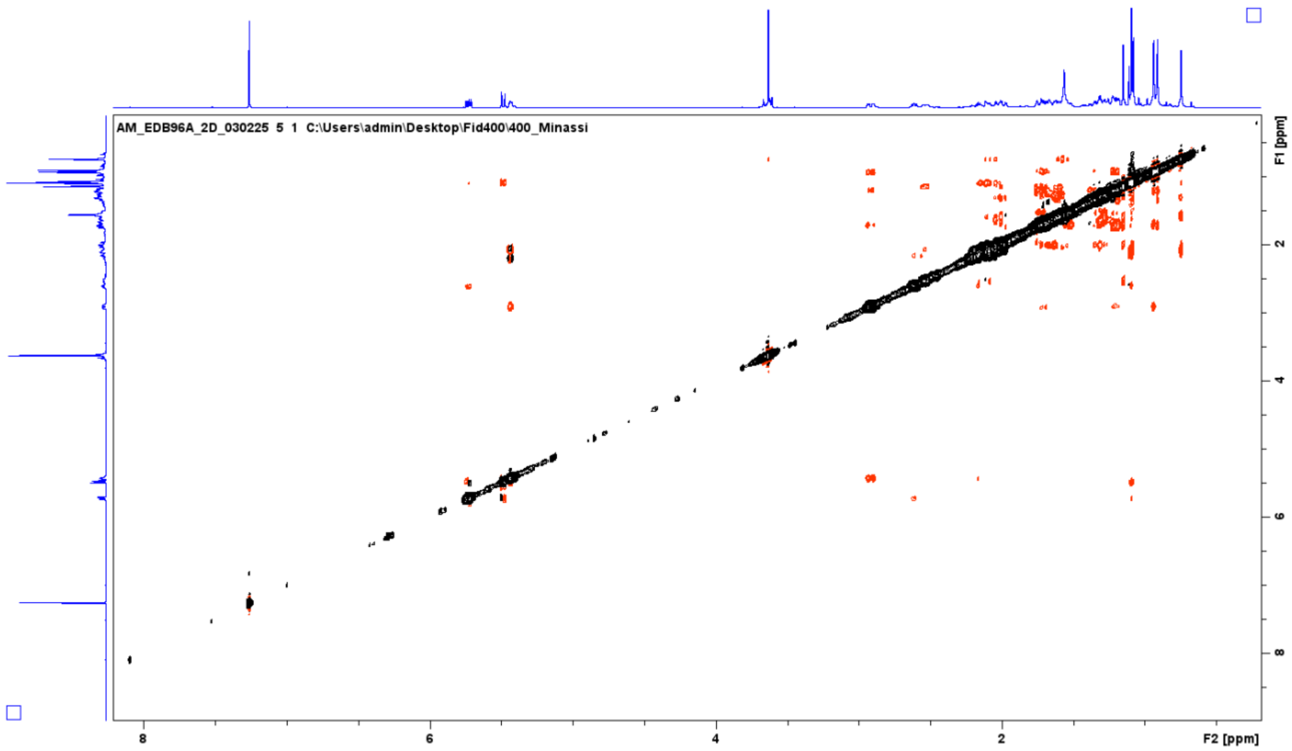

1.17 ^{1}H NMR spectrum of compound 6 (400 MHz, $CDCl_3$)

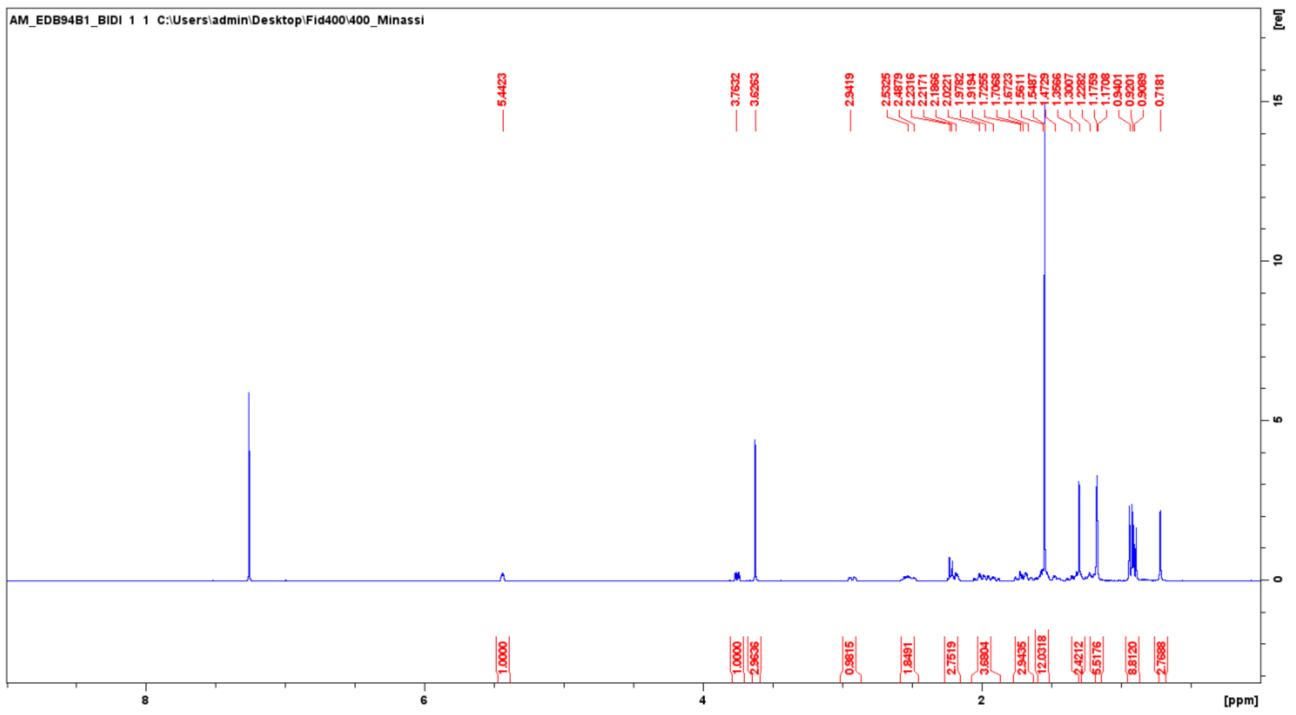

1.18 ^{13}C NMR spectrum of compound 6 (400 MHz, $CDCl_3$)

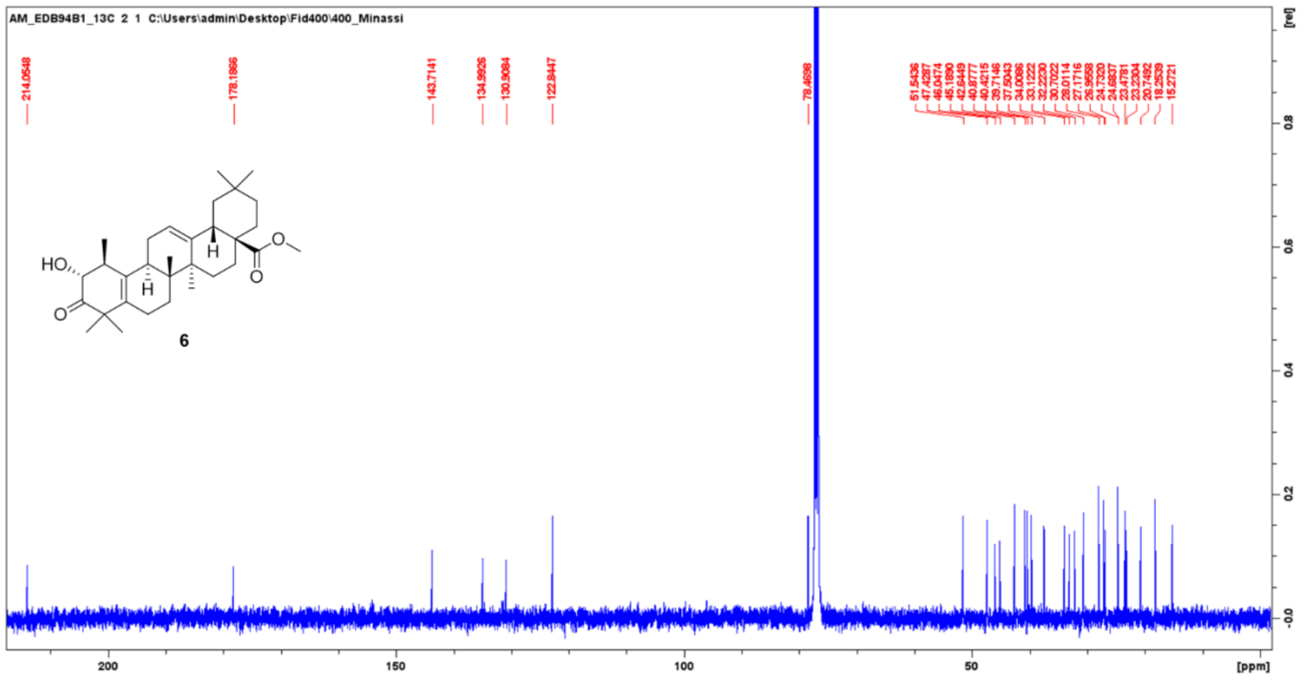

1.19 COSY NMR spectrum of compound 6 (400 MHz, $CDCl_3$)

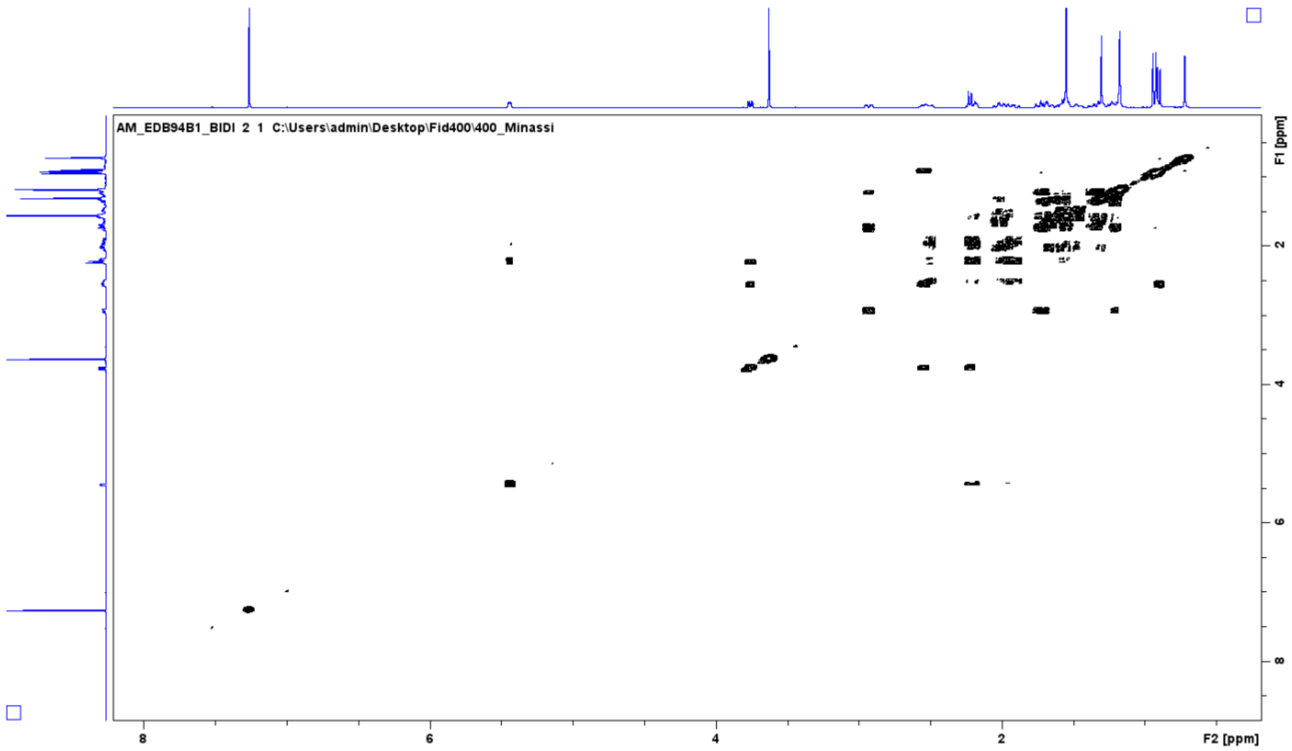

1.20. HSQC NMR spectrum of compound 6 (400 MHz, $CDCl_3$)

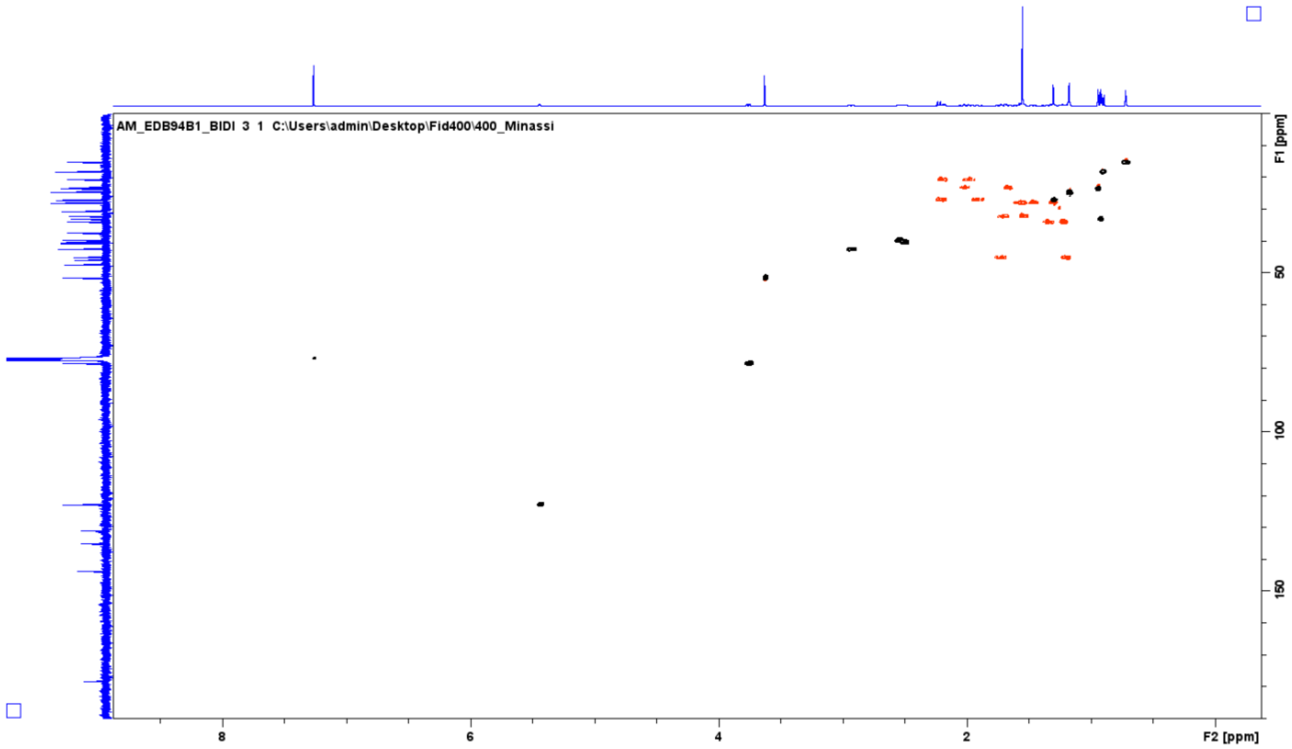

1.21. HMBC NMR spectrum of compound 6 (400 MHz, $CDCl_3$)

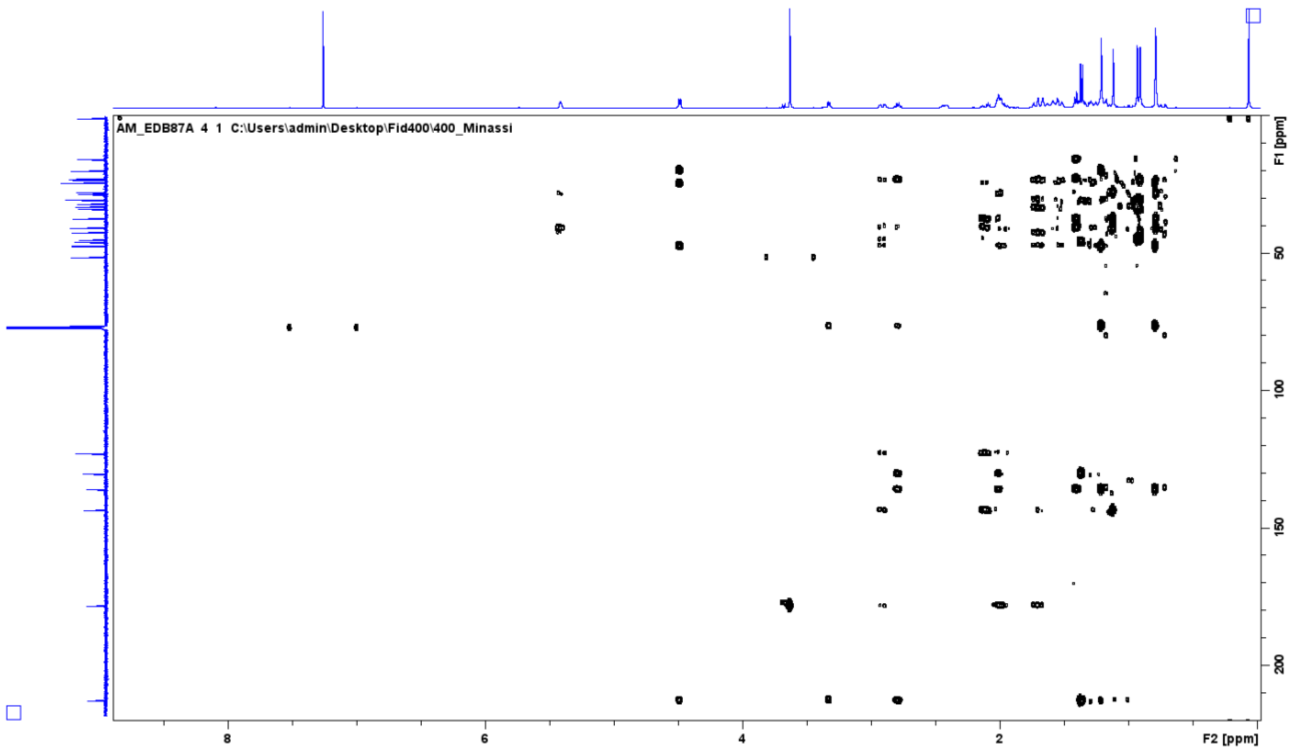

1.22. NOESY NMR spectrum of compound 6 (400 MHz, $CDCl_3$)

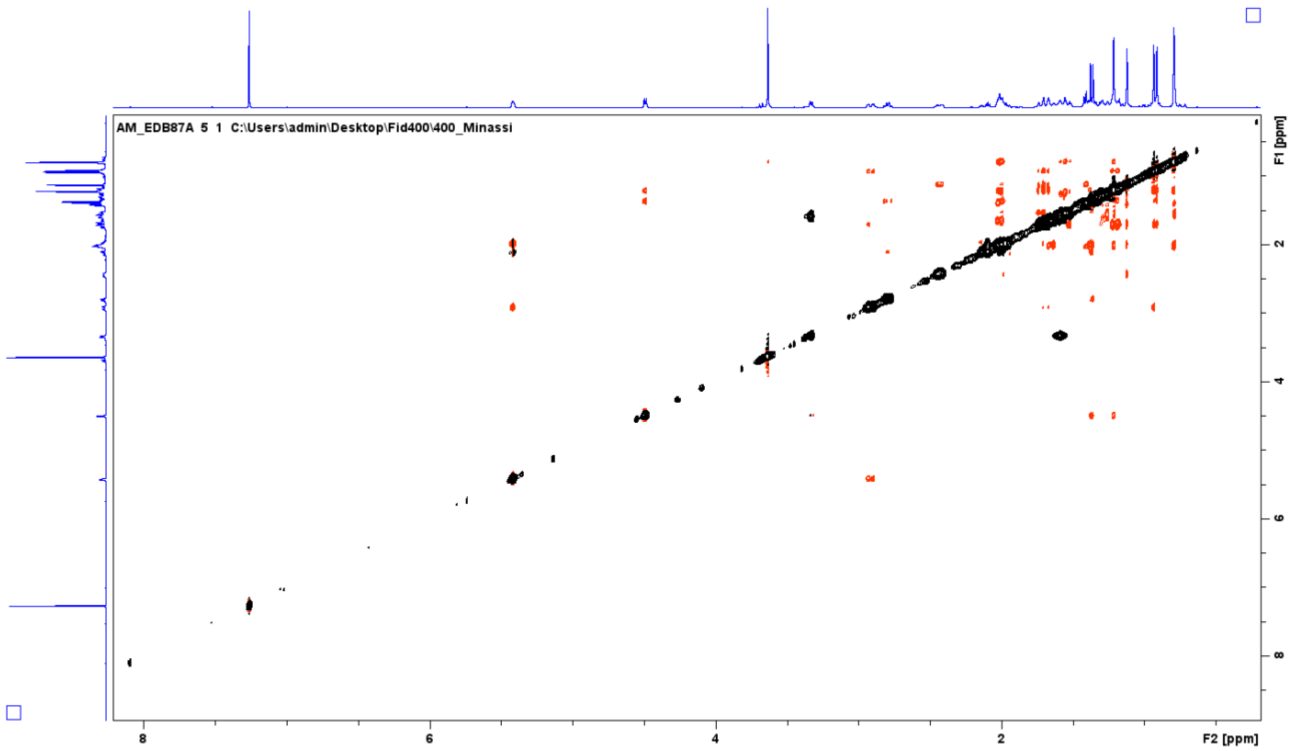

1.23 ^{1}H NMR spectrum of compound 7 (400 MHz, $CDCl_3$)

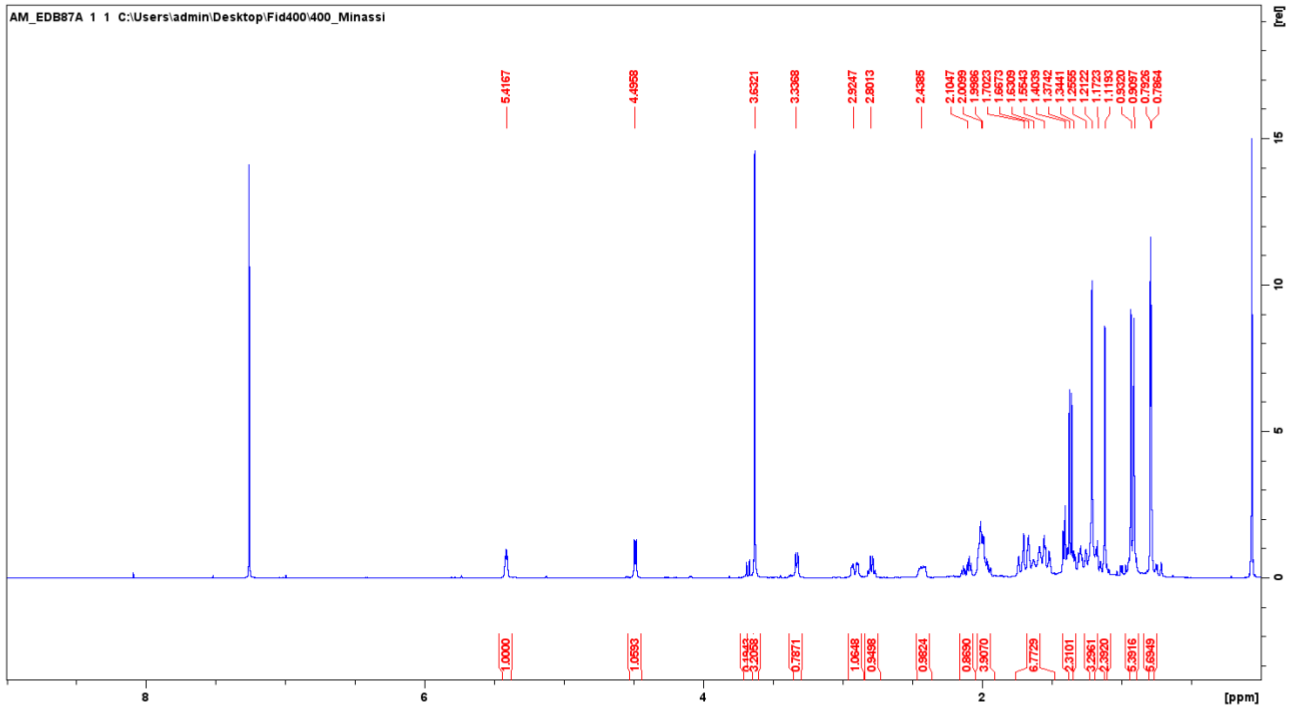

1.24 ^{13}C NMR spectrum of compound 7 (400 MHz, $CDCl_3$)

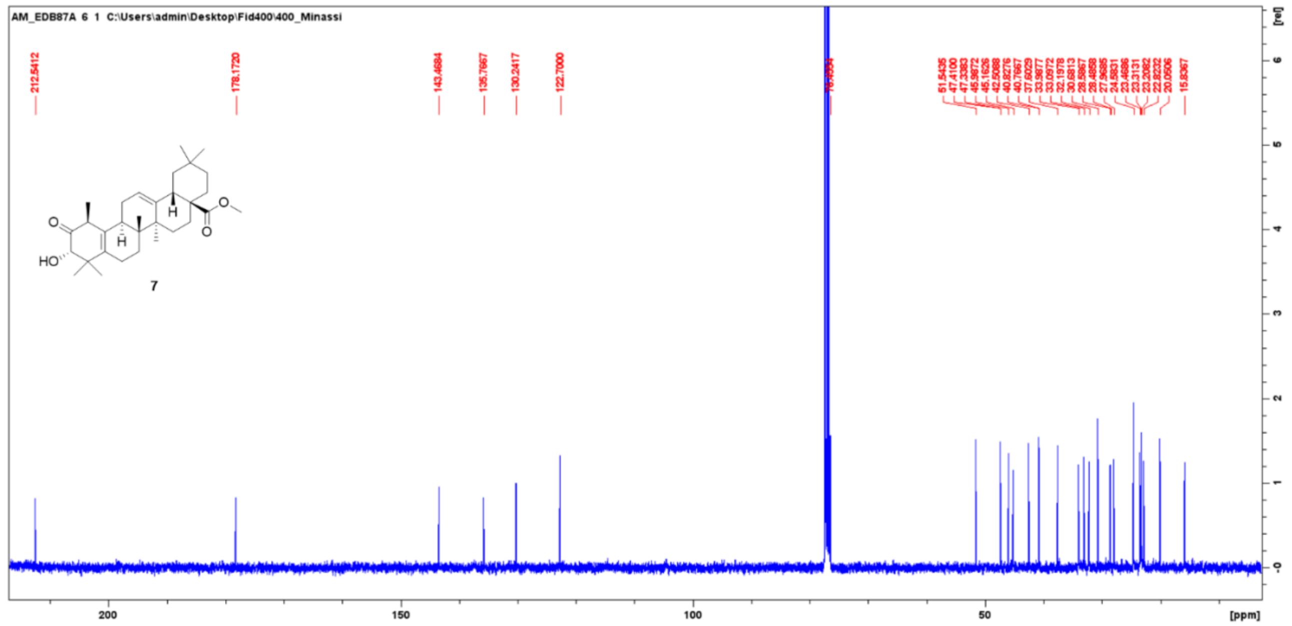

1.25 COSY NMR spectrum of compound 7 (400 MHz, $CDCl_3$)

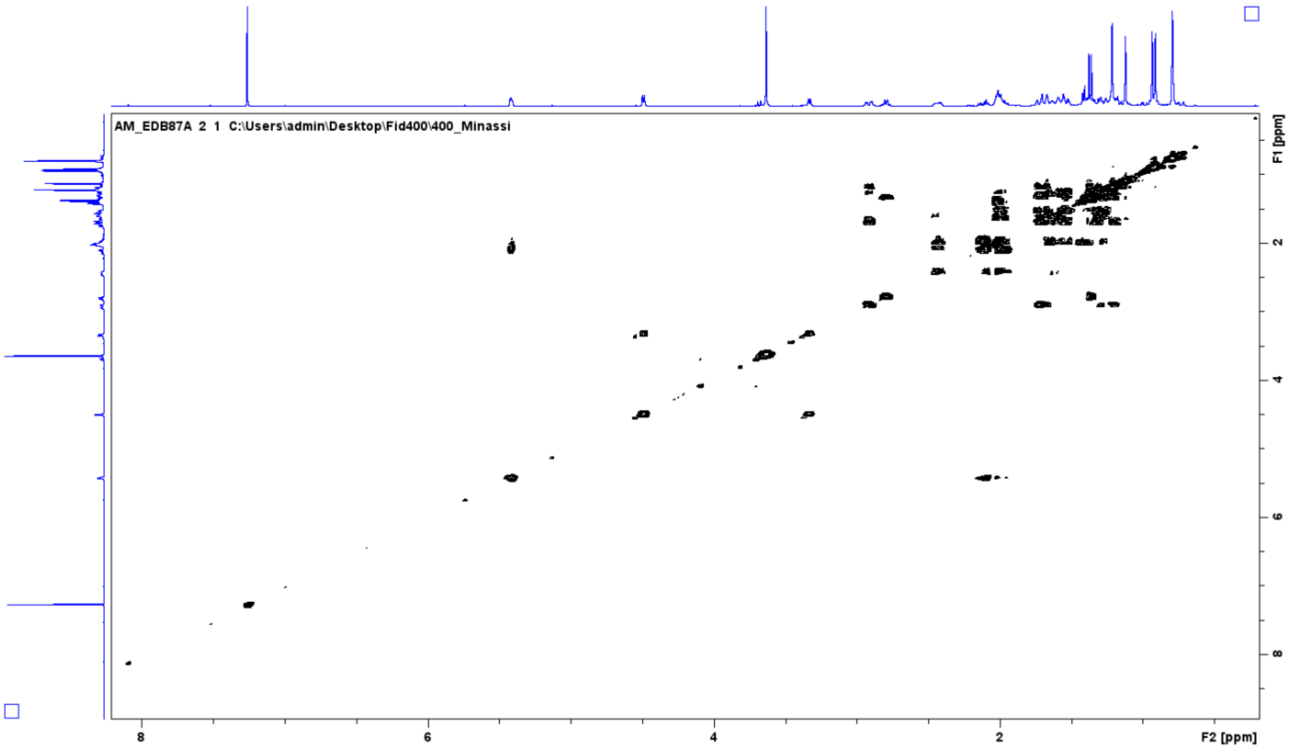

1.26 HSQC NMR spectrum of compound 7 (400 MHz, $CDCl_3$)

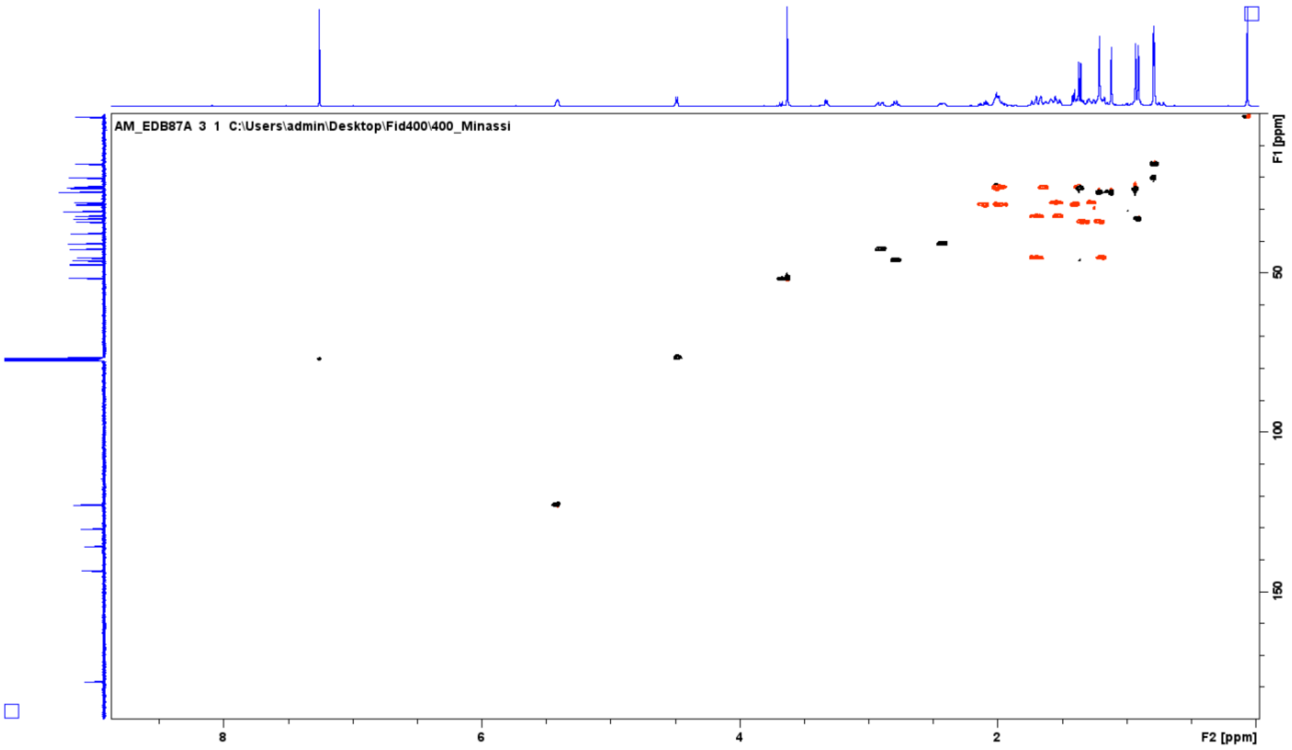

1.27. HMBC NMR spectrum of compound 7 (400 MHz, $CDCl_3$)

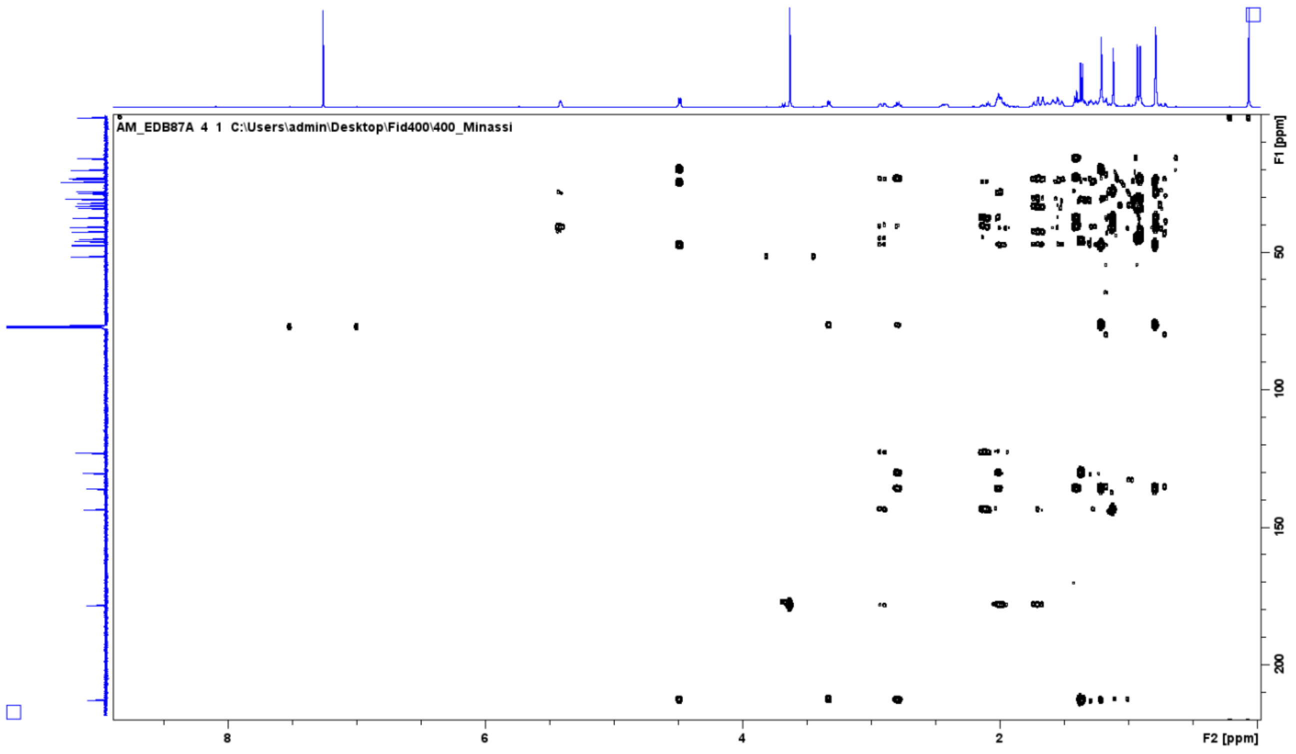

1.28 NOESY NMR spectrum of compound 7 (400 MHz, $CDCl_3$)

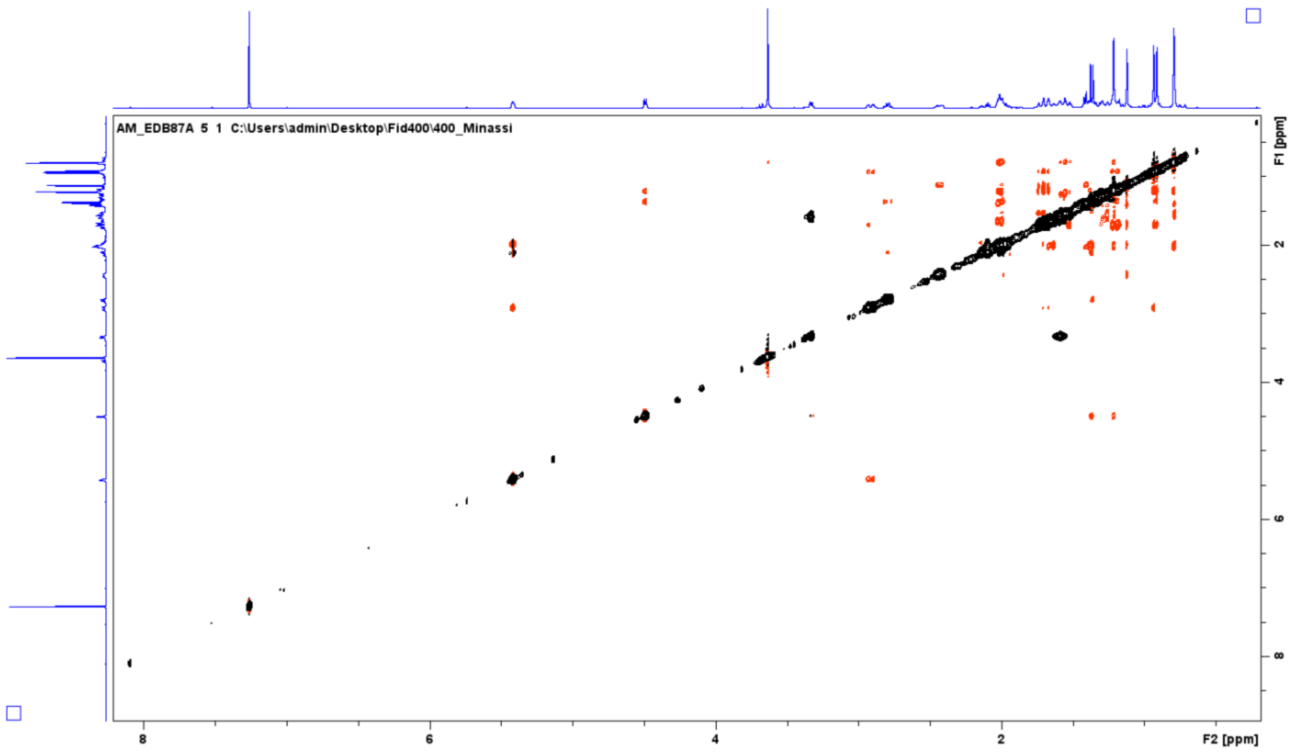

1.29 ^{1}H NMR spectrum of compound 10 (400 MHz, $CDCl_3$)

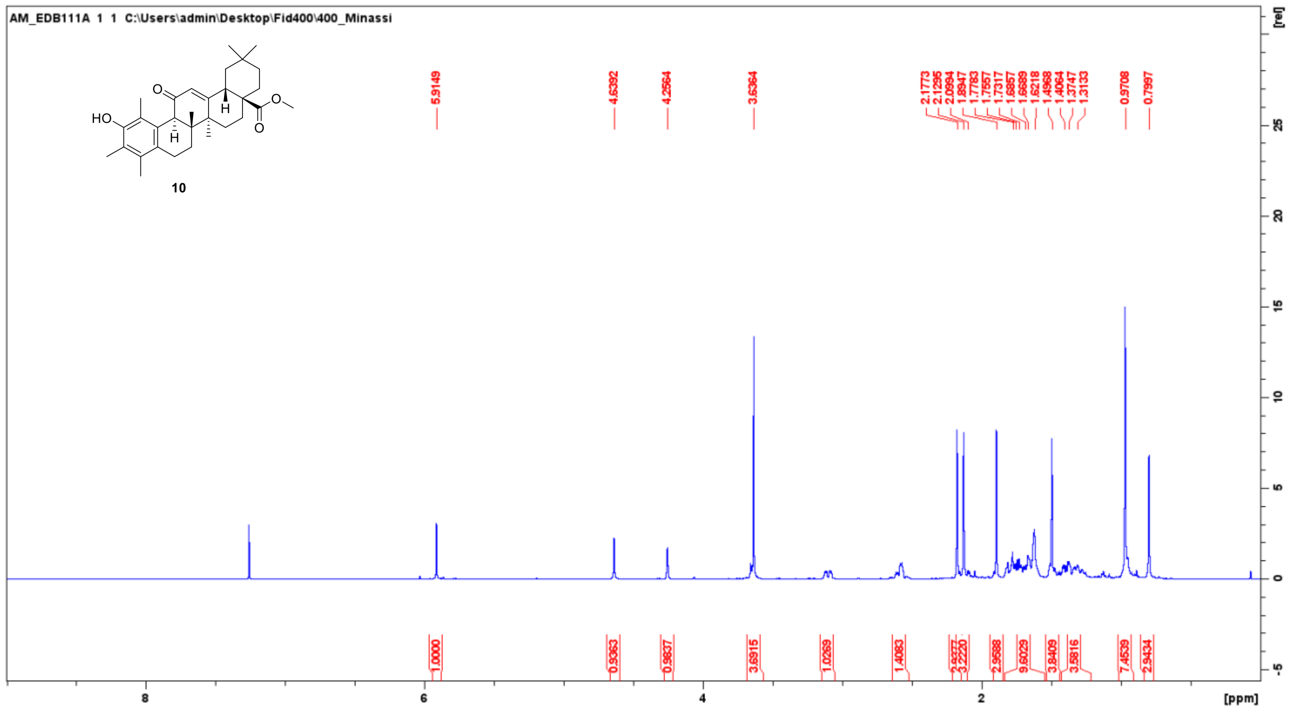

1.30 ^{13}C NMR spectrum of compound 10 (400 MHz, $CDCl_3$)

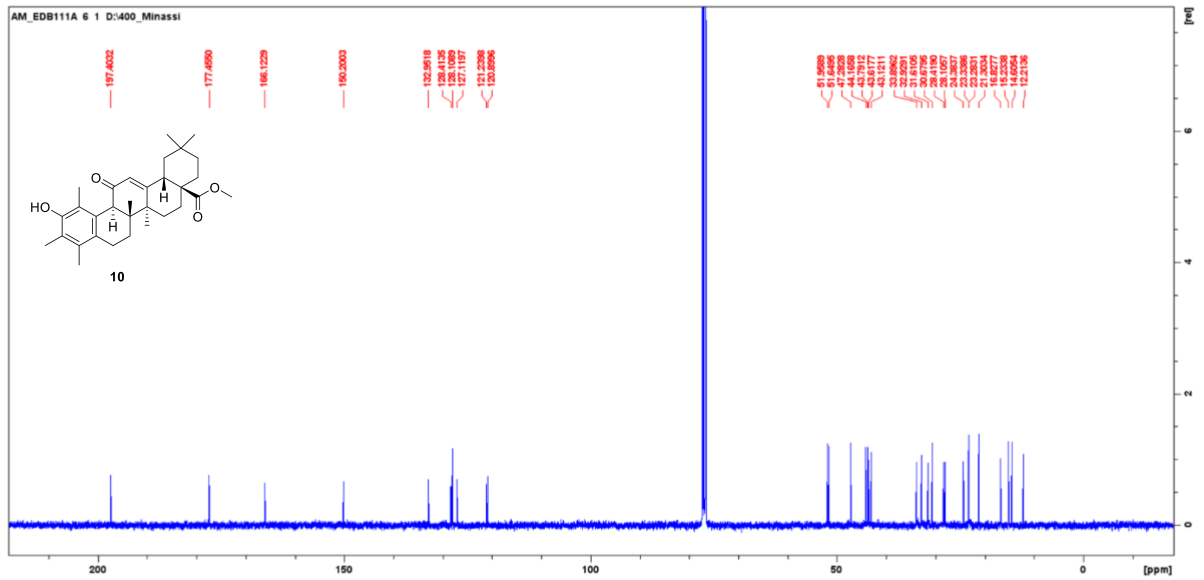

1.31. COSY NMR spectrum of compound 10 (400 MHz, CDCl3)

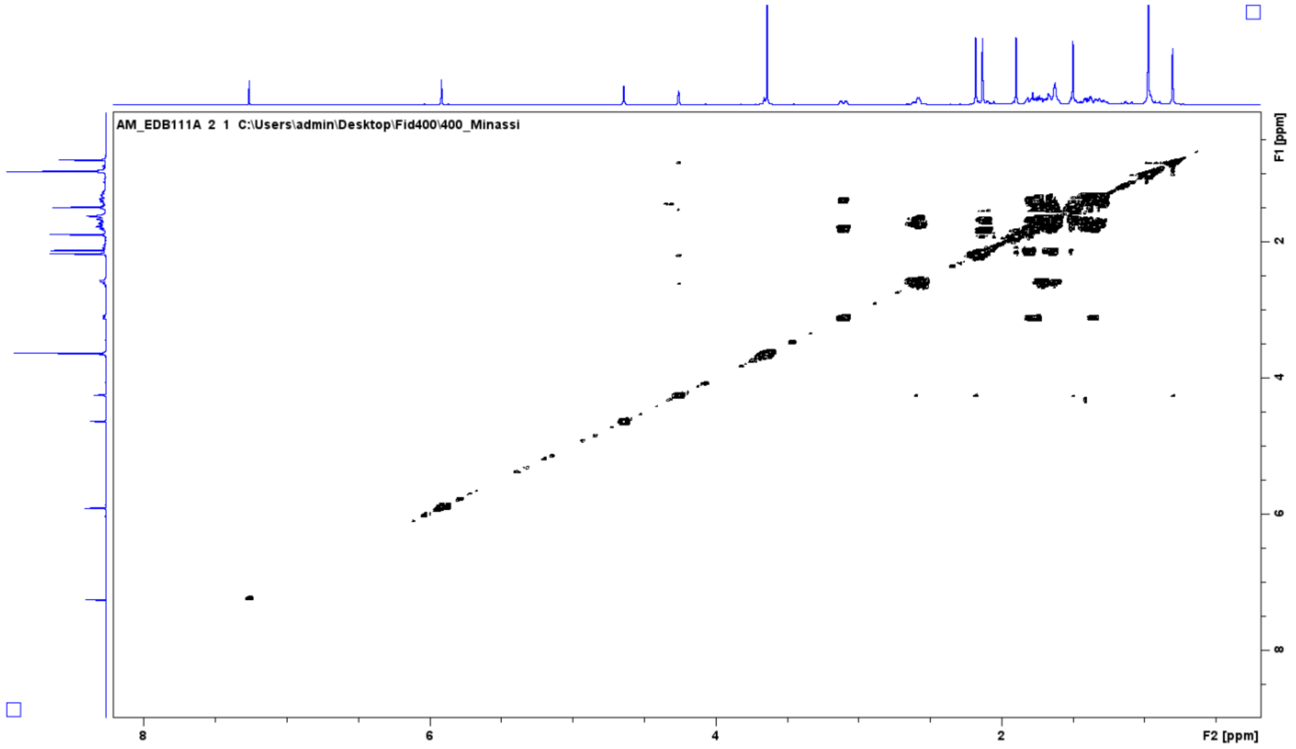

1.32. HSQC spectrum of compound 10 (400 MHz, CDCl3)

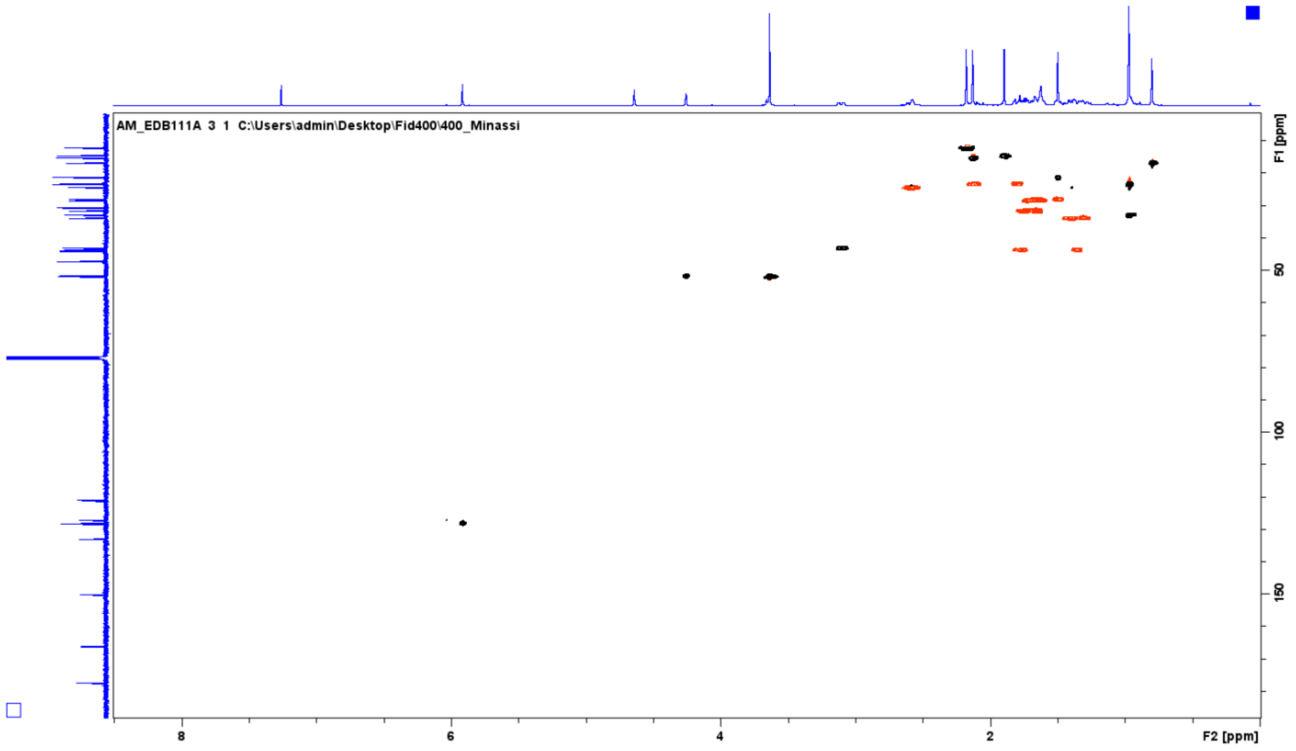

1.33. HMBC spectrum of compound 10 (400 MHz, CDCl$_3$)

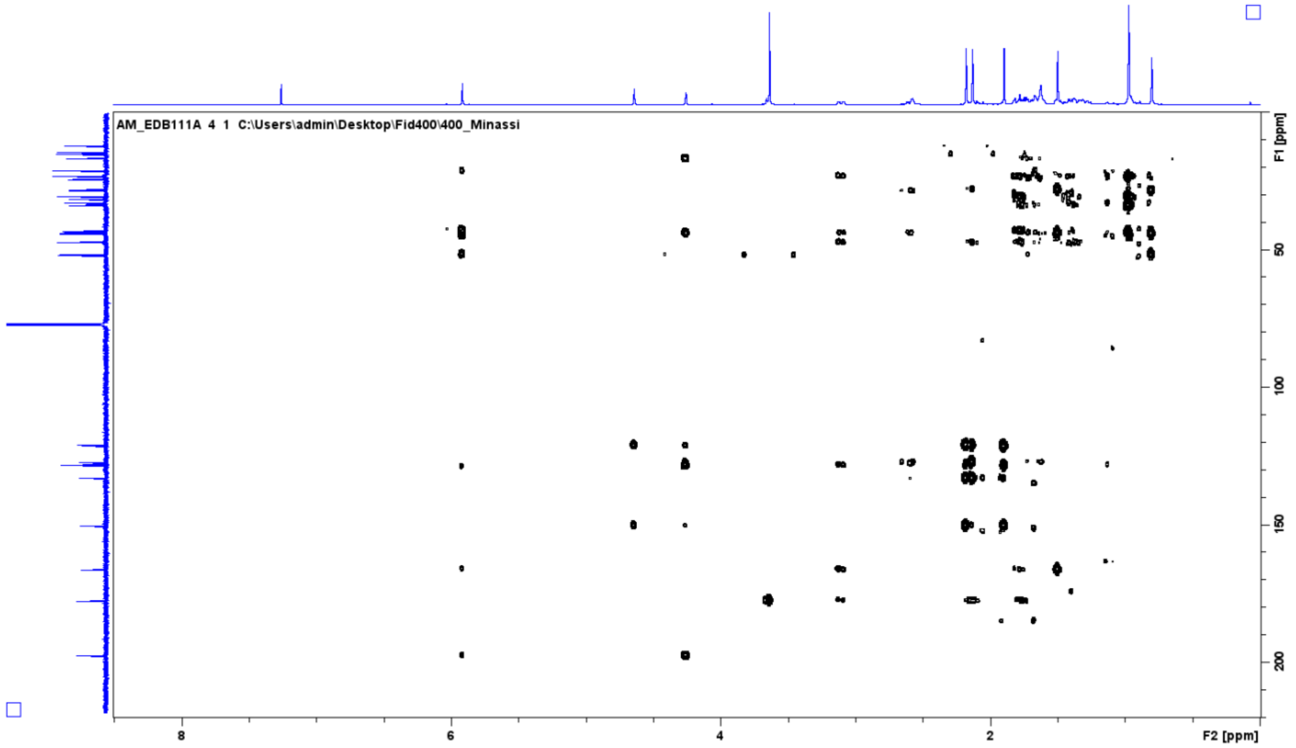

1.34. NOESY spectrum of compound 10 (400 MHz, CDCl$_3$)

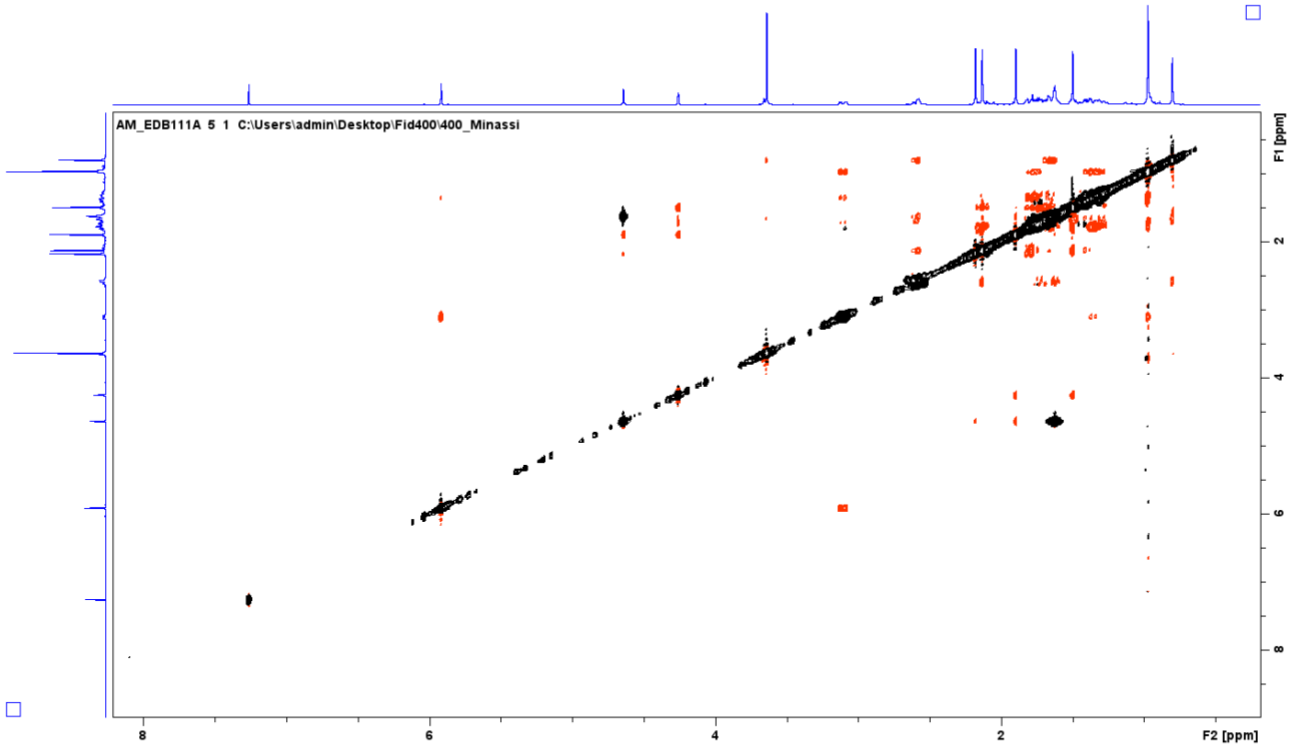

1.35. ^{1}H NMR spectrum of compound 5a (400 MHz, $CDCl_3$)

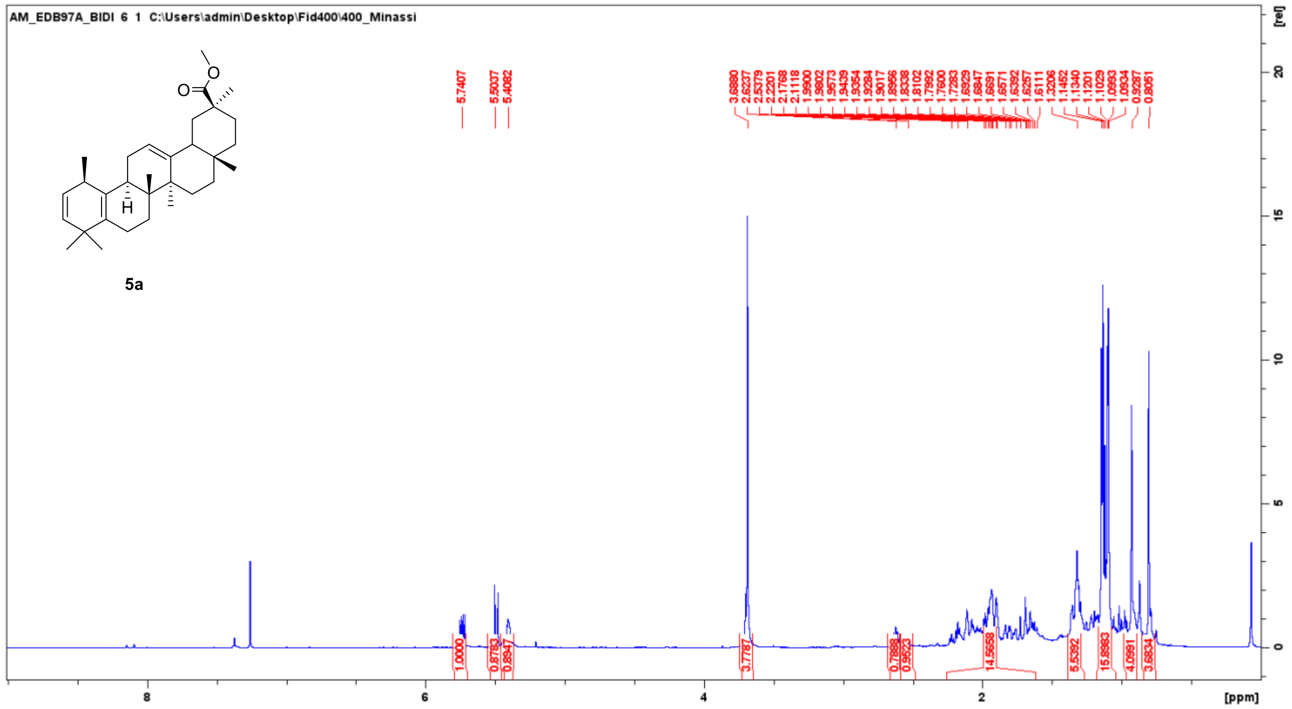

1.36 ^{13}C NMR spectrum of compound 5a (400 MHz, $CDCl_3$)

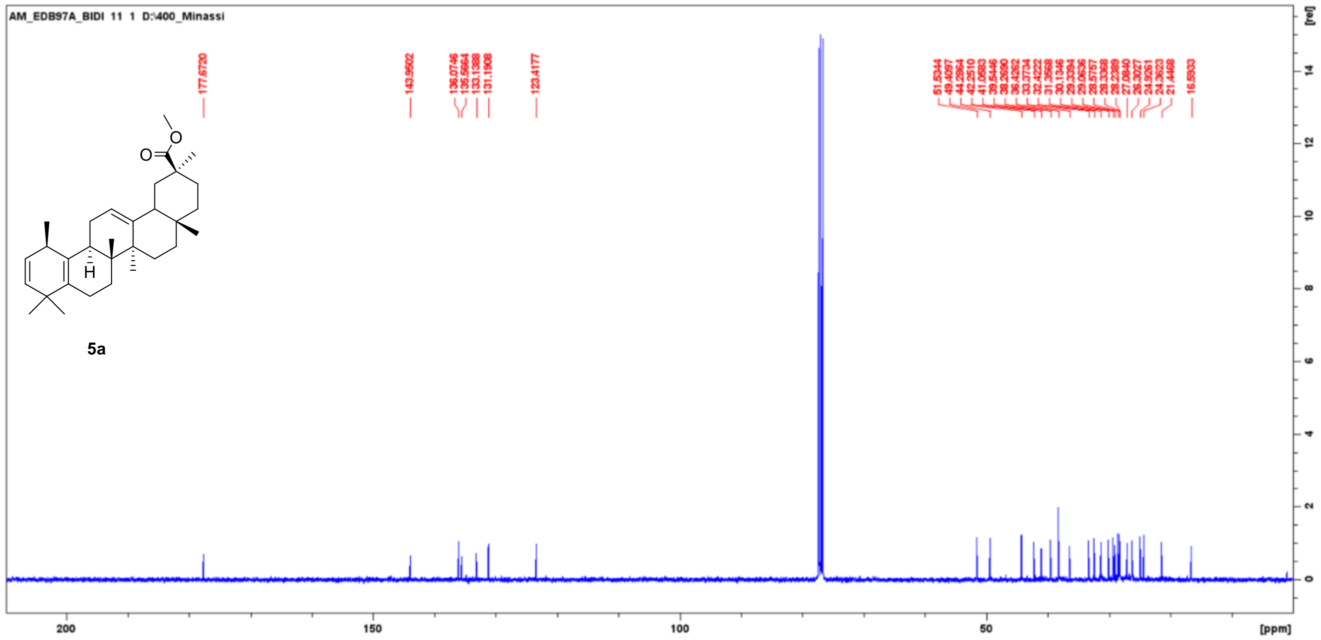

1.37. COSY spectrum of compound 5a (400 MHz, $CDCl_3$)

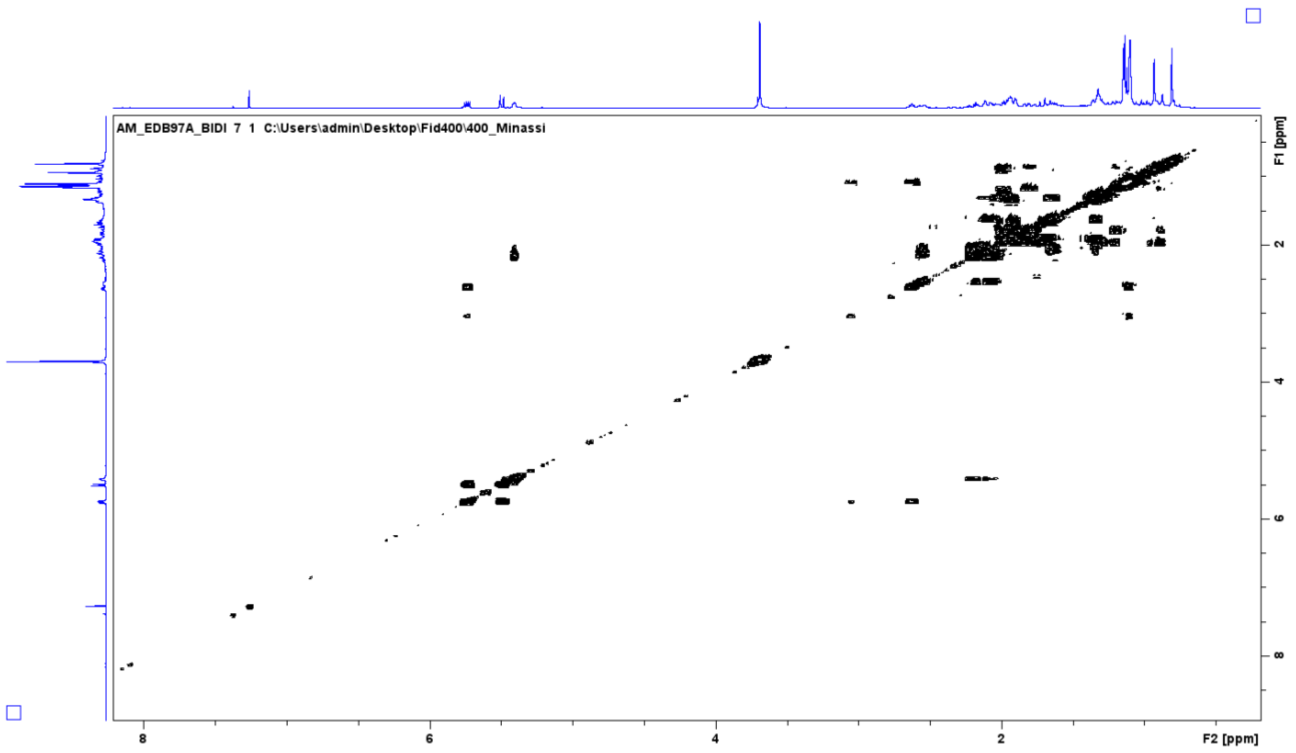

1.38. HSQC spectrum of compound 5a (400 MHz, $CDCl_3$)

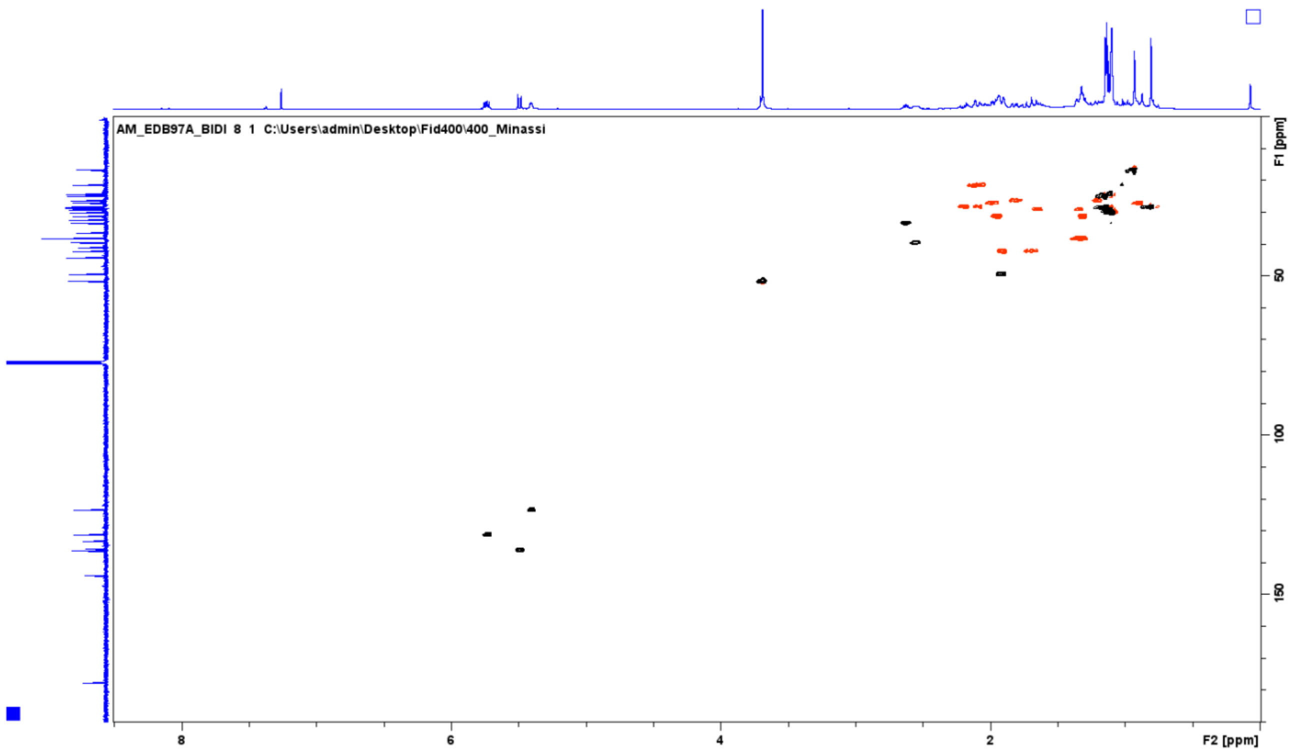

1.39. HMBC spectrum of compound 5a (400 MHz, $CDCl_3$)

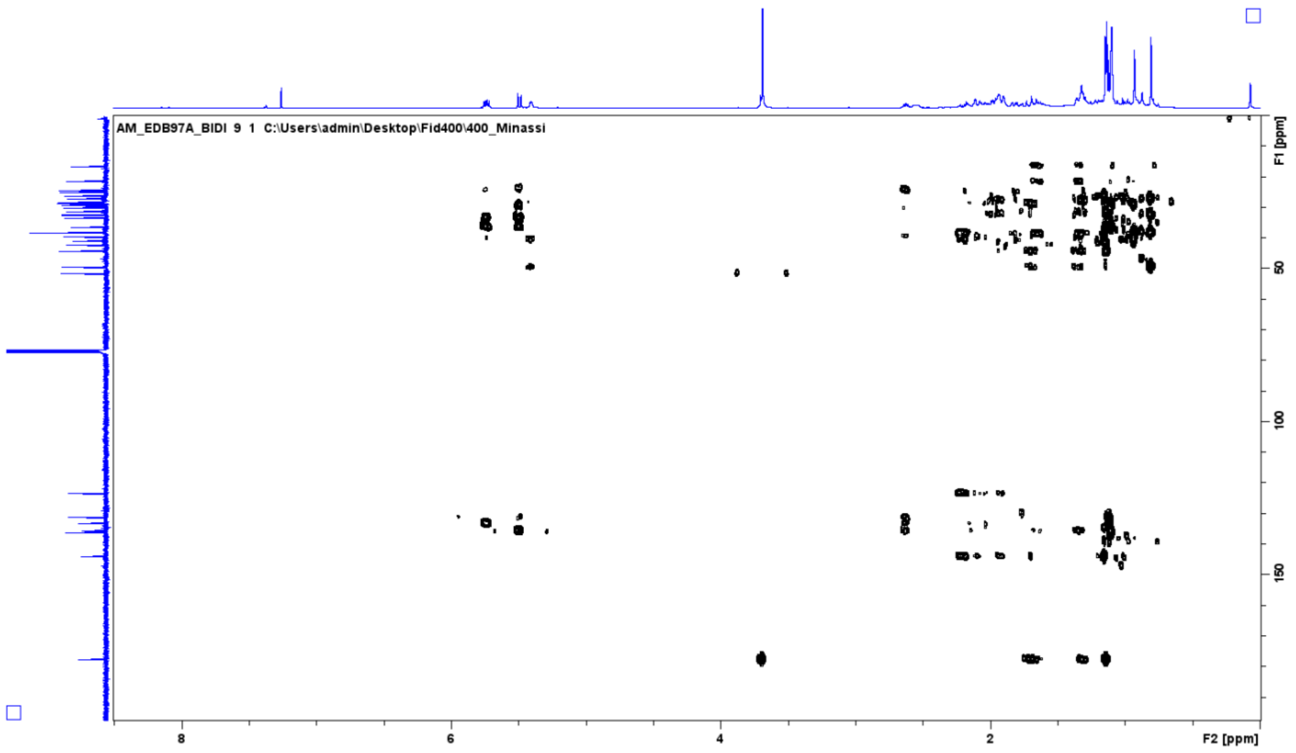

1.40. NOESY spectrum of compound 5a (400 MHz, $CDCl_3$)

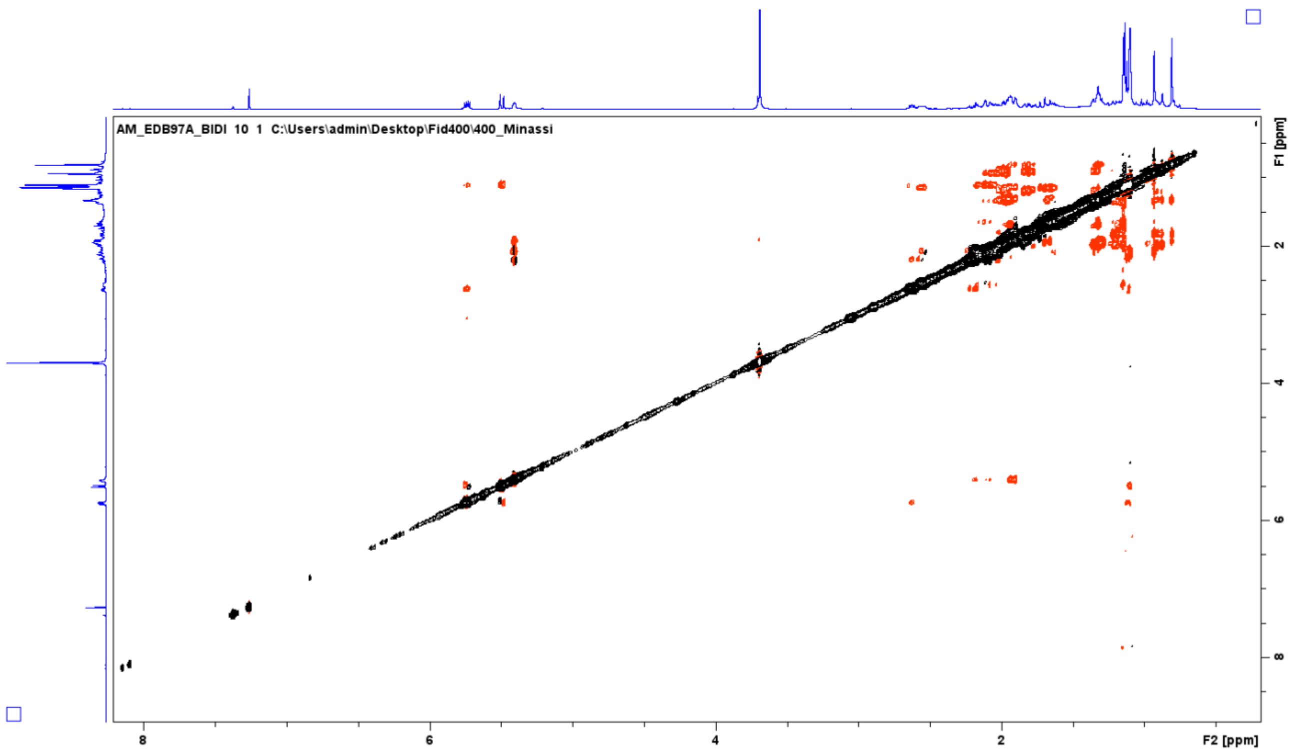

1.41. ^{1}H NMR spectrum of compound 6a (400 MHz, $CDCl_3$)

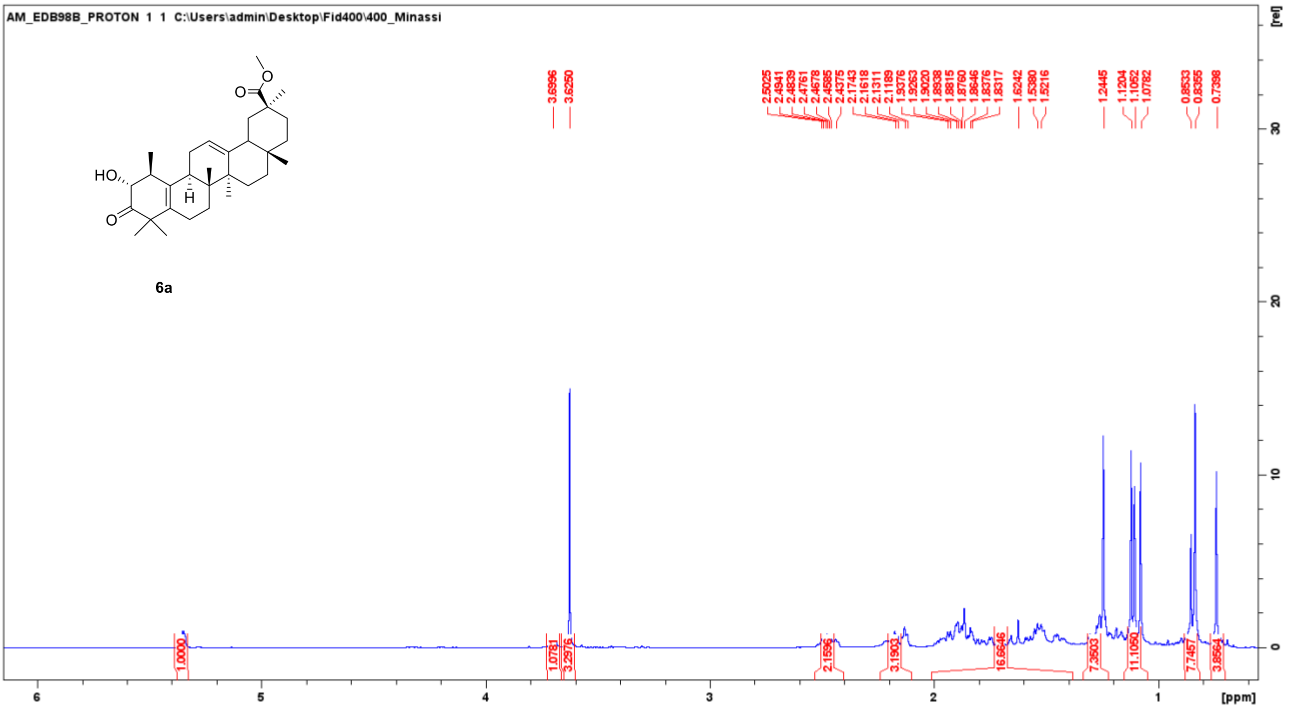

1.42. ^{13}C NMR spectrum of compound 6a (400 MHz, $CDCl_3$)

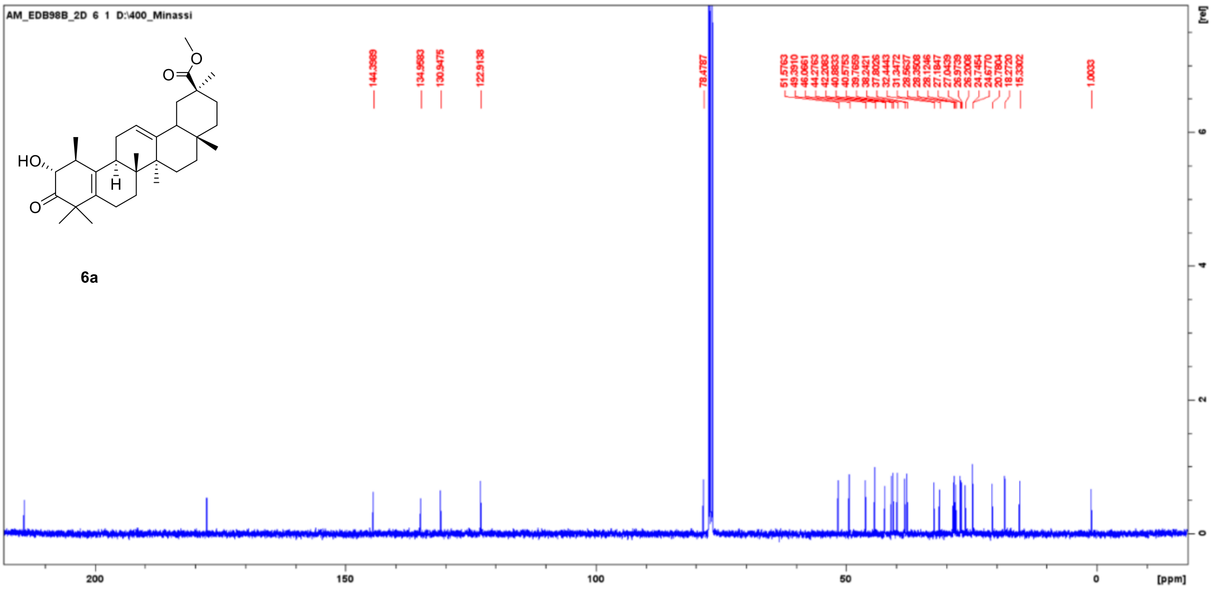

COSY NMR spectrum of compound 6a (400 MHz, $CDCl_3$)

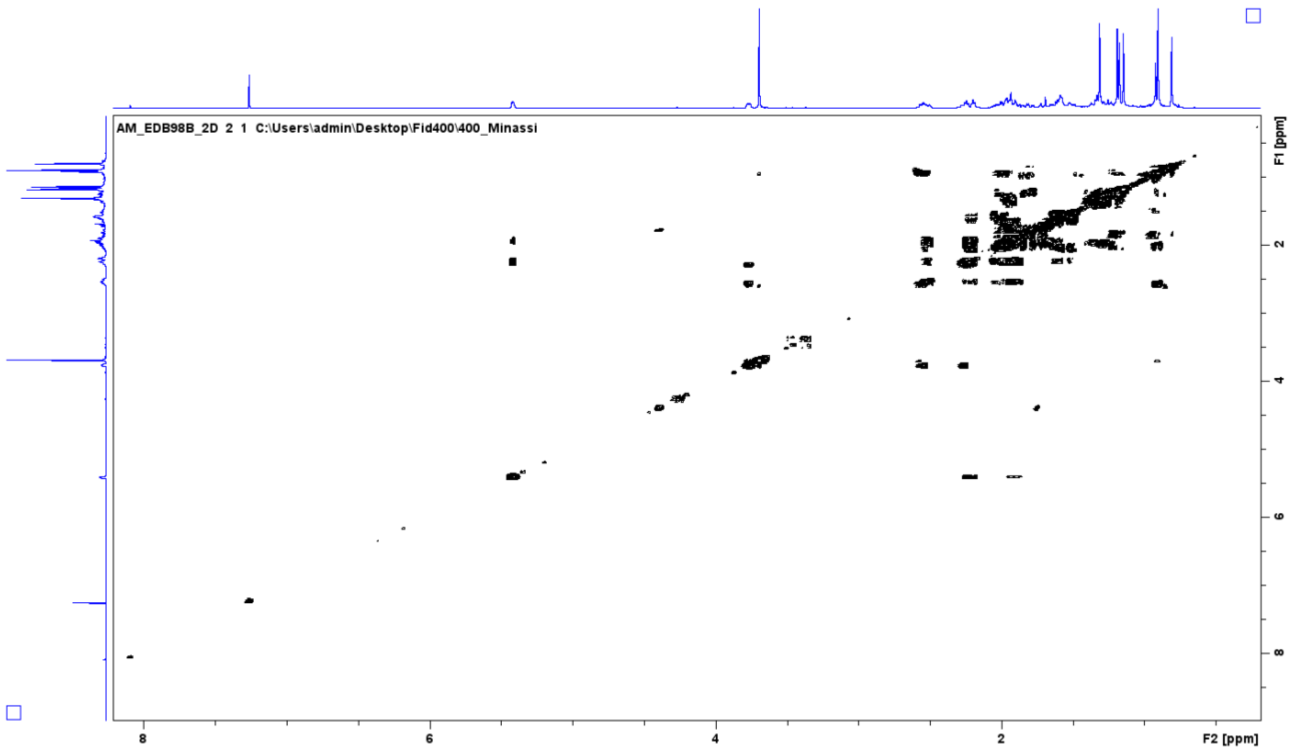

1.43 HSQC NMR spectrum of compound 6a (400 MHz, $CDCl_3$)

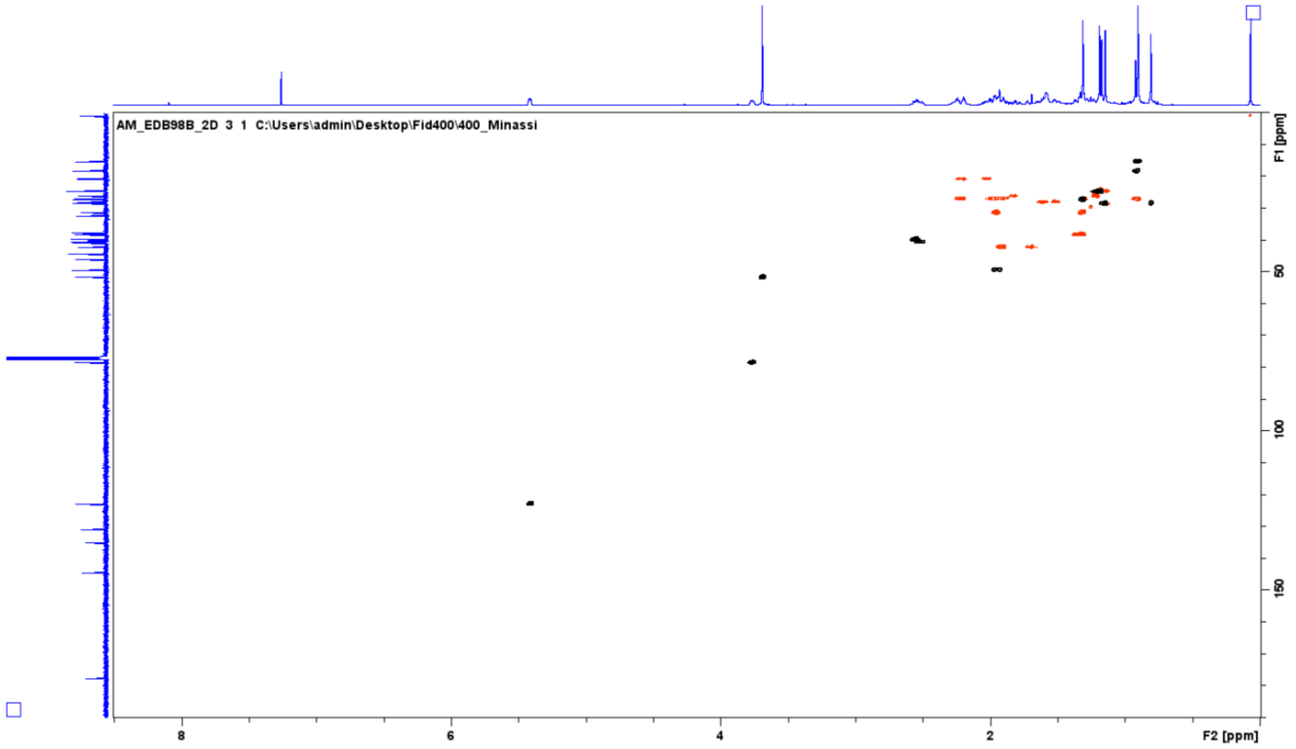

1.44. HMBC NMR spectrum of compound 6a (400 MHz, CDCl3)

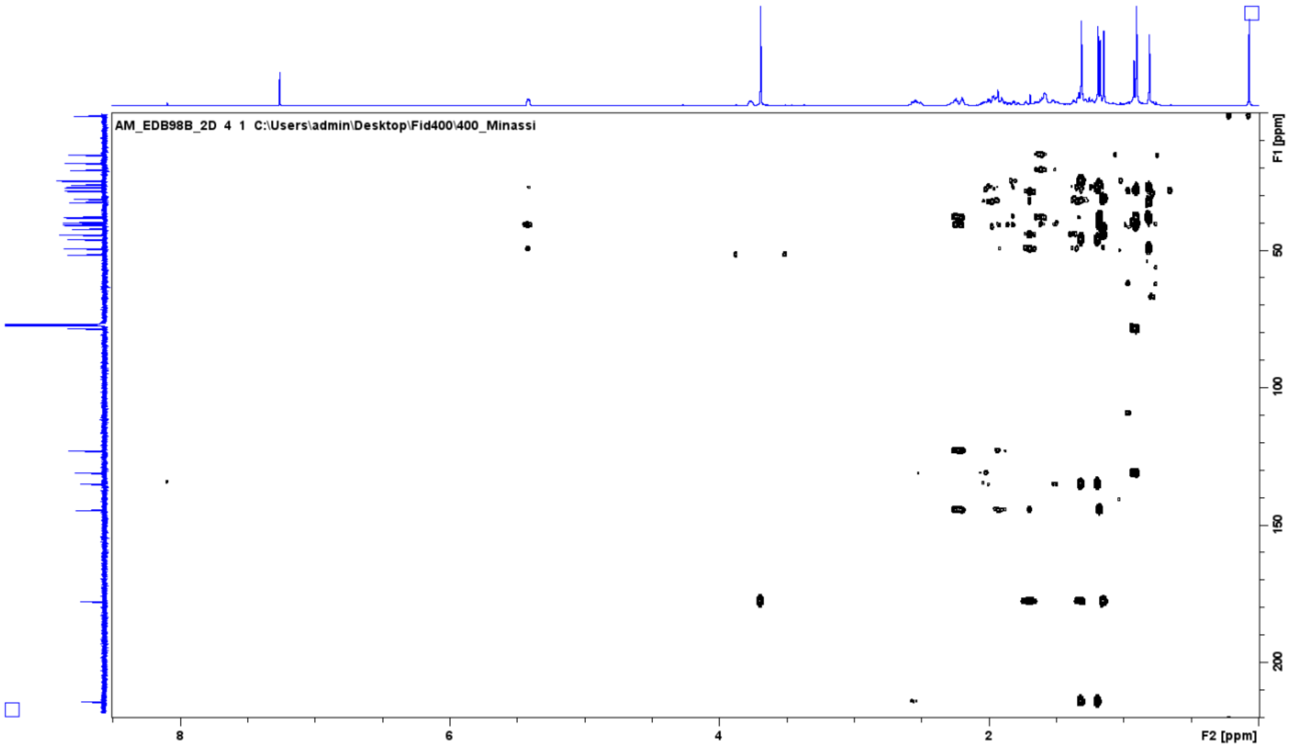

1.46 NOESY NMR spectrum of compound 6a (400 MHz, CDCl3)

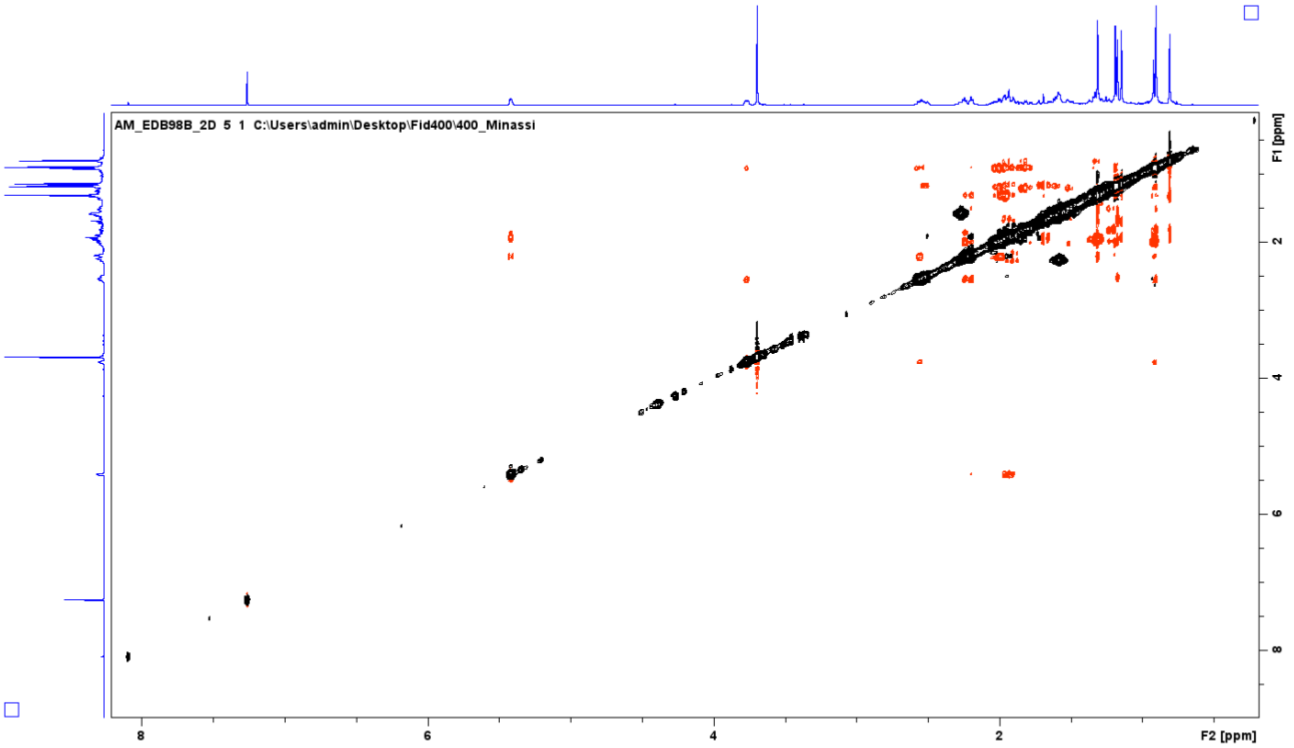

1.47. ^{1}H NMR spectrum of compound 7a (400 MHz, $CDCl_3$)

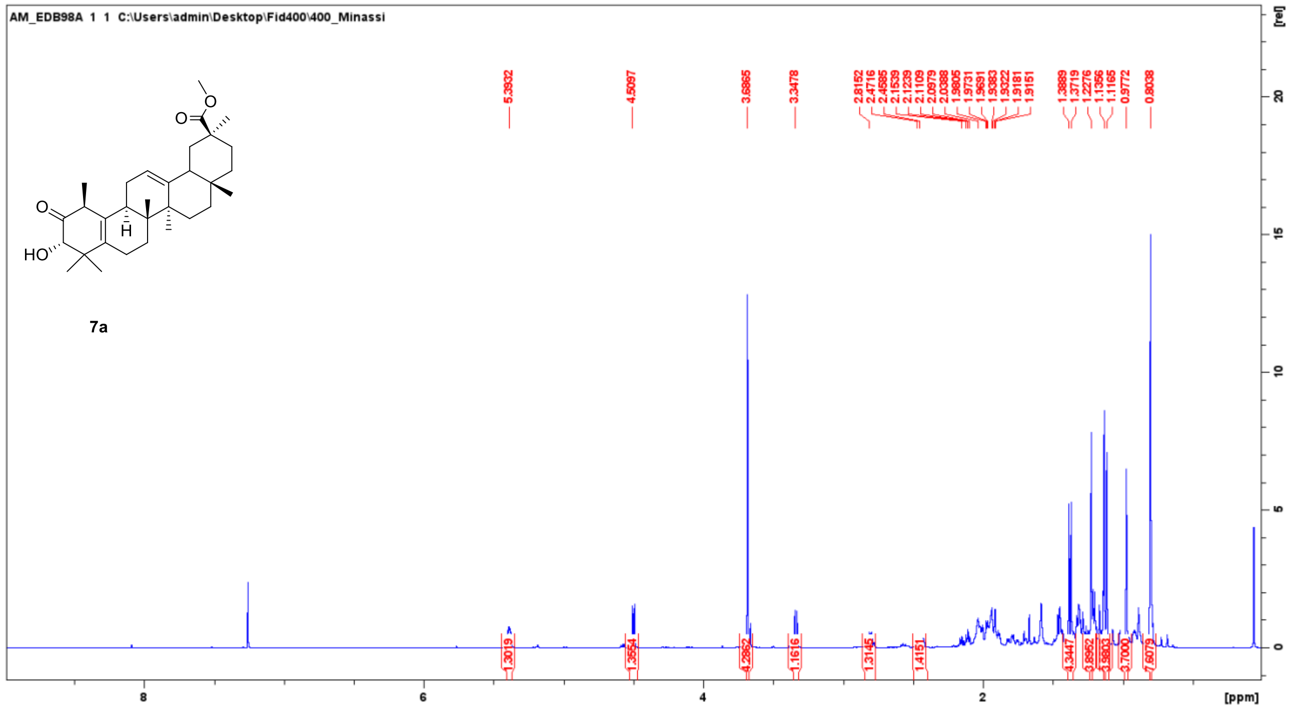

1.48. ^{13}C NMR spectrum of compound 7a (400 MHz, $CDCl_3$)

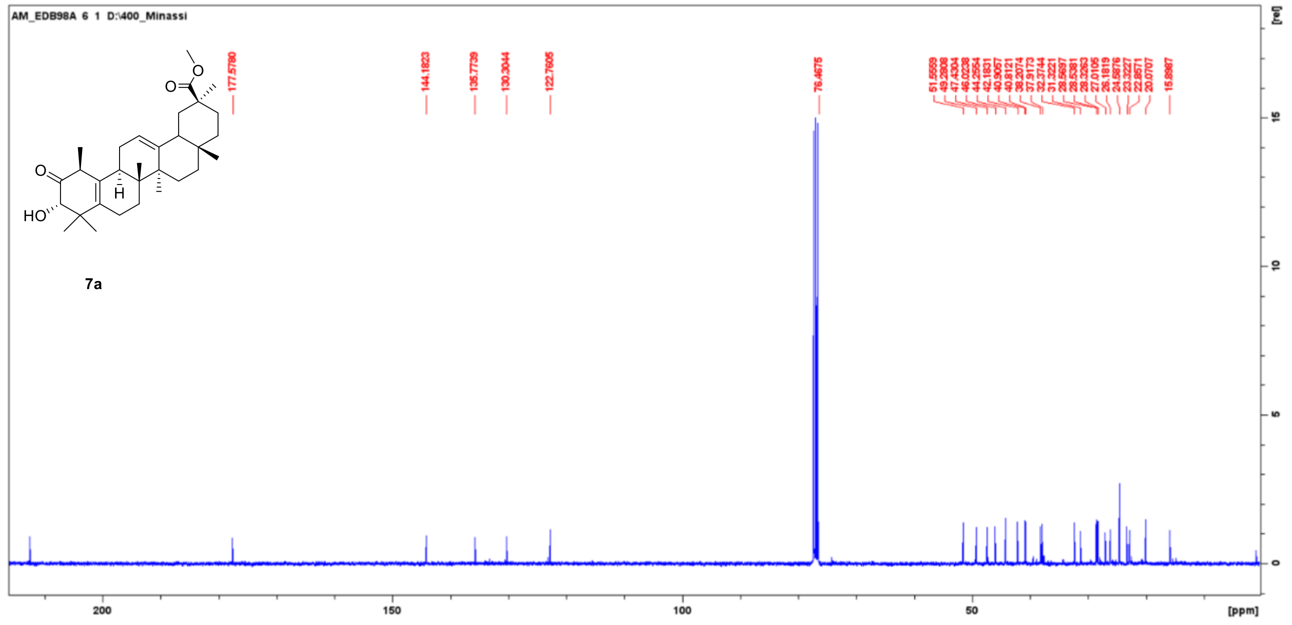

1.49 COSY NMR spectrum of compound 7a (400 MHz, CDCl3)

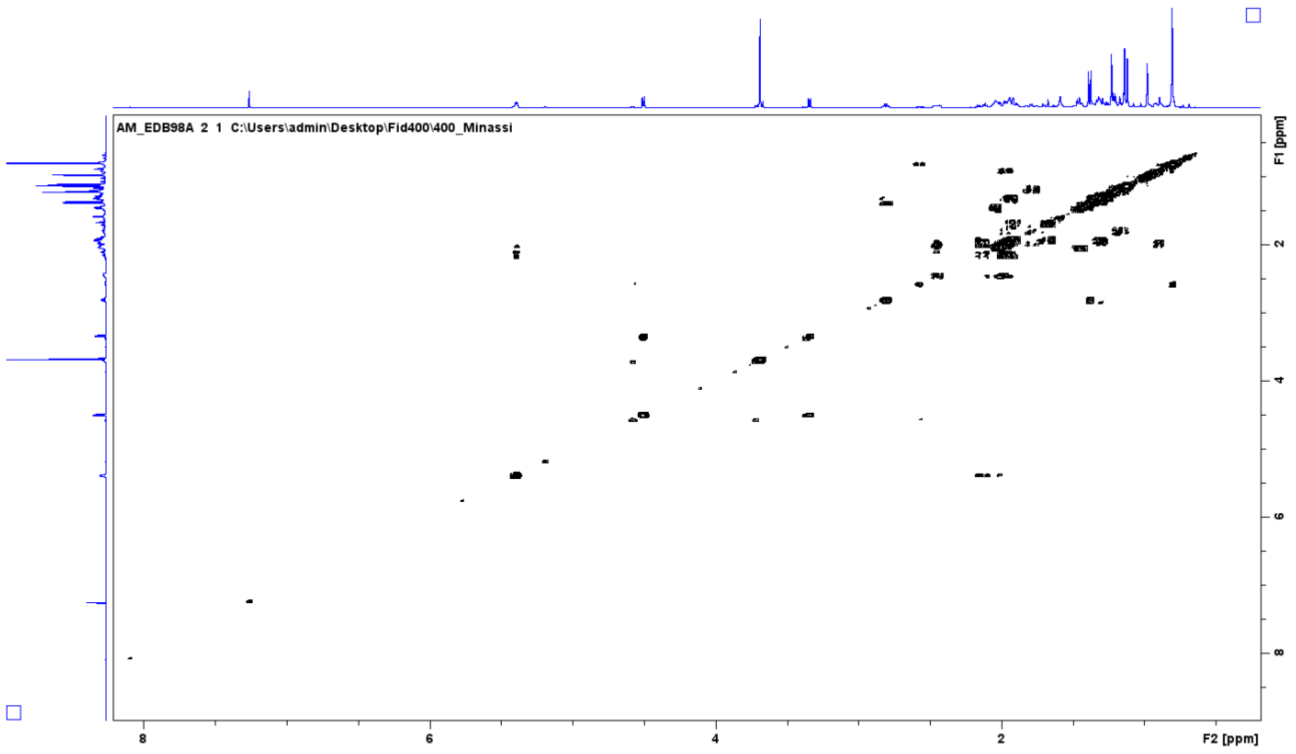

1.50 COSY NMR spectrum of compound 7a (400 MHz, CDCl3)

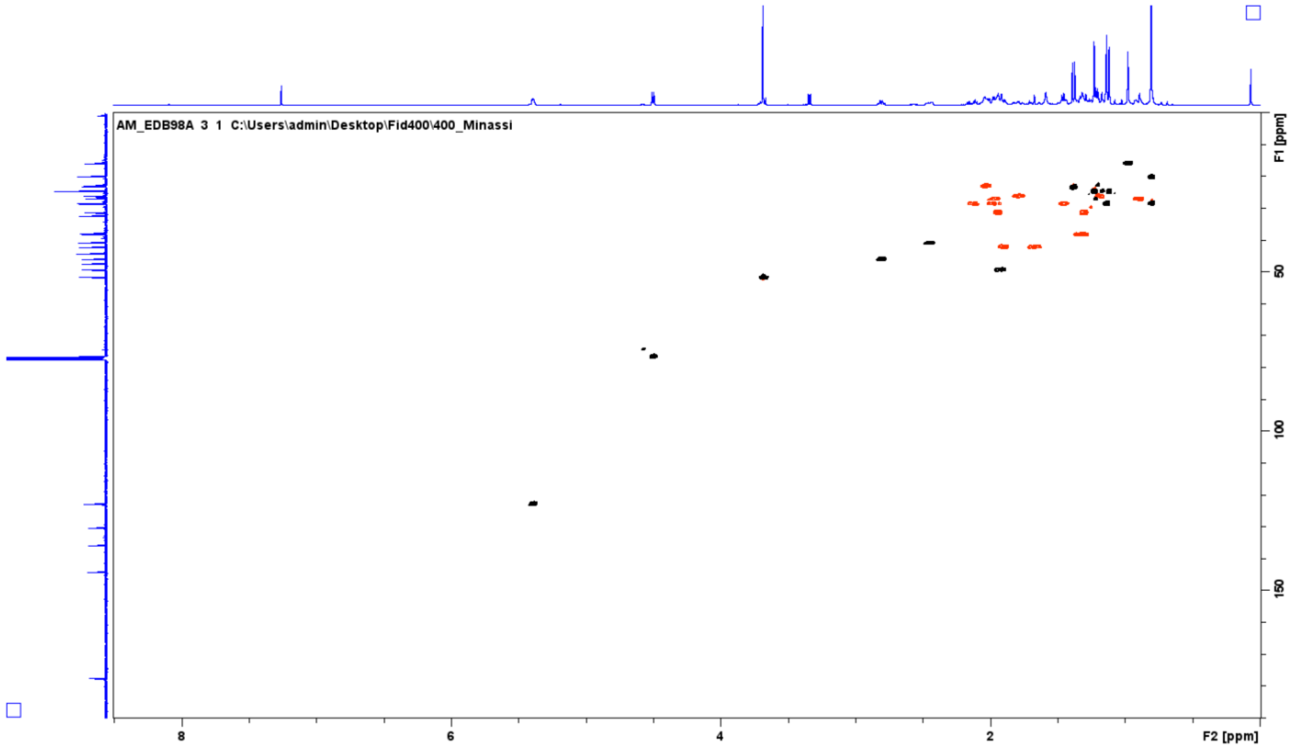

1.51.HMBC NMR spectrum of compound 7a (400 MHz, CDCl₃)

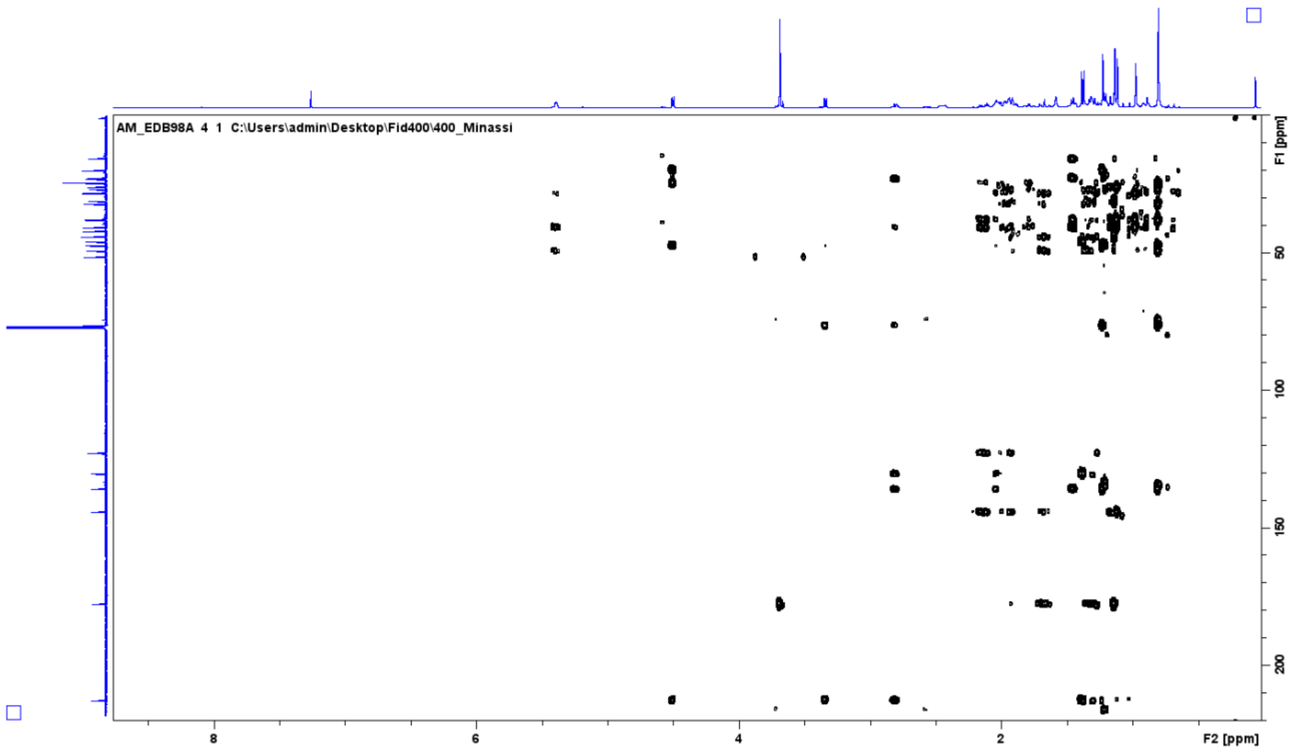

1.52 NOESY NMR spectrum of compound 7a (400 MHz, CDCl₃)

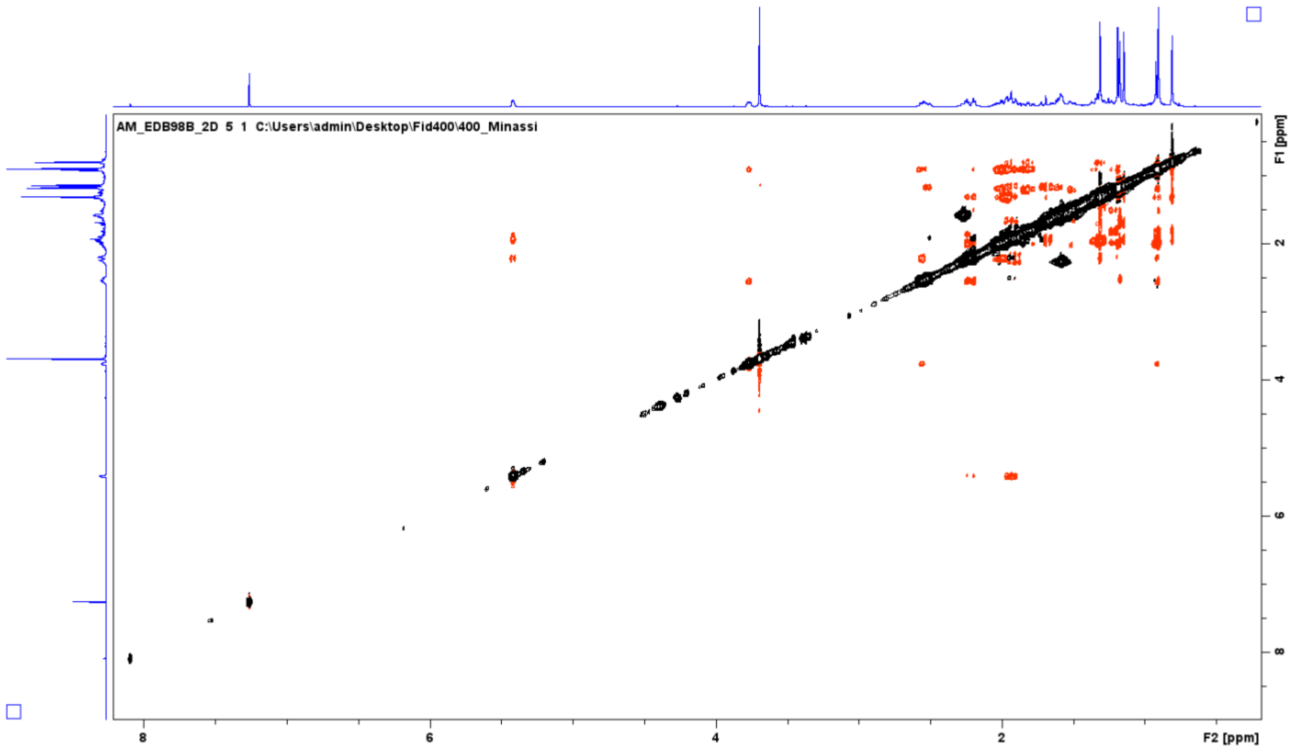

1.53 ^{1}H NMR spectrum of compound 10a (400 MHz, $CDCl_3$)

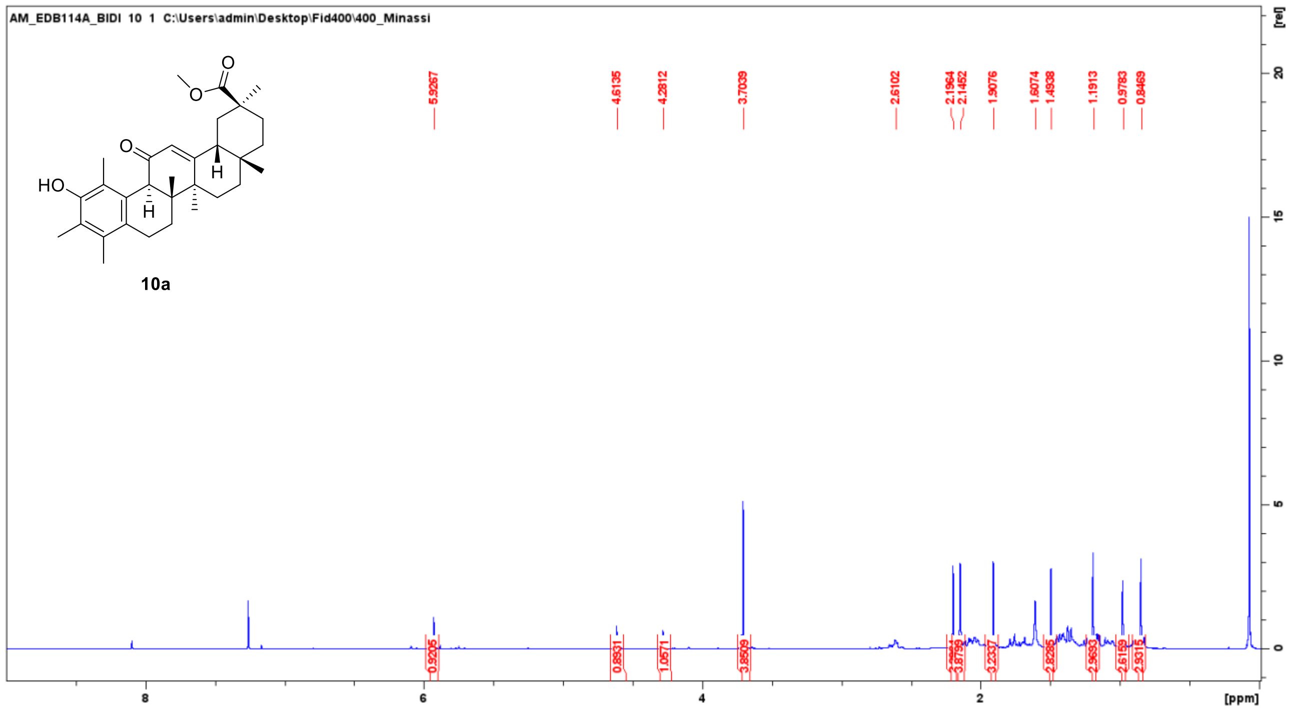

1.54 ^{13}C NMR spectrum of compound 10a (400 MHz, $CDCl_3$)

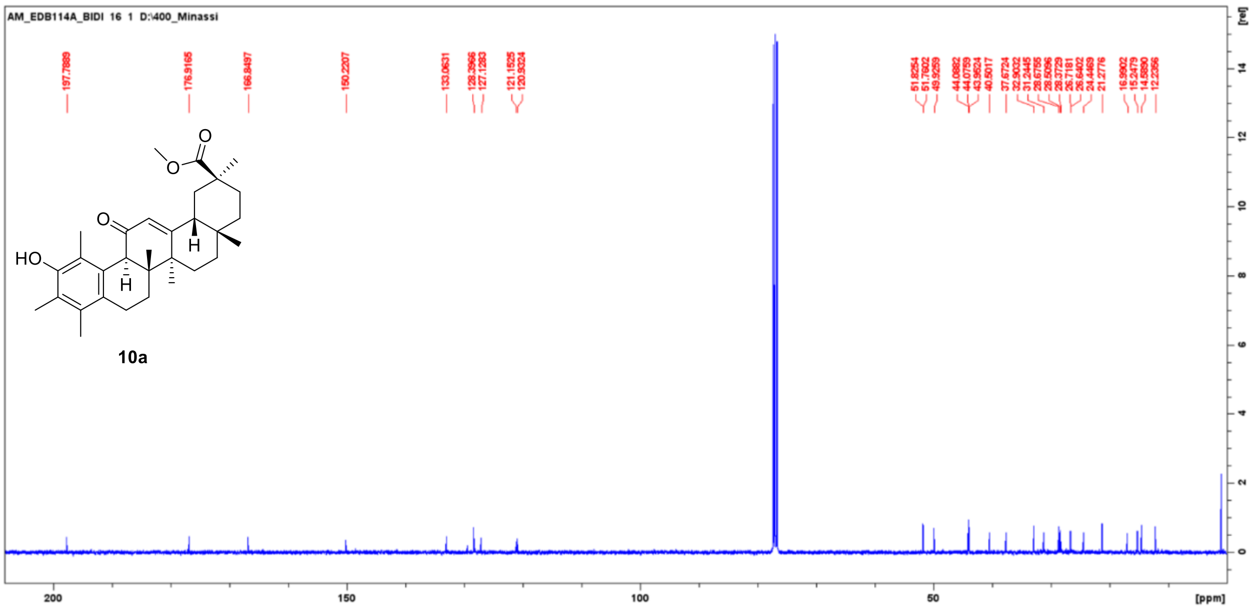

1.55 COSY NMR spectrum of compound 10a (400 MHz, $CDCl_3$)

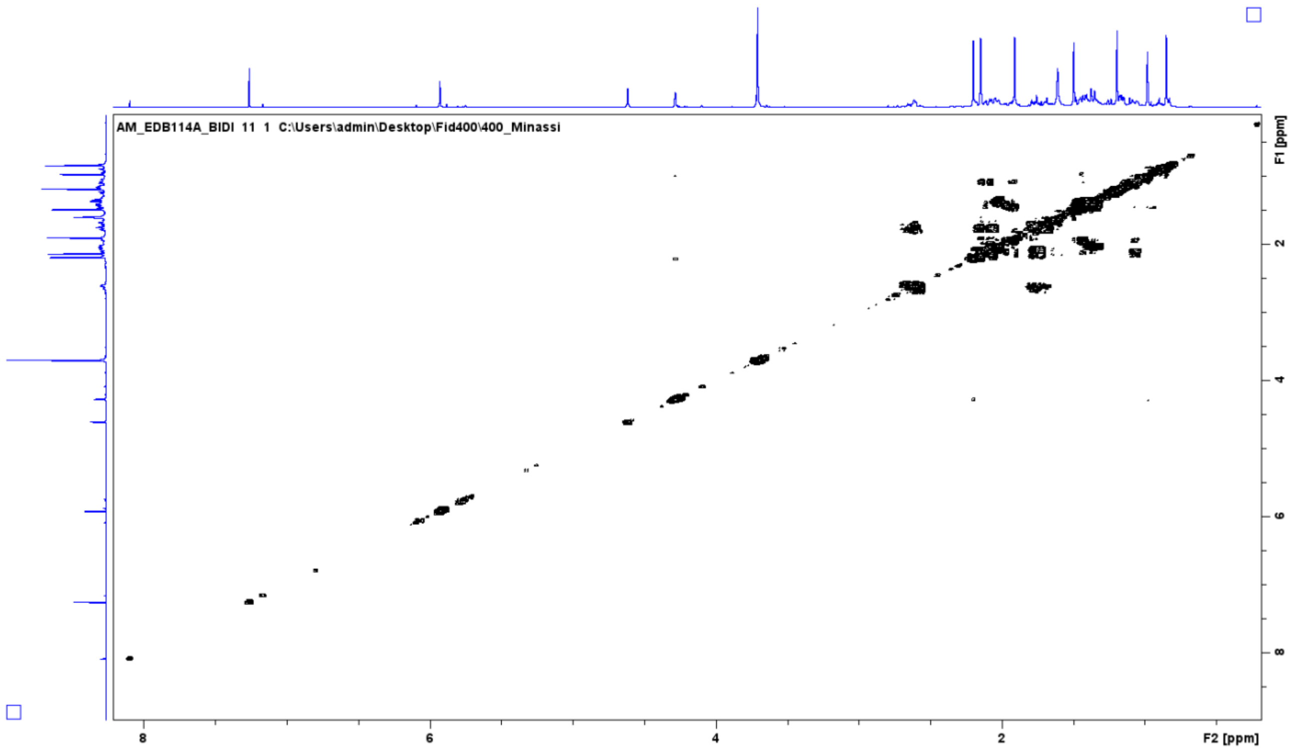

1.56 HSQC NMR spectrum of compound 10a (400 MHz, $CDCl_3$)

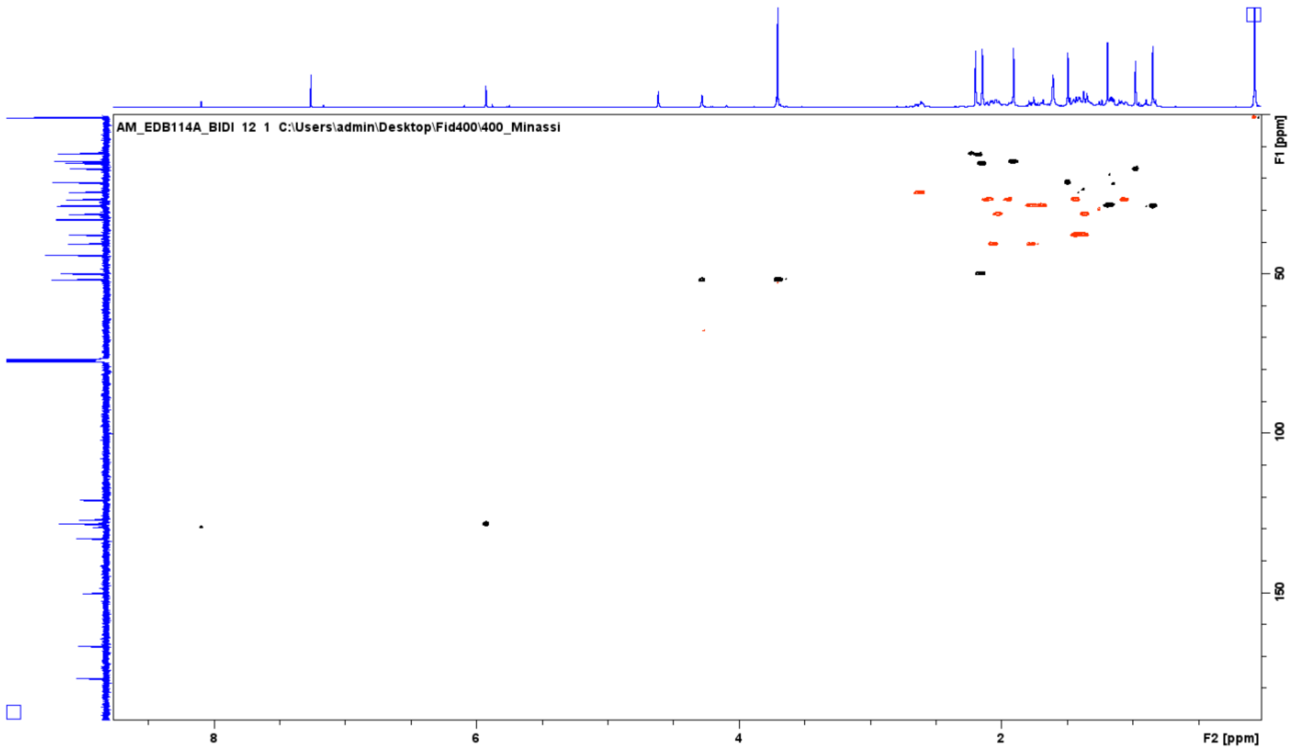

1.57 HMBC NMR spectrum of compound 10a (400 MHz, $CDCl_3$)

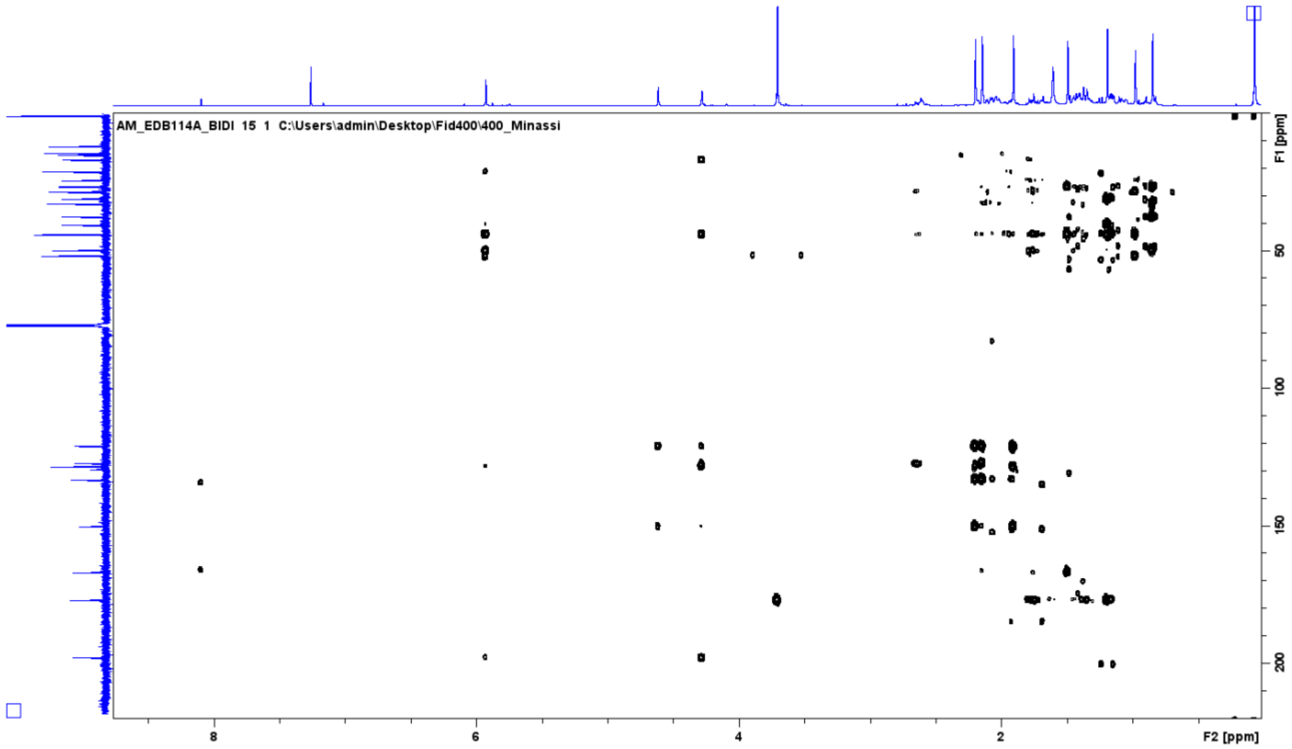

1.58 NOESY NMR spectrum of compound 10a (400 MHz, $CDCl_3$)

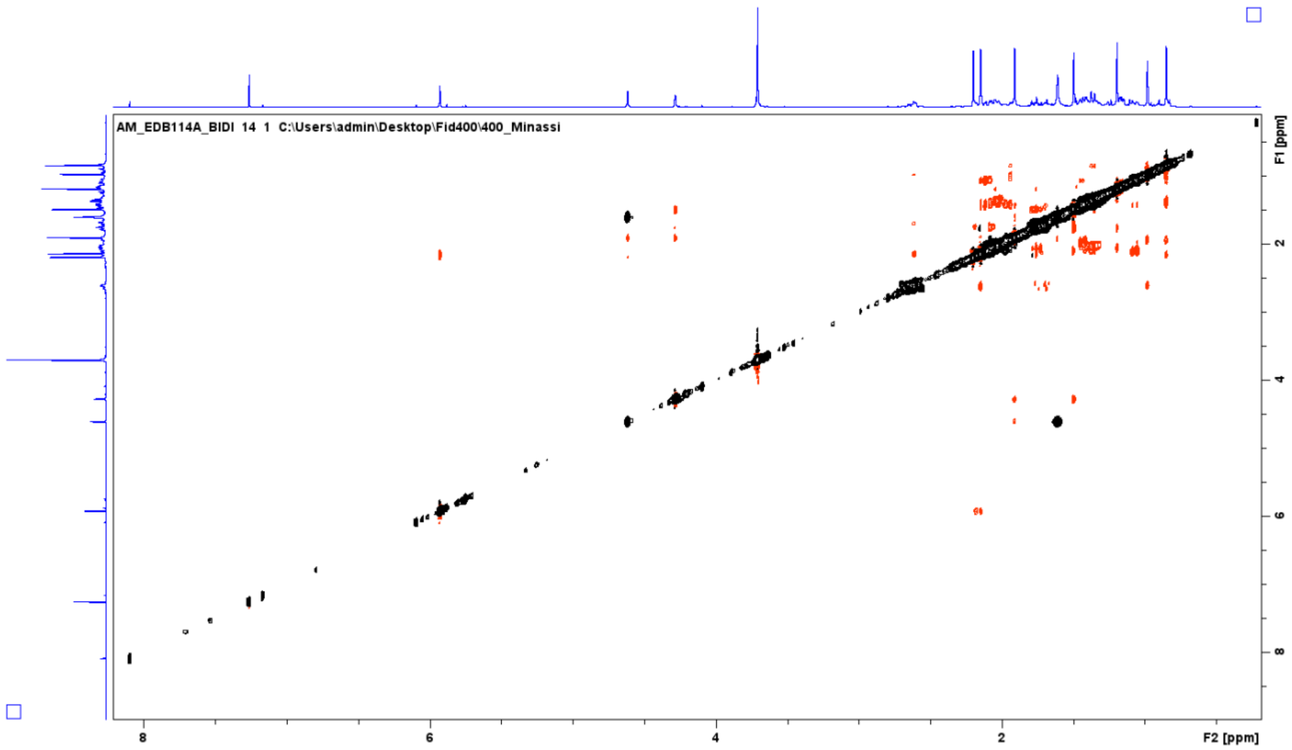

Supplement: Supplementary file 1 [file ol5c02231_si_001.pdf]
